# Insight into the Structure of Victorin, the Host-Selective Toxin from the Oat Pathogen *Cochliobolus victoriae*. Studies of the Unique Dehydroamino Acid β-Chlorodehydroalanine

**DOI:** 10.1021/acs.jafc.3c01387

**Published:** 2023-07-24

**Authors:** Karolina Banaś, Paweł Lenartowicz, Monika Staś, Błażej Dziuk, Dawid Siodłak

**Affiliations:** 1 Faculty of Chemistry, University of Opole, Oleska 48, 45-052 Opole, Poland; 2 Faculty of Chemistry, Wroclaw University of Science and Technology, Wybrzeze Wyspianskiego 27, 50-370 Wroclaw, Poland; 3 Faculty of Chemistry, University of Wroclaw, Joliot-Curie 14, Wroclaw 50-383, Poland

**Keywords:** conformational analysis, amino acid synthesis, Ramachandran diagrams, N−H···Cl hydrogen bond, photoisomerization, dehydroamino acids

## Abstract

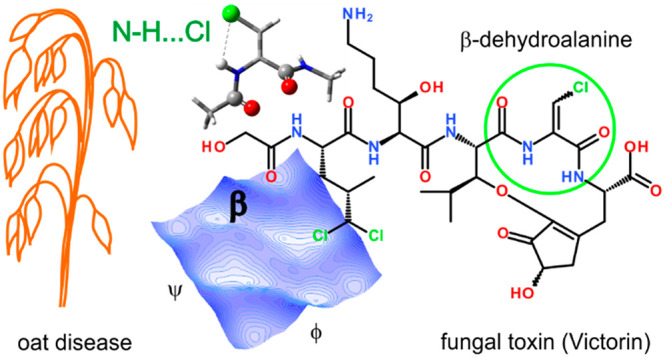

Victorins, a family of peptide toxins, produced by the fungal pathogen *Cochliobolus victoriae* and responsible for disease of some oat varieties, contain a β-chlorodehydroalanine residue, ΔAla(βCl). To determine the conformational properties of this unique dehydroamino acid, a series of model compounds was studied using X-ray, NMR, and FT-IR methods, supported by theoretical calculations. The ΔAla(βCl) geometrical isomers differ in conformational profile. The isomer *Z* prefers the helical conformation α (φ, ψ = −61°, −24°), PPII type conformation β (φ, ψ = −47°, 136°), and semiextended conformation β2 (φ, ψ = −116°, 9°) in weakly and more polar solutions. The isomer *E* prefers mainly the extended conformation C5 (φ, ψ = −177°, 160°), but with an increase of the environment polarity also conformations β (φ, ψ = −44°, 132°) and α (φ, ψ = −53°, −39°). In the most stable conformations the N-H···Cl hydrogen bond (5^γ^) occurs, created between the chlorine atom of the side chain and the N-H donor of the flanking amide group. The method of synthesis of the β-chlorodehydroalanine residue is proposed, by chlorination of dehydroalanine and then the photoisomerization from the isomer *Z* to *E*. The presented results indicate that the assignment of the geometrical isomer of the ΔAla(βCl) residue in naturally occurring victorins still remains an open question, despite being crucial for biological activity.

## Introduction

**Figure 1:**
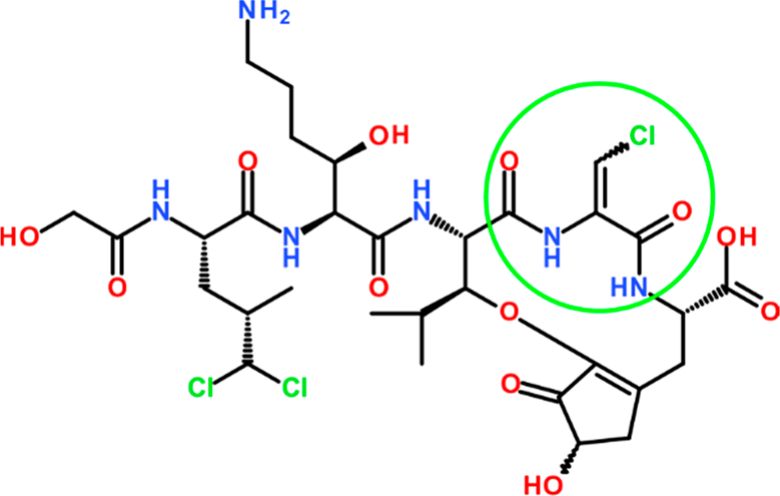
Structure of victorin C, the predominant compound of the family of victorins.^7^ The β-chlorodehydroalanine residue, ΔAla(βCl), is marked, with flanking peptide bonds. The geometry of the Cα=Cβ double bond is debatable (*E*^9^ or *Z*(10)).

The oat, genus *Avena sativa*, is a commercially cultivated plant of considerable economic importance.^1−4^ The necrotrophic fungal pathogen *Cochliobolus victoriae*, producing the host-selective toxins victorins, causes disease of some oat varieties.^5,6^ Victorins are a family of peptides^7−10^ in which victorin C is the predominant compound (Figure 1).

This highly modified peptide does not possess any standard amino acid. Our interest focuses on the β-chlorodehydroalanine residue, ΔAla(βCl), as it is common for all compounds of the victorin family, but so far it was not detected in other naturally occurring compounds. As a conservative unit, one can suppose that β-chlorodehydroalanine plays an important role in the bioactivity of victorins. β-Chlorodehydroalanine belongs to dehydroamino acids, which in general constitute many naturally occurring peptides^11^ and are classified as nonstandard amino acids. Their characteristic structural feature is the π-bond between carbon atoms α and β. The carbon–carbon double bond is responsible for the specific reactivity of the dehydroamino acid residue, for example, by bonding toxin peptide via Michael addition.^12^ The presence of the Cα=Cβ bond results in the lack of asymmetric carbon α and loss of the optical activity of dehydroamino acid residue. On the other hand, it creates the possible geometrical isomers *Z*/*E*, which may be of key importance for the biological action of the peptide. This is shown by the example of biologically active cyclic pentadepsipeptide phomalide and the inactive isophomalide analogue.^13^

The conformational properties of the most common dehydroamino acids—dehydroalanine,^14^ dehydrobutyrine,^15^ and dehydrophenylalanine^16^—are well recognized. Dehydroalanine, having the simplest methylidene side chain, is prototypical for the whole family of dehydroamino acids. Dehydrobutyrine, with the β-methyl group, is the simplest dehydroamino acid, which reveals geometrical isomers *Z*/*E*. Dehydroalanine and dehydrobutyrine are the most common naturally occurring dehydroamino acids. Dehydrophenylalanine, with the β-phenyl group, mostly the isomer *Z*, is often used in peptide design.

The conformational properties of the β-chlorodehydroalanine were not studied before. The presence of the bulky chlorine atom in the side chain can considerably influence the conformational preferences of the amino acid residue. Not only the chlorine atom can impose a steric hindrance, but also the presence of a heteroatom in the side chain, with lone pairs of electrons, is likely to cause a specific interaction, both intramolecular in the peptide and intermolecular with the target protein or solvent. Determining the conformational properties of β-chlorodehydroalanine should provide deeper insight into the biological effects of victorins. In addition, it gives the possibility of using β-chlorodehydroalanine as a specific tool in peptide design in order to obtain desired properties.

## Materials and Methods

### Computational Procedures

Analyses of short model compounds is a common approach in evaluation of the conformational properties of the selected amino acid residue.^17,18^ The model compounds Ac-(*Z*)-ΔAla(βCl)-NHMe (**1**) and Ac-(*E*)-ΔAla(βCl)-NHMe (**2**) were calculated by the DFT method using the Gaussian 16 package.^19^ The initial structures were prepared with GaussView5.^20^ The configurations *trans* (ω_0_ ≈ 180°) of both amide groups were set. The potential energy surfaces *E* = *f*(φ,ψ) were obtained at M06-2X/6-31+G(d,p) level of theory,^21^ in the gas phase, and then in chloroform and water (constrained optimization). The values of the φ and ψ dihedral angles were changed in steps of 30°. Because of the achirality of dehydroamino acids, each structure has a mirror counterpart with the same energy but opposite torsion angles (φ, ψ = −φ, −ψ), which reduced the number of grid point structures of the maps to 91 each. The solvent effect was simulated with the self-consistent reaction field (SCRF) using the conductor-like polarizable continuum model (CPCM).

All potential energy minima localized in the maps were fully optimized by using a bigger basis set, 6-311+G(d,p). Unconstrained optimizations were followed by vibrational analysis to ensure that the resulting structures are true energy minima and to obtain the zero-point vibrational energies (ZPVEs) and Gibbs energies (298.15 K, 1.0 atm). The population of the conformations (*p*) was calculated at the 300 K temperature, where *RT* = 0.595 kcal/mol according to the following equations:^22,23^
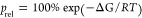
1and
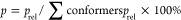
2The names of the conformations are based on the Scarsdale nomenclature.^24^

The XYZ structures of the calculated compounds, Ac-(Z)-ΔAla(βCl)-NHMe (**1**) and Ac-(E)-ΔAla(βCl)-NHMe (**2**), along with their energies are given in Table 1S.

### Crystal Structure Analysis

The single crystals of the studied molecules, Ac-(Z)-ΔAla(βCl)-NHMe (**1**), Ac-(*E*)-ΔAla(βCl)-NHMe (**2**), Cbz-Gly-(Z)-ΔAla(βCl)-Gly-OMe (**3**), Boc-(Z)-ΔAla(βCl)-OMe (**4**), and Ac-ΔAla(βCl_2_)-NHMe (**5**), were collected on a Rigaku Oxford Diffraction XtaLAB SynergyR DW diffractometer equipped with a HyPix ARC 150° Hybrid Photon Counting (HPC) detector using CuKα (λ = 1.54184 Å) at 100 K. The corrections to the Lorentz and polarization factors were applied to the reflection intensities.^25^ Data were processed using the CrysAlisPro software.^26^ The structures were solved by direct methods using SHELXS and refined by the full-matrix least-squares methods based F^2^ using SHELXL.^27,28^ All non-hydrogen atoms were located from difference Fourier synthesis and refined by the least-squares method in the full-matrix anisotropic approximation. The crystallographic data for compounds and details of the X-ray experiment are collected in the Supporting Information. The structure drawings in Supporting Information were prepared by using the Mercury program.^29^ The coordinates of atoms and other parameters for structures were deposited with the Cambridge Crystallographic Data Centre: 2236959 for (**1**), 2236963 for (**2**), 2236961 for (**3**), 2236960 for (**4**), 2236962 for (**5**); 12 Union Road, Cambridge CB2 1EZ, UK (Fax,_44-(1223)336-033, E-mail: deposit@ccdc.cam.ac.uk).

### FTIR Spectra

A Nicolet Nexus 2002 FT-IR spectrometer flushed with dry nitrogen during the measurements was used. The thickness of the KBr liquid cell was 0.01 mm. For each measurement 20 scans were accumulated with 2 cm^–1^ resolution (spectral resolution 0.482 cm^–1^) in the spectral range 400–4000 cm^–1^. The Ac-(*Z*)-ΔAla(βCl)-NHMe (**1**) and Ac-(*E*)-ΔAla(βCl)-NHMe (**2**) solutions were prepared in chloroform in three different concentrations: 0.5, 1.0, and 2.0 mg/mL each. The spectral processing and peak deconvolution were conducted using the Fityk software;^30^ applying the voigt function.

### NMR Spectra

The NMR analyses were performed on a Bruker Ultrashield 400 (Bruker 2005) spectrometer with Bruker software (TopSpin Version 1.3), operating at 400 MHz for ^1^H and 101 MHz for ^13^C. The spectra were recorded in (CD_3_)_2_SO (DMSO-*d*_6_) or CDCl_3_, CD_3_OD, or D_2_O (internal TMS standard) at room temperature. To determine the isomers, the NOE difference method was applied using the standard programs.

### Synthesis—General Procedure

For the detailed synthetic procedure and characterization of each compound, please see the Supporting Information.

### Chlorination of Dehydroalanine

Chlorination of dehydroalanyl residue was performed on a millimolar scale (0.3–0.6 mmol) based on a protocol adapted from the literature^31−33^ with some modifications, including the use of triethylamine as a base. The peptide substrate was dissolved in a DMF/DCM (1:8) solvent mixture. Then, a solution of chlorine gas in DCM was added dropwise to the reaction mixture to the point at which a pale yellow color appeared. The volatile components were quickly evaporated under reduced pressure. The residue was resuspended in DCM, triethylamine (2.5 equiv) was added, and the reaction mixture was stirred for 15 min. The reaction mixture was concentrated and directly purified by flash column chromatography. The desired products were crystallized from an EtOAc/hexane solvent system.

### Photoisomerization of β-Chlorodehydroalanine

Photoisomerization reaction of the β-chlorodehydroalanyl residue was performed on a millimolar scale (0.04–0.2 mmol) in a quartz cuvette. The peptide substrate and benzophenone (5 equiv) were dissolved in a methanol/benzene (1:0.4) solvent mixture. The reaction mixture was stirred and illuminated with UV light (366 nm) with a power density of 400–440 μW/cm^2^ for five hours. Then, the reaction mixture was evaporated under reduced pressure and directly purified by flash column chromatography. The desired products were crystallized from an EtOAc/hexane solvent system.

## Results and Discussion

### Theoretical Method

#### Ac-(*Z*)-ΔAla(βCl)-NHMe (**1**)

**Figure 2:**
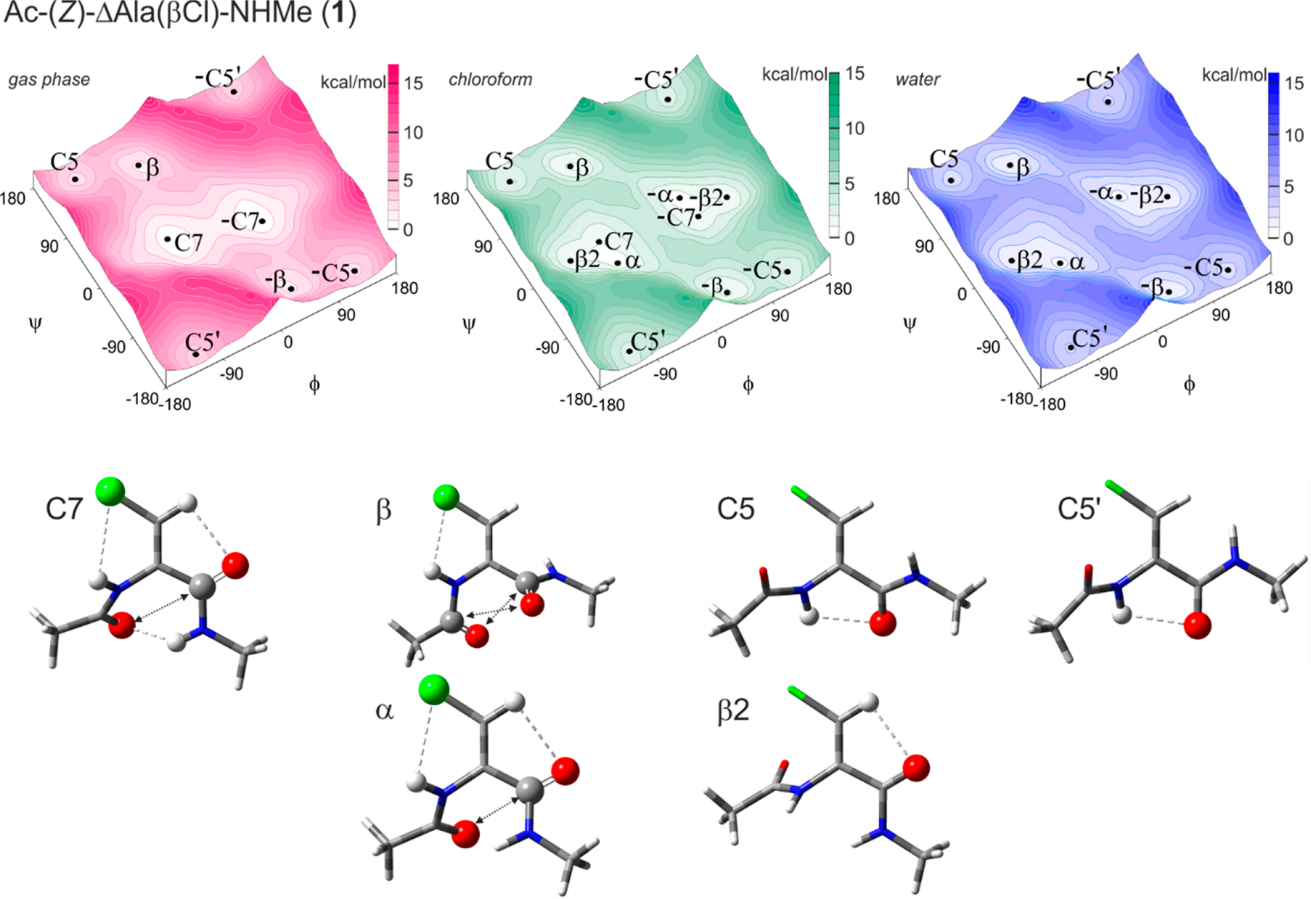
Potential energy surfaces *E* = (φ, ψ) of Ac-(*Z*)-ΔAla(βCl)-NHMe (**1**) obtained by the M06-2X/6-31+G(d,p) method in the gas phase, chloroform, and water environment. Energy contours are plotted every 1 kcal/mol. Below the maps are the conformations optimized at the M06-2X/6-311+G(d,p) level of theory in chloroform with the most important electrostatic interactions (↔)^38^ and hydrogen bonds (···)^41^ created within the residue.

The diamide model compound (**1**) containing the isomer *Z* of the β-chlorodehydroalanine residue was studied using theoretical methods to obtain a general view of its conformational properties. The potential energy maps with the conformations corresponding to local minima are presented in Figure 2. Selected parameters are gathered in Table 1. For the isolated molecule (gas phase, *in vacuo*), four possible conformations—C7, β, C5, and C5′—were found in the map, together with their mirror analogues, which have the same energy, but opposite signs of the torsion angles. The lowest in energy is conformation C7 (φ, ψ = −63°, 20°). The analysis of distances between the atoms within the studied residue indicates possible intramolecular interactions (Table 2S). The N^C^-H···O^N^ hydrogen bond created between the C-terminal N–H group and the N-terminal carbonyl oxygen atom constitutes a 7-membered ring, typical for this conformation. The novelty is the N^N^-H···Cl hydrogen bond.^34,35^ It is created between the N–H group of the N-terminal amide bond and the chlorine atom in the side chain and, thus, is denoted as (5^γ^).^36^ There is also the C^β^-H···O^C^ hydrogen bond, which is stronger in dehydroamino acids, due to the hybridization sp^2^ of the β-carbon atom.^14,37^ It should also be noticed the dipole interaction type I of carbonyl groups.^38−40^ The concomitant intramolecular interactions are the reason why 72% of the molecular population takes conformation C7 in the gas phase. Next in the energy order is conformation β (φ, ψ = −41°, 135°). It is also stabilized, by the N^N^-H···Cl hydrogen bond. Also, the dipole–dipole attraction II type between the carbonyl groups can be considered. This conformation is adopted by about 24% of the molecular population. The highest in energy are two extended conformations C5 (φ, ψ = −128°, 157°) and C5′ (φ, ψ = −125°, −165°), which have an opposite value of the angle ψ. These conformations are stabilized by the N^N^-H···O^C^ hydrogen bond, which creates a 5-membered ring formed between the N-terminal N–H group and the C-terminal oxygen atom. The formation of two extended conformations, C5 and C5′, is due to the energy transition barrier caused by a steric hindrance imposed by the large chlorine atom in position Z of the dehydroamino acid side chain and a repulsion with the electronegative oxygen atom of the N-terminal amide group. Furthermore, the H···H repulsion from the C^β^-H atom and the C-terminal N–H group causes a distortion of the intramolecular N-H···O C5-type hydrogen bonds. This results in two nearly symmetrical positions with respect to the ψ torsional angle.

**Table 1:** Selected Parameters for Conformations of Ac-(*Z*)-ΔAla(βCl)-NHMe (**1**)^a^

Conformation	φ (deg)	ψ (deg)	Δ*G* (kcal·mol^–1^)	*p* (%)
*gas phase*				
C7	–62.6	20.1	0.00	71.69
β	–41.4	135.4	0.65	24.09
C5	–128.2	156.7	1.92	2.85
C5′	–124.9	–165.0	2.35	1.38
*chloroform*				
α	–61.8	–23.1	0.00	40.51
β	–42.7	138.7	0.17	30.44
β2	–116.6	9.8	0.56	15.88
C7	–68.2	16.6	0.85	9.68
C5	–127.7	155.1	1.71	2.31
C5′	–121.0	–157.5	2.10	1.18
*water*				
α	–60.3	–25.0	0.00	49.58
β	–43.6	139.7	0.27	31.57
β2	–116.4	9.4	0.65	16.54
C5	–127.6	154.5	2.09	1.47
C5′	–117.8	–153.7	2.43	0.84

a Each calculated conformation has its mirror counterpart. Optimized at the M06-2X/6-311+G(d,p) method (SCRF, CPCM).

Analysis of the potential energy surface in a weakly polar environment, imitated by chloroform, showed significant changes in the potential energy valley next to conformation C7. Two conformations appear: the helical conformation α (φ, ψ = −62°, −23°) and the semiextended conformation β2 (φ, ψ = −117°, 10°). Both conformations are stabilized by the C^β^-H···O^C^ hydrogen bond. In conformation α, the N-H···Cl hydrogen bond and the dipole attraction type III can be considered. In conformation β2, the value of the torsion angle ψ indicates that the C-terminal amide group and the Cα=Cβ double bond are in a plane; thus, the π-electron conjugation appears.

In a more polar environment, mimicked by water, conformation C7 vanishes. Conformations α and β prevail, both stabilized by the N-H···Cl hydrogen bond. A more polar environment does not have a major influence on the conformation geometry; changes in the values of the torsion angles φ and ψ do not exceed ±3°. The energy order between the conformations is also preserved.

#### Ac-(*E*)-ΔAla(βCl)-NHMe (**2**)

**Figure 3:**
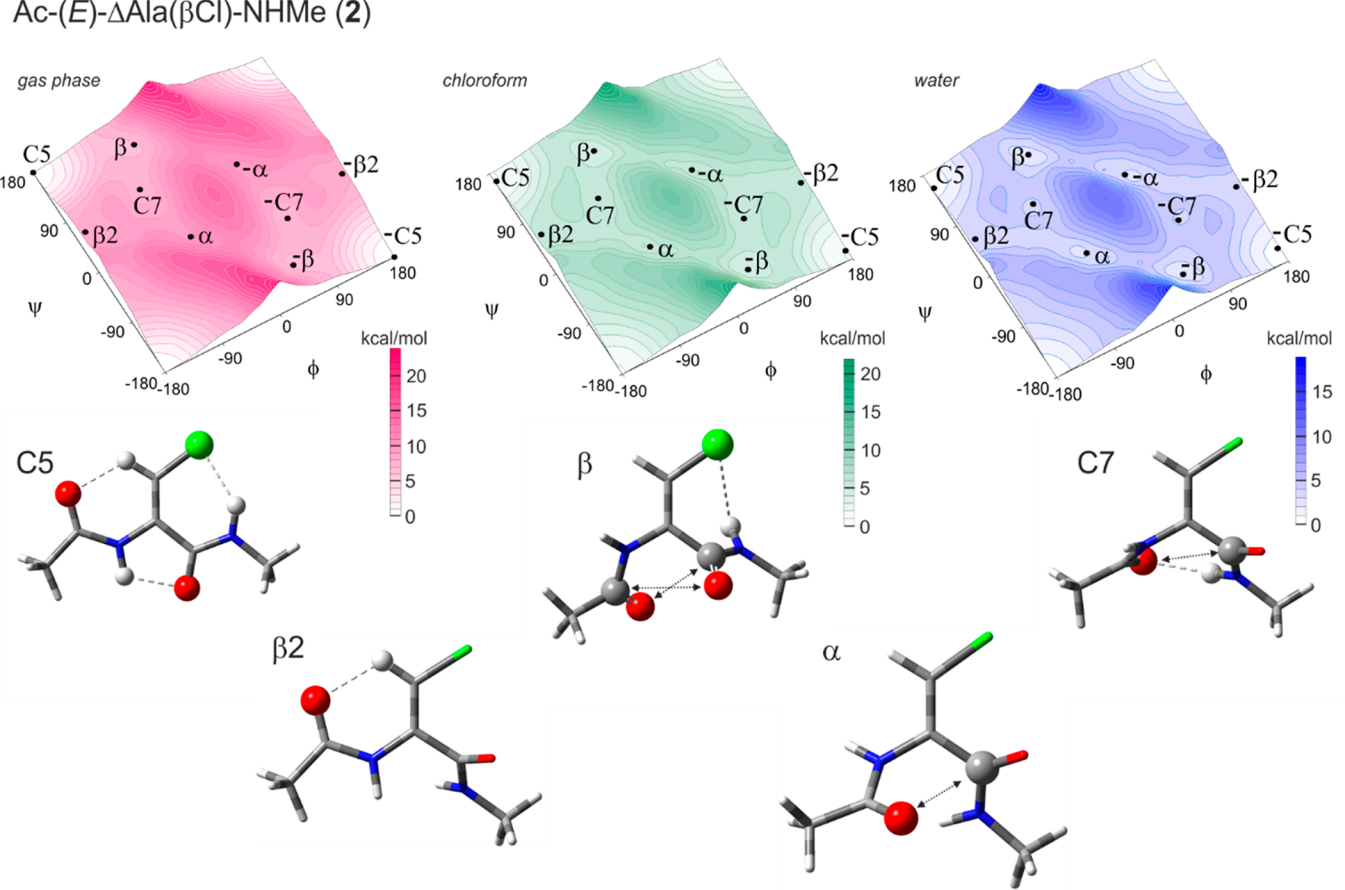
Potential energy surfaces *E* = (φ, ψ) of Ac-(*E*)-ΔAla(βCl)-NHMe (**2**) obtained at the M06-2X/6-31+G(d,p) method in the gas phase, chloroform, and water environment. Energy contours are plotted every 1 kcal/mol. Below the maps are the conformations optimized at the M06-2X/6-311+G(d,p) level of theory in chloroform with the most important electrostatic interactions (↔)^38^ and hydrogen bonds (···)^41^ created within the residue.

The potential energy maps of the diamide model compound (**2**) containing the isomer *E* of the β-chlorodehydroalanine residue together with the conformations corresponding to local minima are presented in Figure 3 and Table 2. Five different conformations can be found, regardless of the environment simulated: C5, β, C7, β2, and α (and their mirror analogues).

**Table 2:** Selected Parameters for Conformations of Ac-(*E*)-ΔAla(βCl)-NHMe (2)^a^

Conformation	φ (deg)	ψ (deg)	Δ*G* (kcal·mol^–1^)	*p* (%)
*gas phase*				
C5	–179.8	180.0	0.00	99.97
β	–46.9	135.6	5.25	0.01
C7	–75.8	69.7	5.45	0.01
β2	–174.0	48.0	6.99	0.00
α	–55.4	–33.4	8.46	0.00
*chloroform*				
C5	–177.9	165.1	0.00	91.87
β	–44.4	132.3	1.77	4.74
C7	–77.7	63.1	2.43	1.56
α	–54.5	–37.6	2.68	1.02
β2	–172.7	53.1	2.81	0.82
*water*				
C5	–177.2	156.2	0.00	60.17
α	–52.6	–39.6	0.71	18.21
β	–44.0	132.2	0.73	17.57
β2	–172.4	52.9	1.82	2.81
C7	–78.6	59.8	2.32	1.23

a Each calculated conformation has its mirror counterpart. Optimized at the M06-2X/6-311+G(d,p) method (SCRF, CPCM).

The global minimum is occupied by the extended conformation C5 (φ, ψ = −180°, 180°). Three intramolecular hydrogen bonds play a stabilizing role: N^N^-H···O^C^, N^C^-H···Cl, and C^β^-H···O^N^. Their parameters can be found in Table 3S in Supporting Information. Additionally, the values of torsion angles φ and ψ indicate the flatness of the structure and, thus, the appearance of the cross-conjugate π-electron system, which overlaps the α,β-double bond and both flanking amide groups. The remaining conformations have clearly much higher energies, which exceed 5 kcal/mol. The existence of conformation β (φ, ψ = −47°, 136°) can be explained by the dipole–dipole attraction type II between the carbonyl groups, like also the N-H···Cl hydrogen contact with considerably lower geometrical parameters. The conformation C7 (φ, ψ = −76°, 70°) is maintained by the N-H···O hydrogen bond C7 type, formed between the C-terminal N-H group and the N-terminal carbonyl group oxygen atom, as well as by the dipole interaction type I. Conformation β2 (φ, ψ = −174°, 48°) is placed in the shallow region of the map. Its main stabilization is provided by the C^β^-H···O^N^ hydrogen bond, whose acceptor is the oxygen atom of the N-terminal amide group. Moreover, π-electron conjugation, including the α,β-double bond and the N-terminal amide group, can be considered. Conformation α (φ,ψ = −55°, −33°) is stabilized by the dipole attraction O^N^···C^C^.

The simulated increase in the polarity of the environment shows that still the lowest energy presents conformation C5. Although the difference in energy between the conformations decreases, up to 3 kcal/mol in chloroform and less than 2.3 kcal/mol in water, most molecules (92%) occur as C5 in a weakly polar solvent, mimicked by chloroform, and about 60% occur as C5 in a natural aquatic environment.

Values of the torsion angle φ are almost unchanged in particular conformations. They are up to ±5° different for the isolated molecule than for the studied solvents. The more significant changes are in the values of the torsion angle ψ. This may be caused by the large chlorine atom in position E of the dehydroamino acid side chain.

Opposite to the gas phase, in the environment of simulated solvents, some molecules appear in conformation β and then also in conformation α. Furthermore, sterically more open conformation α changed its position in energy order. It becomes privileged because of better interaction with the solvent.

### Crystal Structure Analysis

**Figure 4:**
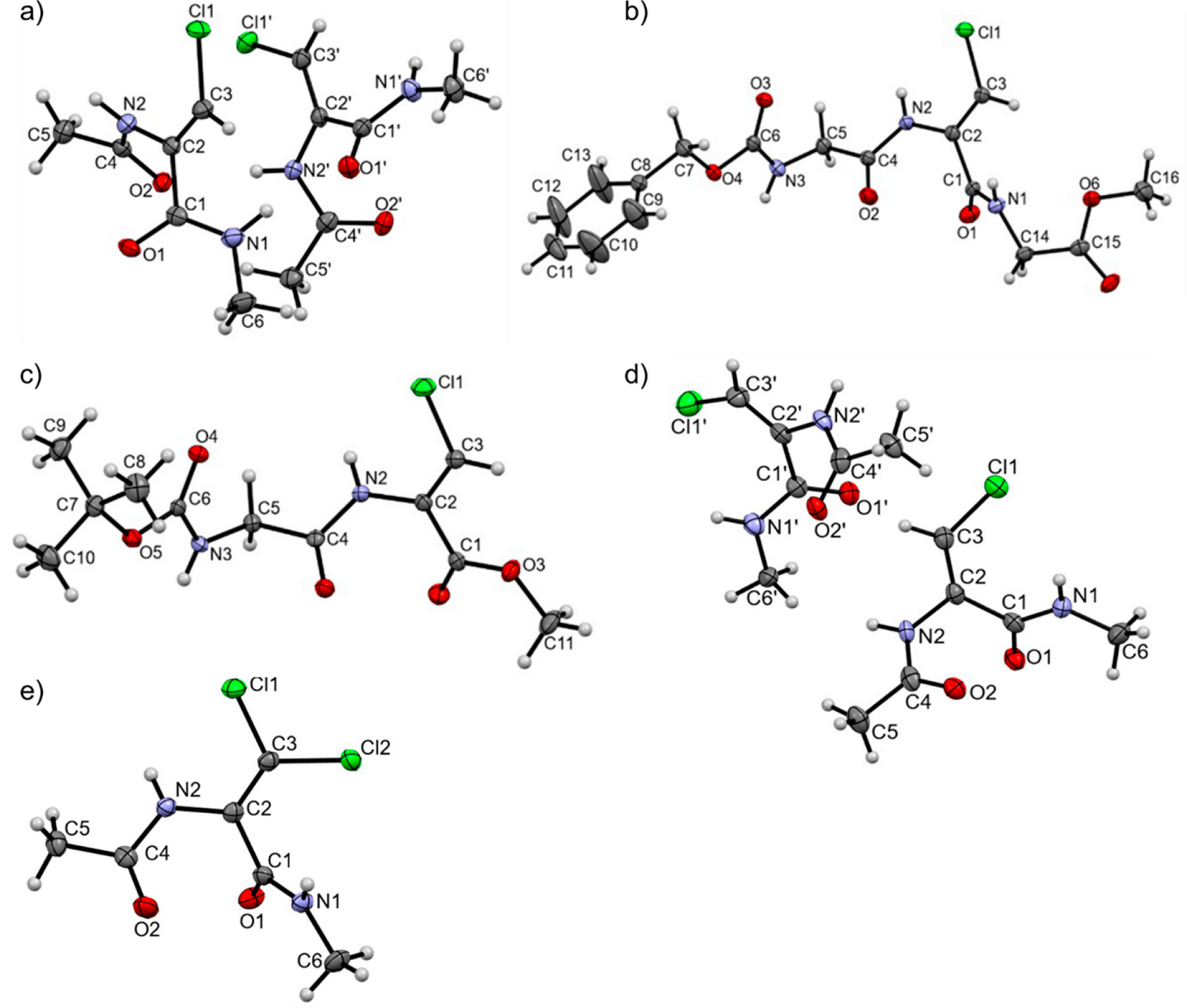
Molecular structures of: a) Ac-(Z)-ΔAla(βCl)-NHMe (**1**), b) Cbz-Gly-(Z)-ΔAla(βCl)-Gly-OMe (**3**), c) Boc-(Z)-ΔAla(βCl)-OMe (**4**), d) Ac-(*E*)-ΔAla(βCl)-NHMe (**2**), and e) Ac-ΔAla(βCl_2_)-NHMe (**5**) in the asymmetric part of the unit cell. Displacement ellipsoids are drawn at the 50% probability level.

The conformational properties of the ΔAla(βCl) residue in a polar solution were estimated using the single X-ray structures of Ac-(Z)-ΔAla(βCl)-NHMe (**1**), Ac-(*E*)-ΔAla(βCl)-NHMe (**2**), Cbz-Gly-(Z)-ΔAla(βCl)-Gly-OMe (**3**), Boc-(Z)-ΔAla(βCl)-OMe (**4**), and Ac-ΔAla(βCl_2_)-NHMe (**5**) determined at 100 K. The crystal parameters and experimental details of X-ray data collection are presented in Tables 4S and 5S. The molecular structures of (**1**–**5**) with the atomic numbering scheme are presented in Figure 4, and selected geometric parameters are presented in Table 3.

**Table 3:** Selected Parameters for the Studied Compounds Measured by the X-ray Method

	Torsion (deg)		Distance (Å)		Angle (deg)	Distance (Å)	
Compound	φ	ψ	H···Cl	N···Cl	N–H···Cl	C^N^···O^C^	C^C^···O^N^
Ac-(Z)-ΔAla(βCl)-NHMe (**1**)	–47.49	149.00	3.009	3.058	84.47	3.046	2.728
	–41.57	141.75	2.868	3.048	94.96	3.116	2.742
Cbz-Gly-(Z)-ΔAla(βCl)-Gly-OMe (**3**)	–41.81	143.49	2.827	3.049	96.30	3.115	2.794
Boc-(Z)-ΔAla(βCl)-OMe (**4**)	–56.51	168.64	2.895	3.057	92.93	3.075	2.979
Ac-(*E*)-ΔAla(βCl)-NHMe (**2**)	–49.53	134.13	2.854	3.221	106.79	3.203	2.807
	–50.04	134.30	2.918	3.256	104.80	3.220	2.833
Ac-ΔAla(βCl_2_)-NHMe (**5**)	–38.86	129.81	2.780	2.975	94.96	3.215	2.731
			2.920	3.114	96.35		

In the crystal structure of Ac-(Z)-ΔAla(βCl)-NHMe (**1**), there are two types of molecules with slightly different geometries. The conformations they adopt (φ, ψ = −47.5°, 149.0° and −41.6°, 141.75°) correspond to conformation β, one of the two most stable forms found on the conformational maps. Analysis of the crystal structure of the larger molecule, tripeptide Cbz-Gly-(Z)-ΔAla(βCl)-Gly-OMe (**3**), shows that the (Z)-ΔAla(βCl) residue also adopts the same conformation β (φ, ψ = −41.8°, 143.5°). Conformation β (φ, ψ = −56.5°, 168.6°) can also be found for Boc-(Z)-ΔAla(βCl)-OMe (**4**) with the C-terminal ester bond, although the values of torsion angles (φ, ψ = −56.5°, 168.6°) are somehow greater than those for the residue with a C-terminal amide group. For the geometrical isomer *E*, as it is shown by Ac-(*E*)-ΔAla(βCl)-NHMe (**2**) that this tendency is maintained, two types of molecules with slightly different geometries were found in the crystal state and they have conformation β (φ, ψ = −49.5°, 134.1° and −50.0°, 134.3°). Finally, Ac-ΔAla(βCl_2_)-NHMe (**5**), with the chlorine atoms in both positions Z and E, also reveals conformation β (φ, ψ = −38.9°, 129.8°).

In conformation β, the hydrogen atom of the N-terminal amide group is in proximity to the chlorine atom. In the studied crystal structures, the H···Cl distance varies from 2.780 to 3.009 Å and the N-H···Cl angle has a value from 96.30 to 84.47° for the (*Z*)-ΔAla(βCl) residue. In the case of the isomer (*E*)-ΔAla(βCl), there is also proximity of the hydrogen atom from the C-terminal amide group and the chlorine atom in position E. The H···Cl distance varies from 2.854 to 2.920 Å, and the N-H···Cl angle has a value from 106.79 to 94.96°. Assuming the sum of van der Waals radii of the hydrogen and chlorine atoms is 2.95 Å,^42^ it can be concluded that there is a N-H···Cl hydrogen bond, which stabilizes conformation β.

Dipole interactions of carbonyl groups II type also can be seen.^38^ Especially, the distance between the C-terminal carbonyl carbon and the N-terminal carbonyl oxygen (C^N^···O^C^) from 2.728 to 2.979 Å, and thus below the sum of van der Waals radii of the carbon and oxygen atoms, also can be perceived as a stabilizing force of conformation β.

**Figure 5:**
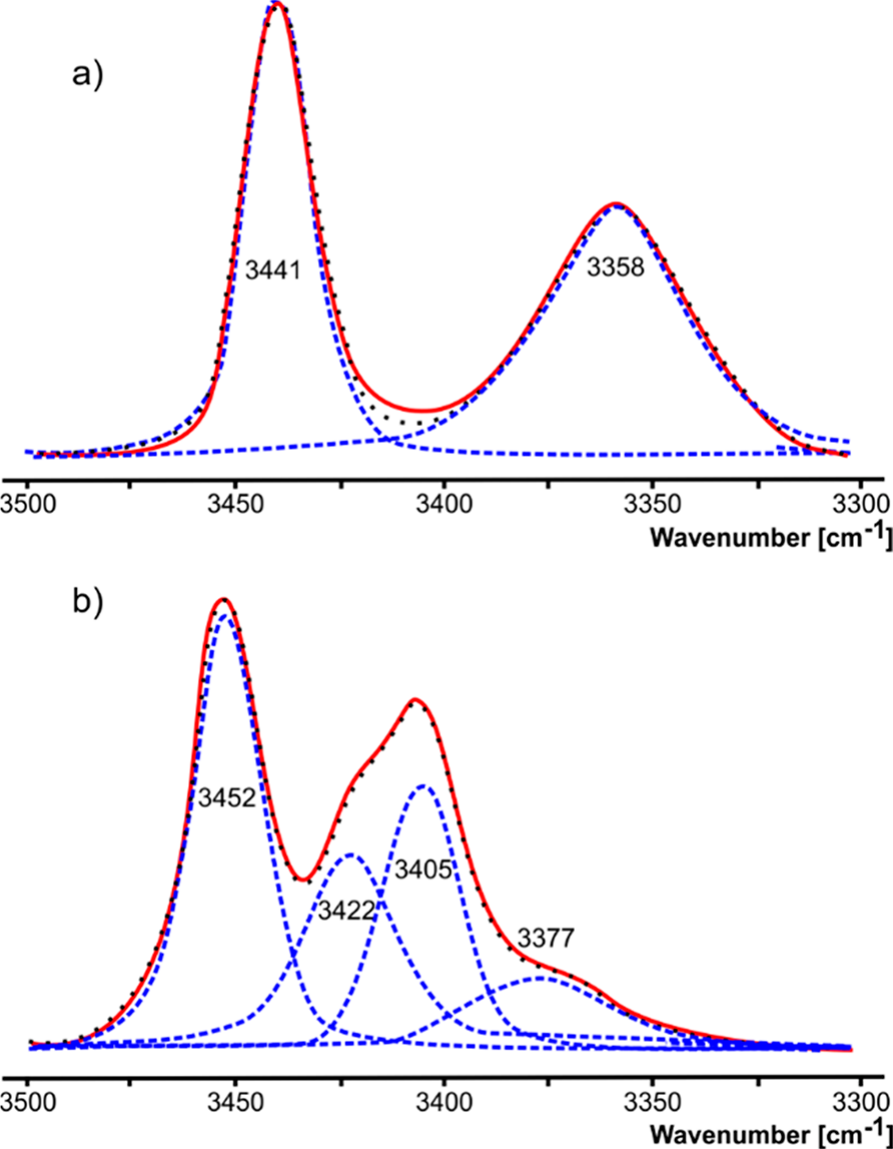
FTIR spectra for Ac-(*Z*/*E*)-ΔAla-(βCl)-NHMe in CHCl_3_, region ν_s_(N-H): (a) isomer *E* (**2**), (b) isomer *Z* (**1**). The component bands (dashed lines) were obtained by a curve-fitting procedure.

The molecules of the studied compounds are mainly associated through N-H···O hydrogen bonds (Figures 1–5S, Table 6S). For Ac-(Z)-ΔAla(βCl)-NHMe (**1**) molecules two similar conformations, here named as β^1^ (φ, ψ = −47.5°, 149.0°) and β^2^ (φ, ψ = −41.6°, 141.75°), are present. The molecule with conformation β^1^ (φ, ψ = −47.5°, 149.0°) creates two N-H···O hydrogen bonds with the molecule with conformation β^2^ (φ, ψ = −41.6°, 141.75°). Additionally, each molecule β1 creates with two molecules −β1 two other N-H···O hydrogen bonds. Analogously, the molecule β^2^ creates two N-H···O hydrogen bonds with two molecules −β^2^ (Figure 1S). For Cbz-Gly-(Z)-ΔAla(βCl)-Gly-OMe (**3**), the molecules are placed in parallel, N-terminus to N-terminus, separately for conformations β and −β (Figure 2S). For Boc-(Z)-ΔAla(βCl)-OMe (**4**), the molecules with conformation β are placed antiparallel between the molecules with conformation −β (Figure 3S). For Ac-(*E*)-ΔAla(βCl)-NHMe (**2**), the molecules are in a linear arrangement. The molecules with conformation β are placed with the molecules with conformation −β alternately, with the (Z)-ΔAla(βCl) side chains upside-down and the N-terminus to the C-terminus (Figure 4S). For Ac-ΔAla(βCl_2_)-NHMe (**5**), the molecules with the opposite conformations, β and −β, and with the ΔAla(βCl_2_) side chains upside-down, create centrosymmetric dimmers. Additionally, each molecule is joined with two other molecules with the opposite conformations (Figure 5S).

Despite various arrangements, the molecules maintain conformation β (or the opposite conformation −β), predicted by the theoretical method as one of the low-energy conformations.

### Infrared Spectral Analysis

**Figure 6:**
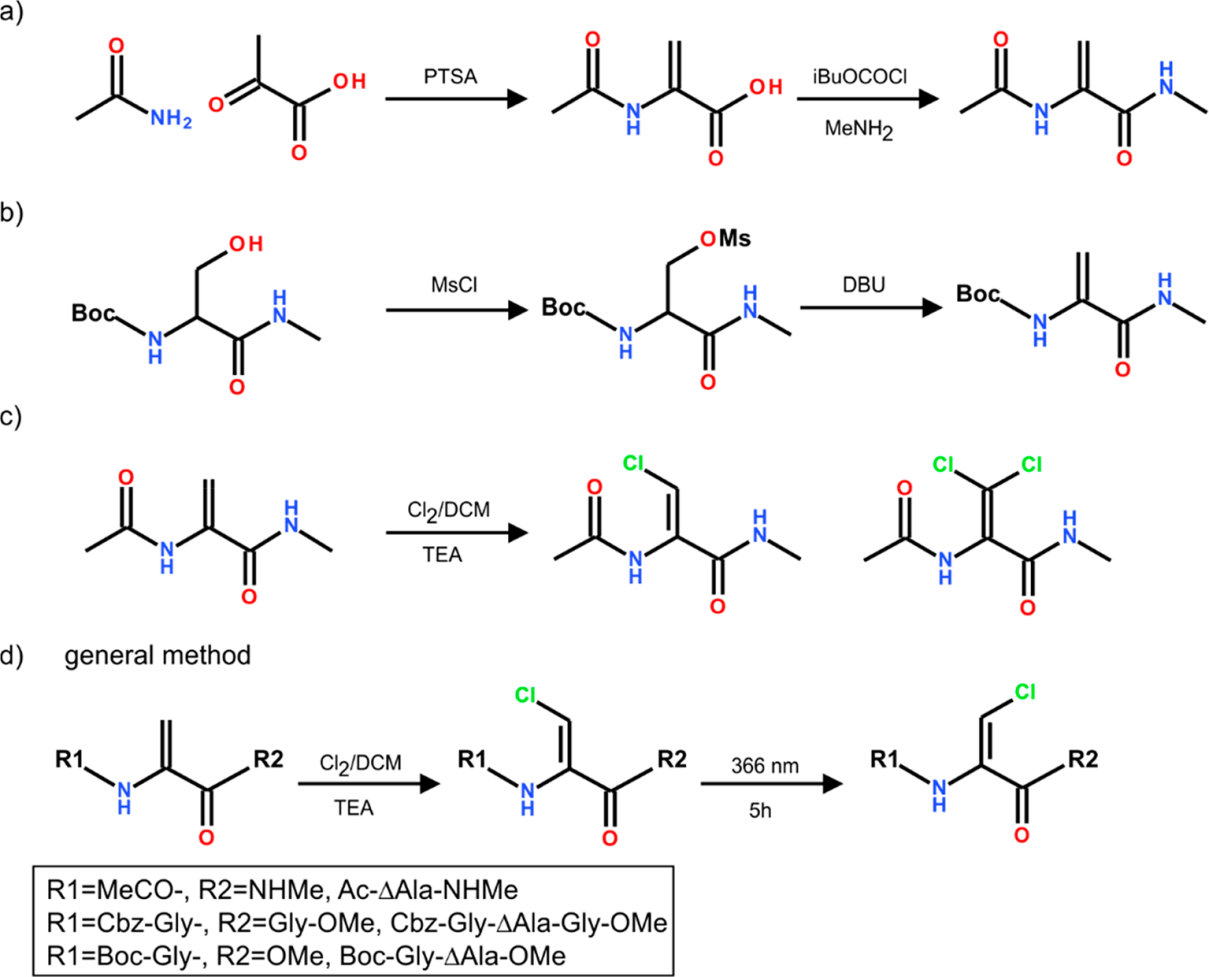
Synthetic procedure of the ΔAla(βCl) residue.

**Figure 7:**
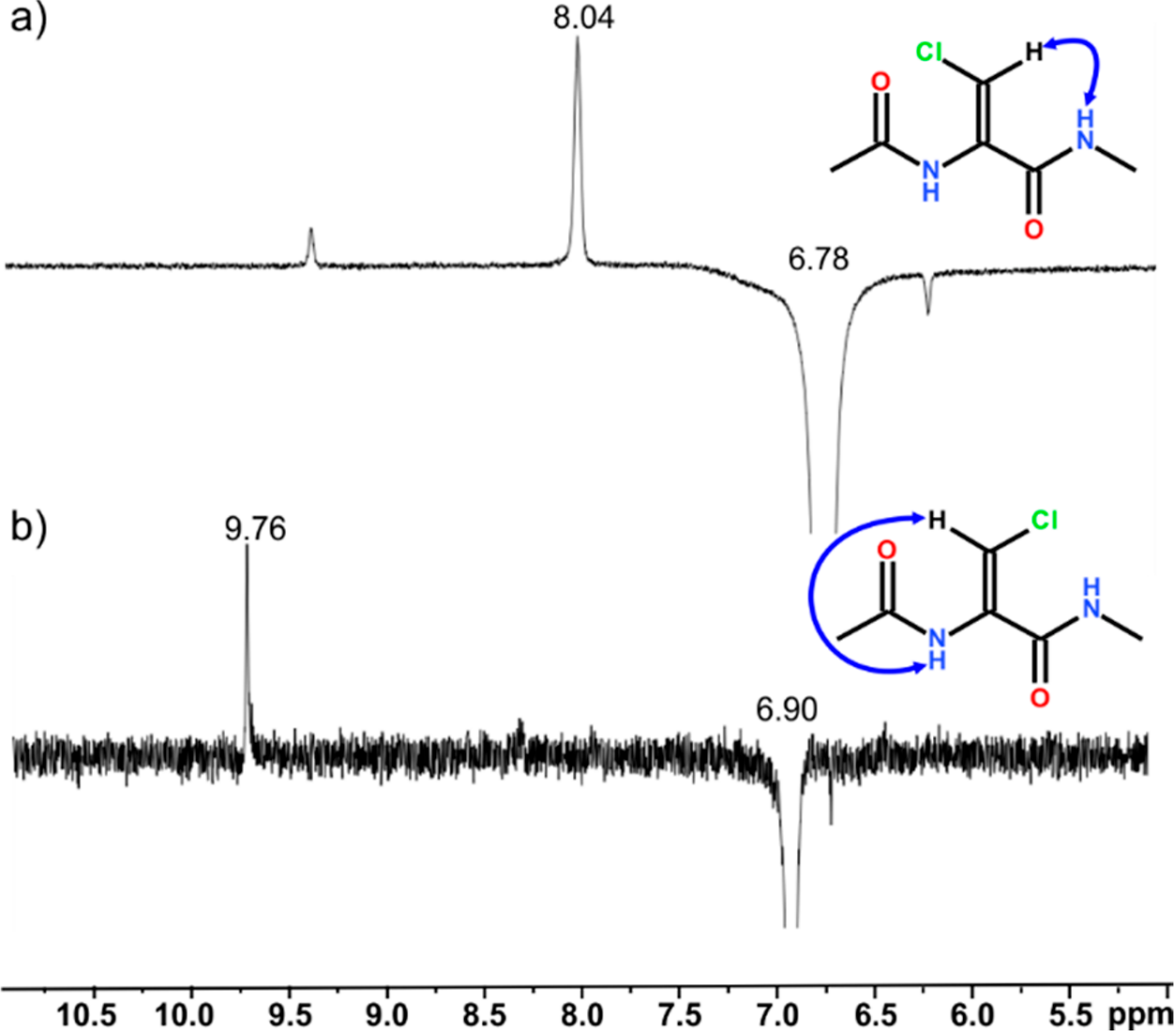
NOE difference NMR spectra of Ac-ΔAla(βCl)-NHMe in DMSO-*d*_6_ with excitation of the vinyl proton of both isomers: (a) isomer *Z* (**1**); (b) isomer *E* (**2**).

The conformational properties of the ΔAla(βCl) residue in a weakly polar solution were estimated using the model compounds Ac-ΔAla(βCl)-NHMe (**1** and **2**) (Figure 6S and 7S).

The ν_s_(N-H) stretching mode regions of the Fourier transform infrared (FTIR) spectra for the solutions in chloroform are shown in Figure 5. The spectrum for Ac-(*E*)-ΔAla(βCl)-NHMe (**2**) shows two bands in the ν_s_(N-H) stretching mode region, at 3441 and 3358 cm^–1^. The analysis of theoretical frequencies (Table 7S) shows that they can be assigned respectively to the C-terminal and N-terminal amide N-H groups. The shapes of the bands are regular, which indicates that they belong to a single conformation. The relative position of the scaled frequencies fits the best to conformation C5, which is also in accordance with the population of the conformation in chloroform presented in Table 2.

The spectrum for Ac-(*Z*)-ΔAla(βCl)-NHMe (**1**) also shows two bands in the ν_s_(N-H) stretching mode region. Nevertheless, the irregularity of their shapes indicates a conformational equilibrium. Deconvolution was resolved on four bands: 3452 and 3405 cm^–1^ with a higher intensity as well as 3422 and 3377 cm^–1^ with a lower intensity. The relative position of the scaled frequencies (Table 7S) allows assuming that in a weakly polar environment conformation C7 can be excluded. Considering the differences in energy presented in Table 1, a mixture of the two conformations α and β is most likely.

It should be noted that the positions of the analyzed bands in the ν_s_(N-H) stretching region for the studied Ac-(*Z*)-ΔAla(βCl)-NHMe and Ac-(*E*)-ΔAla(βCl)-NHMe are considerably lower than the corresponding bands of the structurally closely related Ac-(*Z*)-ΔAbu-NHMe^43^ and Ac-(*E*)-ΔAbu-NHMe,^44^ which have the methyl group in the side chain instead of the chlorine atom. This shows the impact of the chlorine atom in the side chain. Using the lone pairs, it can not only act as the acceptor of the internal N-H···Cl hydrogen bonds with the N-H donors of the flanking amide groups but also participate in a delocalized cross-conjugated system.

### Synthesis

The synthesis of the model compounds containing the β-chlorodehydroalanine residue, ΔAla(βCl), the unsaturated fragment of victorins, was performed in three crucial steps: preparation of the dehydroalanine residue, chlorination of the carbon atom β, and photoisomerization (Figure 6).

The following methods were selected to obtain the dehydroamino acid residue. For the synthesis of the model compounds **1** and **2**, the *N*-acetyldehydroalanine was obtained by condensation of pyruvic acid with acetamide in the presence of *p*-toluenesulfonic acid. In the next step, Ac-ΔAla-OH was converted into methylamide derivative Ac-ΔAla-NHMe using a mixed anhydride method with isobutyl chloroformate (Figure 6a), according to the known procedure.^45^

For the purpose of the synthesis of the model compounds **3** and **4**, dipeptide Boc-Gly-ΔAla-OMe and tripeptide Cbz-Gly-ΔAla-Gly-OMe were obtained using a multistep procedure including preparation of an *N*-protected Gly-ΔAla fragment^46^ followed by its esterification reaction with a cesium salt and methyl iodide^47^ or coupling with a glycine methyl ester using a mixed anhydride protocol,^45,48^ respectively.

An attempt was also undertaken to synthesize the model compound of Boc-protected dehydroalanine, Boc-ΔAla-NHMe, to overcome the solubility problem of short acetyl derivatives in weakly polar solvents, as was done in our previous work.^49^ Nevertheless, the acidic conditions of the condensation reaction are not compatible with the commonly applied amine Boc protection. Therefore, the preparation of Boc-ΔAla-NHMe required another synthetic approach involving dehydration of a β-hydroxy amino acid (serine). The Boc-Ser-NHMe was prepared (please, see Supporting Information). Dehydration using a one pot procedure with methanesulfonyl chloride/DBU was performed,^48^ but a complex reaction mixture was obtained. Thus, the preparation of a methanesulfonyl serine derivative and an elimination reaction were conducted in separate steps (Figure 6b). The analysis of reaction mixtures revealed that the hydantoin derivative was the main product while the desired Boc-ΔAla-NHMe was obtained with a low yield (9%). Taking into account this obstacle, the preparation of the Boc-protected model was abandoned.

The chlorination reaction step was performed on the basis of a modified procedure^31−33^ and our experiences with the bromination of dehydroalanine derivatives.^50^ The reaction was done by treating Ac-ΔAla-NHMe with a solution of chlorine in dichloromethane and then the addition of triethylamine. The isomer *Z* of Ac-ΔAla(βCl)-NHMe (**1**) was obtained as the main product of the reaction. Contrary to the previously mentioned literature protocol, in which the second step of the reaction was performed in acetonitrile in the presence DABCO as organic base, the formation of the isomer *E* of the desired product was not observed in our case. Instead, the minor unsaturated product was a dehydroalanine derivative containing two chlorine atoms at position β, Ac-ΔAla(βCl_2_)-NHMe (**5**) (Figure 6c). On the other hand, the *N*-bromosuccinimide was successfully applied to the synthesis of the β-bromodehydroalanine derivative.^51^ Thus, we decided to perform the chlorination with *N*-chlorosuccinimide as an alternative method, but the desired product was detected in a trace amount. Therefore, the chlorination reaction involving the Cl_2_/DCM solution then triethylamine was chosen as the optimal one. The versatility of this method was shown for the synthesis of more complex structures, tripeptide (Cbz-Gly-ΔAla-Gly-OMe) and dipeptide (Boc-Gly-ΔAla-OMe) substrates, with different positions of the dehydroalanine residue in the peptide chain and different terminations of the C-end. In each case, the isomer *Z*, respectively (**3**) and (**4**), was obtained with a moderate yield of 53–71%.

To complete the preparation of β-chlorodehydroalanine model structures, the isomerization from the isomers *Z* into *E* assisted by UV irradiation was performed. The photoisomerization *Z*/*E* of simple dehydroamino acid residues is known.^52,53^ However, according to the best of our knowledge, the application of this reaction to the β-halogenodehydroalanyl residue is reported for the first time here. The optimal reaction conditions in this case include the irradiation of the reaction mixture with UV light with a maximum wavelength of 366 nm at 5 h with an intensity of 400–440 μW/cm^2^ (Figure 6d). It should be noted that the extension of the reaction time, irradiation with UV light with higher energy (λ_max_ = 254 nm), or increasing the light intensity resulted in our hands in the decomposition of substrate and reduction of overall yield and substrate recovery. Despite several attempts to optimize reaction conditions, the isomerization of Ac-(Z)ΔAla(βCl)-NHMe and Cbz-Gly-(Z)ΔAla(βCl)-Gly-OMe gave the isomer *E* with a yield of 10–15%, while the methyl ester derivative, Boc-Gly-(Z)ΔAla(βCl)-OMe, isomerizes slightly more easily and the product isomer *E* was obtained with a yield of 30%. We speculate that this can be the result of higher steric hindrance of the carboxyamide group. On the other hand, the overall low yields of isomerization *Z*/*E* resulted from higher thermodynamic stability of the isomer *Z*, which is in good accordance with observations for simple dehydroamino acids.^54^

### NMR Spectral Analysis

Apart from the crystallographic structures of both compounds **1** and **2**, the geometries of both isomers of Ac-ΔAla(βCl)-NHMe were confirmed by a series of NOE difference NMR experiments, in which the vinyl and amide protons were excited during the experiment. For the isomer *Z*, a strong resonance is observed between the vinyl (δH 6.78) and C-terminal amide (δH 8.04) protons (Figure 7a). For the isomer *E*, strong resonance is observed between the vinyl (δH 6.90) and, in contrast, the N-terminal amide (δH 9.76) (Figure 7b) protons. The NOE difference NMR spectra for the studied compounds are in Supporting Information (Figures 17-20S, 24-27S, 31-33S, 37-39S).

Determination of the geometric isomer of the chlorodehydroalanine residue in victorin structures was undertaken by comparing the chemical shift of the vinyl proton of the methylated victorin M derivative to the chemical shifts of selected model chemical compounds. Initially, the geometry of the C=C double bond was established as *Z* by the comparison of the vinyl protons of both isomers of *N*-methylated β-chlorodehydroalanine Δ(Me)Ala(βCl).^55,56^ Later, more detailed attempts for the stereochemical assignments of the chlorinated residues in victorin C were undertaken.^10^ The selected ΔAla(βCl) esters were taken as a pattern, and the geometric isomers were assigned by comparing the coupling constant *C*_H_ with the calculated values. Based on this data it was concluded that the chlorodehydroalanyl residue in victorins has *E* rather than *Z* configuration. The geometry *E* of victorins was further adopted in some articles.^6,57,58^

In this work, we synthesized the model compounds, which seem to be structurally better matched to the unsaturated fragment of victorins, because the ΔAla(βCl) is flanked by secondary amide bonds. The structures of the compounds were confirmed by the X-ray method (mainly for the isomer *Z*) and the NOE difference NMR experiments (for both the isomers *Z* and *E*). The comparison of the values of the chemical shift of the vinyl protons for the model compounds (Table 4) revealed that the vinyl proton resonates in the range 6.92–7.15 and 6.58–6.98 ppm, for the isomers *Z* and *E*, respectively (Figures 14–16S, 21–23S, 29S, 30S, 34S, 36S, and 40S). The absolute value of the difference between the positions of the vinyl protons is in the range 0.03–0.39 ppm. The relative position changes with the type of solvent (Ac-ΔAla(βCl)-NHMe) or the type of flanking group (Cbz-Gly-ΔAla(βCl)-Gly-OMe, Boc-Gly-ΔAla(βCl)-OMe). The most significant difference (0.39 ppm in DMSO-*d*_6_) is for the Boc-Gly-ΔAla(βCl)-OMe compound, in which the C-terminus is flanked by a methyl ester. The vinyl proton of the isomer *E* resonates upfield as compared to the isomer *Z* (in DMSO-*d*_6_); however, this is opposite to that established for Ac-ΔAla(βCl)-OMe, where the isomer *E* resonates downfield (in CDCl_3_).^10^ Furthermore, for the derivative of victorin M the vinyl proton of *N*-methylated ΔAla(βCl) resonates at δ = 7.68 ppm in CD_3_OD^55^ and for the product of hydrolysis of victorin C at δ = 7.52 ppm in D_2_O.^59^ Therefore, in our opinion, the geometry of the β-chlorodehydroalanine residue in victorins cannot be definitely assigned by a simple comparison of the chemical shifts of the vinyl protons. Summing up, the data obtained within this study clearly show that the geometrical configuration *Z*/*E* of the β-chlorodehydroalanyl moiety in victorins still remains an open question and the structure should be revised in this particular fragment.

**Table 4:** Chemical Shift (δ ppm) Values of Vinyl Proton for the Studied Compounds

	δ [(ppm)		
Compound/Solvent	Isomer *Z*	Isomer *E*	Δδ (ppm) (*E* – *Z*)
Ac-ΔAla(βCl)-NHMe			
DMSO-*d*_6_	6.78	6.90	0.12
CD_3_OD	6.85	6.81	–0.04
D_2_O	6.92	6.58	–0.34
Cbz-Gly-ΔAla(βCl)-Gly-OMe			
DMSO-*d*_6_	6.95	6.98	0.03
CD_3_OD	7.06	-	-
Boc-Gly-ΔAla(βCl)-OMe			
DMSO-*d*_6_	7.15	6.76	–0.39
Ac-ΔAla(βCl)-OMe^10^			
CDCl_3_	6.92	7.69	0.77

In summary, the conformational preferences of the β-chlorodehydroalanine residue, ΔAla(βCl), the structural constituent of the family of fungal toxins victorins, were determined. Theoretical analysis, using Ramachandran diagrams (*E* = *f*(φ,ψ)) of short diamide model compounds, Ac-(*Z*)-ΔAla(βCl)-NHMe and Ac-(*E*)-ΔAla(βCl)-NHMe, in various environments (gas phase, chloroform, water), gives the overall view of possible conformations. The geometrical isomers differ in their conformational profile. The isomer *Z* prefers the helical conformation α (φ, ψ = −61°, −24°), PPII type conformation β (φ, ψ = −47°, 136°), and semiextended conformation β2 (φ, ψ = −116°, 9°). The isomer *E* prefers mainly the extended conformation C5 (φ, ψ = −177°, 160°) but with an increase of the environment polarity also conformations β (φ, ψ = −44°, 132°) and α (φ, ψ = −53°, −39°). The experimental data and FTIR spectra of Ac-(*Z*)-ΔAla(βCl)-NHMe and Ac-(*E*)-ΔAla(βCl)-NHMe recorded in chloroform confirm the tendency of the isomer *E* toward conformation C5 and the isomer *Z* to the mixture of conformations where α, β, and β2 are the most probable. The X-ray single crystal analyses of the following model compounds, Ac-(*Z*)-ΔAla(βCl)-NHMe, Cbz-Gly-(*Z*)-ΔAla(βCl)-Gly-OMe, Boc-Gly-(*Z*)-ΔAla(βCl)-OMe, Ac-(*E*)-ΔAla(βCl)-NHMe, and Ac-ΔAla(βCl_2_)-NHMe, were performed, and in each case, conformation β was found. The N-H···Cl hydrogen bond created between the chlorine atom in the side chain of the ΔAla(βCl) residue and the N-H group of the flanking amide bonds is present in conformation β, and it seems to influence its relatively low energy.

The method of synthesis of the ΔAla(βCl) residue is proposed. The dehydroalanine substrate undergoes chlorination in dichloromethane in the presence of trimethylamine. The major product is the isomer *Z*. The isomer *E* can be obtained in a photoisomerization reaction. The UV wavelength (λ_max_ = 366 nm), time (5 h), and intensity (400–440 μW/cm^2^) turn out to be important.

The geometry of the the obtained isomers *Z* and *E* was determined by NMR NOE experiments supported by the X-ray method. It was found that the assignment of the geometrical isomer based on the NMR shift of the vinyl proton is not sufficiently precise to determine the geometry isomers, despite the remarkable effort described in the literature. Therefore, we suggest that the geometrical isomer of the ΔAla(βCl) residue in naturally occurring victorins still remains an open question and should be revised.

The geometrical isomer of the dehydroamino acid residue is crucial for biological activity. In the mentioned example of phytotoxic phomalide, produced by the fungus *Leptosphaeria maculans* and responsible for leaf spot and stem cancer (blackleg), a disease of oilseed Brassicas (e.g., canola), the isomer *E* of the dehydrobutyrine residue with a C-terminal ester is present.^60^ In contrast, the isophomalide with the isomer *Z* is biologically inactive.^10^ In another example, tentoxin, a selective weed killer that causes chlorosis of higher plants, the isomer *Z* of *N*-methyldehydrophenylalanine is present. In contrast, isotentoxin with the isomer *E* is biologically inactive.^61^ For both dehydroamino acids, the geometrical isomers have different conformational properties.^62,63^ Therefore, it can be assumed that for victorins, the host-selective toxins from the oat pathogen *Cochliobolus victoriae*, not only the presence of the β-chlorodehydroalanine residue is important but also the proper isomer *Z* or *E*. The present study shows that both the geometrical isomers differ in conformational preferences; therefore, they should have different impacts on the native conformation of victorins and, thus, on the biological activity. The proposed method of synthesis and further photoisomerization enable us to gain deeper insight into the molecular function of victorins, e.g. by comparison of the biological activity of semisynthetic isovictorins. It should be also noticed that the chlorovinyl function is a relatively reactive functional group, where the chlorine atom can be effectively changed by a nucleophile.^32,64,65^ This opens a way to apply the β-chlorodehydroalanine residue in peptide design.

## Supporting Information Available

Theoretical calculations: XYZ structures of the calculated compounds (**1** and **2**), calculated parameters of hydrogen bonds and dipole interactions in conformations **1** and **2**. X-ray crystal parameters and experimental details of the studied compounds **1**–**5**, selected geometric and hydrogen-bond parameters, and molecular interactions for **1**–**5**. FTIR spectra for **1** and **2** in CHCl_3_ and theoretical frequencies for **1** and **2**. Procedure of synthesis. NMR spectra for **1**–**5**. (PDF)
