# Supplementary material for: Insight into the Structure of Victorin, the Host-Selective Toxin from the Oat Pathogen Cochliobolus victoriae. Studies of the Unique Dehydroamino Acid β-Chlorodehydroalanine
Source: J Agric Food Chem. 2023 Jul 24;71(30):11642–53. doi: 10.1021/acs.jafc.3c01387 (PMC10401702; doi:10.1021/acs.jafc.3c01387)
Supplement: Supplementary file 1 — jf3c01387_si_001.pdf [file jf3c01387_si_001.pdf]

Insight into the structure of victorin, the host-selective toxin from the oat pathogen *Cochliobolus victoriae*. Studies of unique dehydroamino acid, β-chlorodehydroalanine

Karolina Banaś[1], Paweł Lenartowicz*[1], Monika Staś[1], Błażej Dziuk[2,3], Dawid Siodłak*[1]
[1]Faculty of Chemistry, University of Opole, Oleska 48, 45-052 Opole, Poland
[2]Faculty of Chemistry, Wroclaw University of Science and Technology, Wybrzeze Wyspianskiego 27, 50-370 Wroclaw, Poland
[3]Faculty of Chemistry, University of Wroclaw, Joliot-Curie 14, Wroclaw 50-383, Poland
Correspondence: plenartowicz@uni.opole.pl, dsiodlak@uni.opole.pl

Table of contents	Page
**Theoretical Calculations**	
**Table 1S.** XYZ structures of the calculated compounds, Ac-(*Z*)-ΔAla(βCl)-NHMe (**1**) and Ac-(*E*)-ΔAla(βCl)-NHMe (**2**), along with their electronic energies.	3
**Table 2S.** Structural parameters for the internal hydrogen bond X–H···A (X = N, C; A = O, Cl) and C=O ◄···► O=C interactions in the M06-2X/6-311+G(d,p) conformers of Ac-(*Z*)-ΔAla(βCl)-NHMe (**1**) in various environment.	14
**Table 3S**. Structural parameters for the internal hydrogen bond X–H···A (X = N, C; A = O, Cl) and C=O ◄ ···► O=C interactions in the M06-2X/6-311+G(d,p) conformers of Ac-(*E*)-ΔAla(βCl)-NHMe (**2**) in various environment.	15
**X-Ray**	
**Table 4S**. Crystal Parameters and Experimental Details of X-Ray Data Collection for the Studied Compounds.	16
**Table 5S**. Selected geometric parameters (Å, °) of the studied compounds as determined by X-ray method.	16
**Figure 1S.** Molecular interactions of the Ac-(Z)-ΔAla(βCl)-NHMe (**1**) molecules.	21
**Figure 2S.** Molecular interactions of the Cbz-Gly-(Z)-ΔAla(βCl)-Gly-OMe (**3**) molecules.	22
**Figure 3S.** Molecular interactions of the Boc-(Z)-ΔAla(βCl)-OMe (**4**) molecules.	23
**Figure 4S.** Molecular interactions of the Ac-(*E*)-ΔAla(βCl)-NHMe (**2**) molecules.	24
**Figure 5S.** Molecular interactions of the Ac-ΔAla(βCl_2)-NHMe (**5**) molecules.	25
**Table 6S**. Selected hydrogen-bond parameters (Å, °) of the studied compounds as determined by X-ray method.	26
**IR**	
**Figure 6S.** FTIR spectra for the Ac-(Z)-ΔAla(βCl)-NHMe (**1**) and Ac-(*E*)-ΔAla(βCl)-NHMe (**2**)in $CHCl_3$.	27
**Figure 7S.** FTIR spectra for the Ac-(*Z*)-ΔAla(βCl)-NHMe (**1**) and Ac-(*E*)-ΔAla(βCl)-NHMe (**2**) in $CHCl_3$, region ν_s(N–H) and ν_s(C=O).	28
**Table 7S.** Theoretical frequencies obtained by M06-2X/6-311+G(d,p) method for the conformations of the Ac-(Z)-ΔAla(βCl)-NHMe (**1**) and Ac-(*E*)-ΔAla(βCl)-NHMe (**2**) in chloroform	28
**Synthesis**	29
**NMR**	
**Figure 8S.** ^{1}H and ^{13}C NMR spectra of Boc-L-Ser-NHMe in chloroform.	33
**Figure 9S.** ^{1}H and ^{13}C NMR spectra of Boc-L-Ser(OMs)-NHMe in DMSO-d_6.	34
**Figure 10S.** ^{1}H and ^{13}C NMR spectra of Boc-ΔAla-NHMe in DMSO-d_6.	35
**Figure 11S.** ^{1}H and ^{13}C NMR spectra of cyclic side product Boc-ΔAla-NHMe synthesis in CD_3Cl.	36
**Figure 12S.** ^{1}H and ^{13}C NMR spectra of Ac-ΔAla-OH in DMSO-d_6.	37
**Figure 13S.** ^{1}H and ^{13}C NMR spectra of Ac-ΔAla-NHMe in DMSO-d_6.	38
**Figure 14S.** ^{1}H and ^{13}C NMR spectra of Ac-(*Z*)-ΔAla(βCl)-NHMe (**1**) in DMSO-d_6.	39
**Figure 15S.** ^{1}H and ^{13}C NMR spectra of Ac-(*Z*)-ΔAla(βCl)-NHMe (**1**) in CD_3OD.	40
**Figure 16S.** ^{1}H and ^{13}C NMR spectra of Ac-(*Z*)-ΔAla(βCl)-NHMe (**1**) in D_2O.	41
**Figure 17S.** ^{1}H NMR 1D-NOE spectra obtained by selective excitation of the N-terminal amide H atom of Ac-(*Z*)-ΔAla(βCl)-NHMe (**1**) in DMSO-d_6.	42
**Figure 18S.** ^{1}H NMR 1D-NOE spectra obtained by selective excitation of the C-terminal amide H atom of Ac-(*Z*)-ΔAla(βCl)-NHMe (**1**) in DMSO-d_6.	42
**Figure 19S.** ^{1}H NMR 1D-NOE spectra obtained by selective excitation of the side chain H atom of Ac-(*Z*)-ΔAla(βCl)-NHMe (**1**) in DMSO-d_6.	43

**Figure 20S.** ^{1}H NMR 1D-NOE spectra obtained by selective excitation of the N-terminal amide H atom, the C-terminal amide H atom and the side chain H atom of Ac-(*Z*)-ΔAla(βCl)-NHMe (**1**) in DMSO-d_6.	43
**Figure 21S.** ^{1}H and ^{13}C NMR spectra of Ac-(*E*)-ΔAla(βCl)-NHMe (**2**) in DMSO-d_6.	44
**Figure 22S.** ^{1}H and ^{13}C NMR spectra of Ac-(*E*)-ΔAla(βCl)-NHMe (**2**) in CD_3OD.	45
**Figure 23S.** ^{1}H and ^{13}C NMR spectra of Ac-(*E*)-ΔAla(βCl)-NHMe (**2**) in D_2O.	46
**Figure 24S.** ^{1}H NMR 1D-NOE spectra obtained by selective excitation of the N-terminal amide H atom of Ac-(*E*)-ΔAla(βCl)-NHMe (**2**) in DMSO-d_6.	47
**Figure 25S.** ^{1}H NMR 1D-NOE spectra obtained by selective excitation of the C-terminal amide H of Ac-(*E*)-ΔAla(βCl)-NHMe (**2**) in DMSO-d_6.	47
**Figure 26S.** ^{1}H NMR 1D-NOE spectra obtained by selective excitation of the side chain H atom of Ac-(*E*)-ΔAla(βCl)-NHMe (**2**) in DMSO-d_6.	48
**Figure 27S.** ^{1}H NMR 1D-NOE spectra obtained by selective excitation of the N-terminal amide H atom, the C-terminal amide H atom and the side chain H atom of Ac-(*E*)-ΔAla(βCl)-NHMe (**2**) in DMSO-d_6.	48
**Figure 28S.** ^{1}H NMR spectrum of Cbz-Gly-ΔAla-Gly-OMe in DMSO-d_6.	49
**Figure 29S.** ^{1}H and ^{13}C NMR spectra of Cbz-Gly-(*Z*)-ΔAla(βCl)-Gly-OMe (**3**) in DMSO-d_6.	50
**Figure 30S.** ^{1}H and ^{13}C NMR spectra of Cbz-Gly-(*Z*)-ΔAla(βCl)-Gly-OMe (**3**) in CD_3OD.	51
**Figure 31S.** ^{1}H NMR 1D-NOE spectra obtained by selective excitation of the N-terminal amide H of Cbz-Gly-(*Z*)-ΔAla(βCl)-Gly-OMe (**3**) in DMSO-d_6.	52
**Figure 32S.** ^{1}H NMR 1D-NOE spectra obtained by selective excitation side chain H atom (blue spectrum) of Cbz-Gly-(*Z*)-ΔAla(βCl)-Gly-OMe (**3**) in DMSO-d_6.	52
**Figure 33S.** ^{1}H NMR 1D-NOE spectra obtained by selective excitation of the N-terminal amide H atom and the side chain H atom of Cbz-Gly-(*Z*)-ΔAla(βCl)-Gly-OMe (**3**) in DMSO-d_6.	53
**Figure 34S.** ^{1}H and ^{13}C NMR spectra of Cbz-Gly-(*E*)-ΔAla(βCl)-Gly-OMe in DMSO-d_6.	54
**Figure 35S.** ^{1}H spectrum of Boc-Gly-ΔAla-OMe in DMSO-d_6.	55
**Figure 36S.** ^{1}H and ^{13}C NMR spectra of Boc-Gly-(*Z*)-ΔAla(βCl)-OMe (**4**) in DMSO-d_6.	56
**Figure 37S.** ^{1}H NMR 1D-NOE spectra obtained by selective excitation of the N-terminal amide H atom of Boc-Gly-(*Z*)-ΔAla(βCl)-OMe (**4**) in DMSO-d_6.	57
**Figure 38S.** ^{1}H NMR 1D-NOE spectra obtained by selective excitation of the side chain H atom of Boc-Gly-(*Z*)-ΔAla(βCl)-OMe (**4**) in DMSO-d_6.	57
**Figure 39S.** ^{1}H NMR 1D-NOE spectra obtained by selective excitation of the N-terminal amide H atom and the side chain H atom of Boc-Gly-(*Z*)-ΔAla(βCl)-OMe (**4**) in DMSO-d_6.	58
**Figure 40S.** ^{1}H and ^{13}C NMR spectra of Boc-Gly-(*E*)-ΔAla(βCl)-OMe in DMSO-d_6.	59
**Figure 41S.** ^{1}H and ^{13}C NMR spectra of Ac-ΔAla(βCl_2)-NHMe (**5**) in DMSO-d_6.	60

Table 1S. XYZ structures of the calculated compounds, Ac-(*Z*)-ΔAla(βCl)-NHMe (**1**) and Ac-(*E*)-ΔAla(βCl)-NHMe (**2**), along with their electronic energies.

Ac-(*Z*)-ΔAla(βCl)-NHMe (**1**)	
	**Gas phase**
conformation C7	20<br>Ac-(Z)-ΔAla(βCl)-NHMe (1) gas phase C7 M062X/6-311+G(d,p) Charge = 0 Multiplicity = 1 Electronic Energy= -954.159514254 Electronic_Energy_with_ZeroPoint_Correction= -954.004264 Gibbs_Free_Energy= -954.044886 LowF= 38.8627 NImag= 0<br>N -0.0806596126 0.3092529903 0.1377354803<br>H -0.1249075957 0.706447957 1.0690642236<br>C 1.1882538513 0.2953964685 -0.5688564768<br>H 1.9928026071 0.2375275063 0.1617713898<br>H 1.2305637526 -0.5732085916 -1.2253188504<br>H 1.3132684957 1.1909014288 -1.1829139702<br>C -1.2211060313 0.2709138662 -0.5794856948<br>O -1.2458514634 0.1634900511 -1.792288385<br>C -2.5278811602 0.3306919596 0.1952642623<br>N -2.6384266034 0.015444664 1.5721199527<br>H -3.4517746608 -0.5380688767 1.8059697986<br>C -2.0335257725 0.6580312177 2.6226405811<br>O -1.1389958936 1.4674974261 2.479178896<br>C -2.5763621383 0.2872343058 3.98219631<br>H -1.7874522006 0.4201325093 4.7189313635<br>H -3.3942010291 0.9698119474 4.2250391802<br>H -2.9521145215 -0.7354792039 4.0177549378<br>C -3.6199888969 0.5364325725 -0.5402311651<br>H -3.5621006337 0.6593032014 -1.6112483591<br>Cl -5.212612271 0.5656538776 0.1256174307
conformation β	20<br>Ac-(Z)-ΔAla(βCl)-NHMe (1) gas phase β M062X/6-311+G(d,p) Charge = 0 Multiplicity = 1 Electronic Energy= -954.15847483 Electronic_Energy_with_ZeroPoint_Correction= -954.003727 Gibbs_Free_Energy= -954.043851 LowF= 53.7575 NImag= 0<br>N 0.0008633743 -0.0684487823 -0.0429468185<br>H 0.0033834265 -0.1711843786 0.9602953434<br>C 1.2701045443 -0.1281806765 -0.7493394431<br>H 1.919345853 -0.8452388718 -0.2501446087<br>H 1.0928003529 -0.4548004503 -1.772897558<br>H 1.752554818 0.8521085558 -0.7785876523<br>C -1.0109856081 0.6629535757 -0.5744822238<br>O -0.9913117676 1.1563575061 -1.6787018956<br>C -2.2521366123 0.7551271903 0.2914731342<br>N -2.8254899689 2.0168427315 0.403203092<br>H -3.8312358353 2.08664448 0.4544404754<br>C -2.0488144245 3.1508938886 0.5560529155<br>O -0.8516780699 3.0784704044 0.7020806186<br>C -2.8087182854 4.4516879704 0.4942908876<br>H -2.2370205468 5.2163010093 1.0146471566<br>H -2.911891619 4.7402132662 -0.5542100911<br>H -3.8055116866 4.368979975 0.9297512254<br>C -2.8105846957 -0.3608819499 0.757218383<br>H -2.4024522294 -1.3424599126 0.5711896826<br>Cl -4.3098833061 -0.3537057784 1.6295794044
conformation C5	20<br>Ac-(Z)-ΔAla(βCl)-NHMe (1) gas phase C5 M062X/6-311+G(d,p) Charge = 0 Multiplicity = 1 Electronic Energy= -954.156786227 Electronic_Energy_with_ZeroPoint_Correction= -954.001694 Gibbs_Free_Energy= -954.041825 LowF= 53.6749 NImag= 0<br>N -0.006052635 -0.1569366385 0.018101592<br>H -0.0681042737 -0.5541338979 0.9415750605<br>C 1.2953265048 -0.0651289038 -0.6236197985<br>H 2.0603852601 0.0239629425 0.1452726249<br>H 1.4978479815 -0.9412437258 -1.2441918899<br>H 1.3198892301 0.8180970551 -1.2603572873<br>C -1.1302564308 -0.1090567536 -0.7359781415<br>O -1.1138382336 0.0434042355 -1.9442117185<br>C -2.4510991112 -0.2231193852 -0.021968874<br>N -3.4801450748 -0.67714921 -0.8531910022<br>H -3.3216253168 -0.4779692285 -1.8338719868<br>C -4.3068151368 -1.7455385227 -0.5344208732<br>O -4.267729957 -2.304950703 0.5332600829<br>C -5.2828050503 -2.1085107847 -1.6295704905

	H -5.6185964917 -3.1304066605 -1.4707548626<br>H -6.1436410489 -1.4392594314 -1.5662998097<br>H -4.8442904577 -2.0086926048 -2.6234918738<br>C -2.6047217749 0.196881294 1.2349387623<br>H -1.7720654051 0.560932793 1.8187218829<br>Cl -4.1144505194 0.3072486811 2.0416184852
conformation C5'	20<br>Ac-(Z)-ΔAla(βCl)-NHMe (1) gas phase C5' M062X/6-311+G(d,p) Charge = 0 Multiplicity = 1 Electronic Energy= -954.155098076 Electronic_Energy_with_ZeroPoint_Correction= -954.000178 Gibbs_Free_Energy= -954.041135 LowF= 28.2457 NImag= 0<br>N -0.0009707544 -0.107518682 -0.0304763801<br>H 0.0041135914 -0.2393191279 0.9668494471<br>C 1.2673852629 -0.062926404 -0.7385456353<br>H 2.00679304 -0.6228718354 -0.1691379114<br>H 1.1488960352 -0.5184716856 -1.7208343447<br>H 1.6112122762 0.9649250248 -0.8772387393<br>C -1.1132038758 0.3940285132 -0.6204699358<br>O -1.1010598339 0.8721859398 -1.7402172189<br>C -2.39845714 0.3688537396 0.1706723271<br>N -3.3853787263 1.1905727258 -0.3734507715<br>H -3.0164051058 1.9018170191 -0.993097559<br>C -4.6573997044 0.7238615755 -0.7122968222<br>O -5.0597464462 -0.3657068764 -0.3982207647<br>C -5.4714577106 1.7313403851 -1.4908739365<br>H -6.4231581046 1.2783818819 -1.7555440743<br>H -5.6431035928 2.6218532705 -0.883353576<br>H -4.9445042354 2.0326997605 -2.3989612613<br>C -2.5425957956 -0.3743051709 1.2718520485<br>H -1.7959435325 -1.0922518908 1.5772749123<br>Cl -3.8905784165 -0.3013733476 2.3310857204
**Chloroform**	
conformation α	20<br>Ac-(Z)-ΔAla(βCl)-NHMe (1) chloroform α M062X/6-311+G(d,p) Charge = 0 Multiplicity = 1 Electronic Energy= -954.171778918 Electronic_Energy_with_ZeroPoint_Correction= -954.017304 Gibbs_Free_Energy= -954.058071 LowF= 41.2221 NImag= 0<br>N -0.0923550601 0.090157349 0.0394705624<br>H -0.1220834647 0.3269652619 1.0185655395<br>C 1.1868263734 -0.0547359718 -0.6318616657<br>H 1.9760429944 0.2374435407 0.0566587059<br>H 1.3418123501 -1.0897413932 -0.942613295<br>H 1.2251706629 0.5823752369 -1.5163365729<br>C -1.2393968596 -0.1874203959 -0.6020081575<br>O -1.2902965764 -0.5017124629 -1.7821475624<br>C -2.4947443941 -0.0210162501 0.2187585799<br>N -2.4138378182 -0.0876129742 1.622092609<br>H -2.9802985205 0.5628271338 2.1516678103<br>C -1.9846310638 -1.2330630086 2.2668148936<br>O -1.5128581312 -2.1665904788 1.6530815549<br>C -2.1558439003 -1.2139423618 3.7636632794<br>H -1.498068952 -1.9591363742 4.2042289288<br>H -1.9426356597 -0.2300778795 4.1817189195<br>H -3.1906218646 -1.4722405843 4.0012107549<br>C -3.6120369272 0.2798630965 -0.4380025967<br>H -3.630424167 0.395892219 -1.5112961189<br>Cl -5.1206400041 0.5432663878 0.3582515401
conformation β	20<br>Ac-(Z)-ΔAla(βCl)-NHMe (1) chloroform β M062X/6-311+G(d,p) Charge = 0 Multiplicity = 1 Electronic Energy= -954.171809467 Electronic_Energy_with_ZeroPoint_Correction= -954.017286 Gibbs_Free_Energy= -954.0578 LowF= 54.3536 NImag= 0<br>N 0.0216916882 -0.0927684112 -0.0347163912<br>H 0.038532259 -0.2143426445 0.9660963079<br>C 1.2673963459 -0.1527297134 -0.7808025849<br>H 2.0040170511 -0.6881930151 -0.1863551615<br>H 1.1081305596 -0.6825990087 -1.7195772519<br>H 1.6386395172 0.849826607 -1.0046502159<br>C -1.0883004638 0.4167152161 -0.5993010401<br>O -1.1536096828 0.797162518 -1.7550224377<br>C -2.31820761 0.4186733013 0.2830617538<br>N -3.1171605811 1.558516797 0.2331493162

	H -4.119843066 1.4465467324 0.2925013586<br>C -2.5832331599 2.8274086861 0.2197848903<br>O -1.3879056335 3.0116507343 0.3284872328<br>C -3.5824793916 3.9378323311 0.0318843889<br>H -3.1959944017 4.8431133078 0.4943428805<br>H -3.7007763652 4.1144109626 -1.039702068<br>H -4.5580682905 3.6911155325 0.4501779518<br>C -2.6620475975 -0.6981663072 0.9237307887<br>H -2.0884020794 -1.6108589142 0.8679710173<br>Cl -4.1224522359 -0.8229766272 1.8480362516
conformation β2	20<br>Ac-(Z)-ΔAla(βCl)-NHMe (1) chloroform β2 M062X/6-311+G(d,p) Charge = 0 Multiplicity = 1 Electronic Energy= -954.170489823 Electronic_Energy_with_ZeroPoint_Correction= -954.016167 Gibbs_Free_Energy= -954.057182 LowF= 39.369 NImag= 0<br>N -0.0276258324 -0.0579360581 0.0271819674<br>H -0.0451014017 -0.1290639625 1.0325858511<br>C 1.2429181905 -0.0437207039 -0.6759092633<br>H 2.0388470248 0.1260248615 0.0453475017<br>H 1.4154320242 -0.9912113565 -1.1905146596<br>H 1.2478742308 0.7572521311 -1.415453946<br>C -1.1874527074 -0.1341602565 -0.6509753002<br>O -1.2470911314 -0.15835616 -1.8725735891<br>C -2.4265018517 -0.1852650586 0.2041282376<br>N -2.3148370011 0.0578009938 1.5915901022<br>H -2.1248134915 1.0037714628 1.8942462158<br>C -2.6094930298 -0.8880732756 2.5503022938<br>O -2.8618609677 -2.0393791016 2.2650866392<br>C -2.6079615001 -0.36858301 3.9659741285<br>H -2.3563423558 -1.1836713781 4.6407385099<br>H -1.9136396076 0.4599465816 4.1049723909<br>H -3.6161401289 -0.0193336002 4.2018112375<br>C -3.57078106 -0.5022671318 -0.3973166724<br>H -3.6044633281 -0.7291518689 -1.4533095033<br>Cl -5.0801668296 -0.5807950983 0.4167699612
conformation C7	20<br>Ac-(Z)-ΔAla(βCl)-NHMe (1) chloroform C7 M062X/6-311+G(d,p) Charge = 0 Multiplicity = 1 Electronic Energy= -954.170082292 Electronic_Energy_with_ZeroPoint_Correction= -954.015346 Gibbs_Free_Energy= -954.056713 LowF= 19.5464 NImag= 0<br>N -0.026832007 0.1592312071 -0.0828054867<br>H -0.0160541188 0.314777635 0.9163443455<br>C 1.2403922663 0.0787390408 -0.7902376121<br>H 1.9804977865 0.6566596756 -0.241387941<br>H 1.5801665558 -0.9557494747 -0.8811964903<br>H 1.1284708596 0.4942087798 -1.7912094861<br>C -1.1275935464 -0.3909773335 -0.6236521348<br>O -1.1368453301 -0.9184836344 -1.7279067751<br>C -2.4048929807 -0.2991662534 0.1852070985<br>N -2.5559214828 0.6053493035 1.2656143429<br>H -3.3865421598 1.1821797891 1.238530399<br>C -1.8939373488 0.529495697 2.4600629032<br>O -0.9537887905 -0.2292224871 2.6317998053<br>C -2.4063938333 1.442568045 3.542901846<br>H -1.5580566357 1.8294790811 4.104557726<br>H -3.0031746408 2.2665429067 3.1548557843<br>H -3.0224665336 0.8485301248 4.2214469562<br>C -3.4349402579 -1.0189852835 -0.2575401017<br>H -3.3580309854 -1.6363821793 -1.1400301928<br>Cl -4.9827468167 -1.0081407617 0.498394104
conformation C5	20<br>Ac-(Z)-ΔAla(βCl)-NHMe (1) chloroform C5 M062X/6-311+G(d,p) Charge = 0 Multiplicity = 1 Electronic Energy= -954.169485309 Electronic_Energy_with_ZeroPoint_Correction= -954.014854 Gibbs_Free_Energy= -954.055353 LowF= 47.0973 NImag= 0<br>N 0.0269360115 -0.0369491989 -0.0094965609<br>H 0.0449354138 -0.0727783901 0.9974899924<br>C 1.2844044056 -0.0827350603 -0.7393132642<br>H 2.0526815089 -0.479508991 -0.0801048354<br>H 1.1828348466 -0.7336625051 -1.6072568587<br>H 1.5761862848 0.9123358925 -1.081202991<br>C -1.1102709662 0.3164696527 -0.6330799844

	O -1.1625251232 0.6133435811 -1.8191890773<br>C -2.3814933853 0.3168870623 0.1769158407<br>N -3.3578731952 1.1828380144 -0.3329071928<br>H -3.2966293109 1.3283605215 -1.3337230726<br>C -4.0017374242 2.1459732481 0.416762248<br>O -3.8398806049 2.2591730322 1.6135054168<br>C -4.953591423 3.0121885791 -0.3701233893<br>H -5.0209293574 3.986040072 0.1098645847<br>H -4.6509150706 3.1279432235 -1.4104700584<br>H -5.9404374074 2.5442207451 -0.3483036914<br>C -2.5639351284 -0.5344262804 1.1866337617<br>H -1.7848449149 -1.1984904894 1.5308196728<br>Cl -4.0628086468 -0.7510065964 2.0022494053
conformation C5'	20<br>Ac-(Z)-ΔAla(βCl)-NHMe (1) chloroform M062X/6-311+G(d,p) Charge = 0 Multiplicity = 1 Electronic Energy= -954.168360511 Electronic_Energy_with_ZeroPoint_Correction= -954.013751 Gibbs_Free_Energy= -954.054719 LowF= 31.4987 NImag= 0<br>N 0.0071765762 -0.0540448514 -0.0167856792<br>H 0.0209638145 -0.084828713 0.9900339375<br>C 1.2698782426 -0.0751628744 -0.7370855939<br>H 2.0398383109 -0.4658778994 -0.0760462876<br>H 1.1844647555 -0.7202664062 -1.6111579614<br>H 1.5495767354 0.9266580575 -1.0699808981<br>C -1.1328262821 0.278376836 -0.6523356289<br>O -1.1711143201 0.5866734318 -1.8343743044<br>C -2.4028567614 0.2704791062 0.1643819571<br>N -3.4234812981 1.0663055526 -0.3638475468<br>H -3.1118366738 1.881687204 -0.8751765745<br>C -4.6680265073 0.5730819986 -0.7229346942<br>O -5.0132385282 -0.5627565662 -0.4853072479<br>C -5.5588725223 1.5897417997 -1.3920759022<br>H -6.3606162297 1.0685607837 -1.9091242433<br>H -5.9903897667 2.2374940748 -0.6257114466<br>H -5.0066010022 2.2138661452 -2.0953108998<br>C -2.5200769635 -0.4752787131 1.2653225392<br>H -1.75413268 -1.1694335336 1.5781252675<br>Cl -3.8806521433 -0.4344981889 2.3177697579
**Water**	
conformation α	20<br>Ac-(Z)-ΔAla(βCl)-NHMe (1) water α M062X/6-311+G(d,p) Charge = 0 Multiplicity = 1 Electronic Energy= -954.176427838 Electronic_Energy_with_ZeroPoint_Correction= -954.021976 Gibbs_Free_Energy= -954.062676 LowF= 41.6878 NImag= 0<br>N -0.1611000086 0.3355663691 -0.1762306103<br>H -0.3984164304 1.1619196306 0.3509965585<br>C 1.1904654971 0.1650173432 -0.6795066135<br>H 1.7868735931 1.0182925502 -0.3666515465<br>H 1.6317311959 -0.7497438458 -0.2803946906<br>H 1.1899637871 0.1052390529 -1.7690268204<br>C -1.1038502378 -0.5913292335 -0.4010235732<br>O -0.9080131986 -1.6059466119 -1.0588809473<br>C -2.4645603582 -0.2778857284 0.1696082456<br>N -2.5570661971 0.5854974477 1.2756415787<br>H -3.2852706 1.2876991217 1.2710897411<br>C -1.9444093526 0.2848303838 2.4722115365<br>O -1.2420147052 -0.7010111268 2.5914067425<br>C -2.161423646 1.2835248773 3.5771706031<br>H -1.3141125428 1.9730450029 3.5838863205<br>H -3.0763597539 1.8600094874 3.4484530371<br>H -2.1830658572 0.7555216915 4.5283384998<br>C -3.5224733565 -0.7693210606 -0.4704231317<br>H -3.4240177031 -1.3698579252 -1.3624087934<br>Cl -5.1432786316 -0.4665457358 0.0358385225
conformation β	20<br>Ac-(Z)-ΔAla(βCl)-NHMe (1) water β M062X/6-311+G(d,p) Charge = 0 Multiplicity = 1 Electronic Energy= -954.176220523 Electronic_Energy_with_ZeroPoint_Correction= -954.021786 Gibbs_Free_Energy= -954.062248 LowF= 50.4857 NImag= 0<br>N -0.1015352387 -0.2689422919 -0.0128895122<br>H -0.2136112984 -0.5941285319 0.9351231173<br>C 1.222300243 -0.2575267091 -0.612705465

	H 1.9607206174 -0.3834956611 0.1751903066<br>H 1.3266689765 -1.065284227 -1.3399984109<br>H 1.3943446711 0.692733941 -1.1184867725<br>C -1.1972052673 -0.0530418809 -0.7574545873<br>O -1.171786815 0.1863835244 -1.9552019868<br>C -2.5056132613 -0.0487065939 0.0012717046<br>N -3.5890924597 -0.6728358767 -0.6155864155<br>H -4.5055919138 -0.2588245515 -0.5108146272<br>C -3.4771180141 -1.8859808228 -1.2506489681<br>O -2.4310554628 -2.5076121862 -1.2521388348<br>C -4.7211438118 -2.3557313476 -1.9557552944<br>H -4.7371769012 -3.4433462522 -1.9593735218<br>H -5.6294141899 -1.9697358753 -1.4943150792<br>H -4.6822743551 -2.0036633516 -2.9892435083<br>C -2.6082634158 0.6716319562 1.1179979365<br>H -1.7896641231 1.2413050667 1.5313279251<br>Cl -4.1010027613 0.8325812328 1.9810501437
conformation β2	20<br>Ac-(Z)-ΔAla(βCl)-NHMe (1) water β2 M062X/6-311+G(d,p) Charge = 0 Multiplicity = 1 Electronic Energy= -954.175324215 Electronic_Energy_with_ZeroPoint_Correction= -954.020958 Gibbs_Free_Energy= -954.061634 LowF= 39.3618 NImag= 0<br>N -0.0298507836 -0.0532750695 0.0256033313<br>H -0.0475391923 -0.1145165694 1.0320173887<br>C 1.2431152432 -0.044688107 -0.6738325743<br>H 2.0415544166 0.0307126287 0.0600028163<br>H 1.3711727124 -0.9593186115 -1.255676823<br>H 1.2930187259 0.8085095556 -1.3512250416<br>C -1.1888302771 -0.0710051177 -0.6511810191<br>O -1.2499679836 -0.066738443 -1.8756759071<br>C -2.4308159011 -0.0988493038 0.2012878388<br>N -2.3110888201 0.0970531837 1.5944261934<br>H -2.0501408544 1.0164881326 1.9260270554<br>C -2.6383118358 -0.8624471574 2.5244339445<br>O -2.9586307617 -1.9903461683 2.2040389743<br>C -2.586028861 -0.3948741828 3.9558916952<br>H -2.3675449752 -1.2449598778 4.5982361312<br>H -1.8456448593 0.3896687064 4.1092573938<br>H -3.5694193346 0.0005087544 4.2217048616<br>C -3.5864351447 -0.3496122638 -0.4098475777<br>H -3.6304021895 -0.5433095157 -1.4718691922<br>Cl -5.0996592831 -0.3882354494 0.4016757722
conformation C5	20<br>Ac-(Z)-ΔAla(βCl)-NHMe (1) water C5 M062X/6-311+G(d,p) Charge = 0 Multiplicity = 1 Electronic Energy= -954.173432608 Electronic_Energy_with_ZeroPoint_Correction= -954.018863 Gibbs_Free_Energy= -954.059341 LowF= 48.0322 NImag= 0<br>N -0.044095742 -0.0216915465 0.0017265162<br>H -0.0647990298 -0.0651863199 1.0086353373<br>C 1.2389091941 0.1145869103 -0.6706674656<br>H 2.0272972428 -0.1069425127 0.0440919712<br>H 1.2991896923 -0.5870575354 -1.5025393673<br>H 1.3713262479 1.1276223476 -1.0551616728<br>C -1.1913429661 0.1297887949 -0.6760272631<br>O -1.2389093882 0.3970330919 -1.8714755682<br>C -2.4826260123 -0.0651860433 0.0777638218<br>N -3.5611913447 0.6303025698 -0.4867992942<br>H -3.4902532595 0.7623157213 -1.4888781739<br>C -4.3825164851 1.4853530626 0.2138596606<br>O -4.2939748325 1.6390403128 1.4162037727<br>C -5.425090298 2.17866239 -0.6261050981<br>H -5.6446870117 3.1487080564 -0.1851336797<br>H -5.1142725458 2.3013973154 -1.6628936166<br>H -6.335411171 1.5749633199 -0.6079095549<br>C -2.5734871721 -0.9281734045 1.0895869469<br>H -1.7171916518 -1.4582196406 1.4798390339<br>Cl -4.0578877811 -1.3737542063 1.8393185736
conformation C5'	20<br>Ac-(Z)-ΔAla(βCl)-NHMe (1) water C5' M062X/6-311+G(d,p) Charge = 0 Multiplicity = 1 Electronic Energy= -954.172825942 Electronic_Energy_with_ZeroPoint_Correction= -954.018196 Gibbs_Free_Energy= -954.058805 LowF= 43.1216 NImag= 0

	N -0.003908442 -0.0174103896 -0.0043615589<br>H 0.0084152073 -0.0549958728 1.002827426<br>C 1.2601442381 -0.0178872139 -0.7228868366<br>H 2.0400229735 -0.3666416873 -0.0506127545<br>H 1.1982146003 -0.68737514 -1.5806555675<br>H 1.5086018636 0.9842484661 -1.0783295799<br>C -1.1530642511 0.2674780394 -0.6399639871<br>O -1.2093763252 0.5564671892 -1.8280888869<br>C -2.4174156807 0.2203316461 0.1840744396<br>N -3.4515611193 1.0371543321 -0.2881144718<br>H -3.168288608 1.9278237811 -0.6767327272<br>C -4.6733448032 0.5528228896 -0.7139888176<br>O -4.996463934 -0.6095011403 -0.5826335649<br>C -5.5525460888 1.5962052035 -1.356497363<br>H -6.5459993377 1.1811599443 -1.5046516747<br>H -5.6103387814 2.4912607564 -0.7362152651<br>H -5.1333817876 1.8791097689 -2.3248229181<br>C -2.5252632631 -0.5930359963 1.2358706834<br>H -1.7491366682 -1.2916535922 1.5122373674<br>Cl -3.8961786508 -0.6378926242 2.2773349627
Ac-(*E*)-ΔAla(βCl)-NHMe (**2**)	
**Gas phase**	
conformation C5	20<br>Ac-(E)-ΔAla(βCl)-NHMe (2) gas phase C5 M062X/6-311+G(d,p) Charge = 0 Multiplicity = 1 Electronic Energy= -954.161092043 Electronic_Energy_with_ZeroPoint_Correction= -954.005897 Gibbs_Free_Energy= -954.047138 LowF= 26.294 NImag= 0<br>N -0.0032793582 -0.0006045445 -0.0027396253<br>H -0.0092963423 -0.0021292609 1.002772259<br>C 1.2580569159 -0.0012411196 -0.7241344796<br>H 2.0704223613 -0.0061253993 -0.0005178526<br>H 1.3313671844 -0.8829756293 -1.3624285429<br>H 1.3362415115 0.8847297491 -1.3559884358<br>C -1.1559715899 0.0034359939 -0.6908150669<br>O -1.1751815456 0.0063526952 -1.9136926592<br>C -2.4850998092 0.0044066577 0.0521714756<br>N -3.5157698231 0.0081332042 -0.8959947181<br>H -3.1604178202 0.0092324793 -1.8459247917<br>C -4.8687743925 0.0072194409 -0.6830421297<br>O -5.3733873228 0.0062493369 0.4210825331<br>C -5.7073063154 0.0009551019 -1.9416716521<br>H -6.3997836527 0.8414679153 -1.8940437474<br>H -5.1195521983 0.0635952263 -2.8567838263<br>H -6.2979100541 -0.9159939099 -1.9529672946<br>C -2.7344495859 0.002589543 1.3690459862<br>H -3.7428705232 0.0039457518 1.7468901553<br>Cl -1.5510075901 -0.0019784455 2.6419226581
conformation β	20<br>Ac-(E)-ΔAla(βCl)-NHMe (2) gas phase β M062X/6-311+G(d,p) Charge = 0 Multiplicity = 1 Electronic Energy= -954.15218243 Electronic_Energy_with_ZeroPoint_Correction= -953.997834 Gibbs_Free_Energy= -954.038778 LowF= 39.1947 NImag= 0<br>N -0.0280826819 0.0915309083 0.037849112<br>H -0.06047592 0.2779372447 1.0275767277<br>C 1.24755506272 0.1019115091 -0.6542366238<br>H 2.038448549 -0.1164074108 0.060798237<br>H 1.2427397207 -0.6614309493 -1.4315791468<br>H 1.4317019378 1.0717849934 -1.1227951662<br>C -1.1748282158 0.1760096079 -0.6623945661<br>O -1.2366896423 0.1753385894 -1.8751211002<br>C -2.4534791127 0.2639272239 0.1490680663<br>N -3.3706364147 1.234514824 -0.3128761677<br>H -4.2304291099 0.9117739145 -0.7305715432<br>C -2.9194169457 2.5029886088 -0.6169699431<br>O -1.8289371593 2.87964581 -0.2550292569<br>C -3.8711506736 3.3464333685 -1.427239072<br>H -3.65399537 4.3960744033 -1.2441058853<br>H -3.7013296606 3.1315926686 -2.4850216581<br>H -4.9145625364 3.133746937 -1.190870791<br>C -2.8251900599 -0.5496208747 1.1343182383<br>H -3.7763336046 -0.4310789907 1.6352465146<br>Cl -1.9181917578 -1.909919342 1.6995863115

conformation C7	20<br>Ac-(E)-ΔAla(βCl)-NHMe (2) gas phase C7 M062X/6-311+G(d,p) Charge = 0 Multiplicity = 1 Electronic Energy= -954.151119295 Electronic_Energy_with_ZeroPoint_Correction= -953.996624 Gibbs_Free_Energy= -954.038459 LowF= 19.0045 NImag= 0<br>N -0.0116226052 -0.1290276259 0.0771708269<br>H -0.0270867856 -0.1591357449 1.0893384335<br>C 1.2353806107 -0.0426696457 -0.6599191883<br>H 2.061540045 -0.0658823765 0.0477190423<br>H 1.3265587926 -0.8805543123 -1.3531634151<br>H 1.2773412565 0.8821068261 -1.2387388608<br>C -1.1873675901 -0.1224077248 -0.5796845788<br>O -1.2949781493 -0.0550364276 -1.7883301404<br>C -2.4114526049 -0.1832890106 0.3156745879<br>N -2.7135007221 0.9627835242 1.1135153656<br>H -3.4014230201 1.6138801879 0.7658509686<br>C -2.0146211381 1.2724114435 2.2482552061<br>O -1.1213960453 0.563146307 2.6693302837<br>C -2.4146172291 2.5568742758 2.9330594848<br>H -1.6438220617 3.3042907257 2.735905551<br>H -3.3775366082 2.9410606038 2.5972773595<br>H -2.4420985182 2.3783580325 4.0067981568<br>C -3.2479537067 -1.2118016651 0.3391461979<br>H -4.1293263433 -1.2219127745 0.9664399653<br>Cl -3.0264160318 -2.6352004018 -0.6024468372
conformation β2	20<br>Ac-(E)-ΔAla(βCl)-NHMe (2) gas phase β2 M062X/6-311+G(d,p) Charge = 0 Multiplicity = 1 Electronic Energy= -954.149505838 Electronic_Energy_with_ZeroPoint_Correction= -953.994663 Gibbs_Free_Energy= -954.036003 LowF= 18.8032 NImag= 0<br>N 0.0645580403 -0.1463808406 -0.009786747<br>H 0.1592285226 -0.4116193419 0.9595242649<br>C 1.2673041912 -0.0242522437 -0.8186522739<br>H 2.0811760986 0.3337236599 -0.1906121469<br>H 1.5444754384 -0.9784329076 -1.2733190749<br>H 1.0853533318 0.6929427748 -1.6176565036<br>C -1.1280037372 -0.4166585896 -0.6225070529<br>O -1.2624786737 -0.4344678401 -1.8226447419<br>C -2.2741183549 -0.6157289867 0.3448471759<br>N -2.3429087615 0.3962445717 1.3242301868<br>H -1.7718864832 1.2091629618 1.1466700127<br>C -3.1897331848 0.4470214396 2.4092431424<br>O -3.9576950468 -0.445989988 2.68588313<br>C -3.1001838219 1.7209634265 3.2199958139<br>H -3.2190714751 1.4661694675 4.271525888<br>H -2.1641111388 2.2605847767 3.0741347001<br>H -3.9300411245 2.3705176781 2.9338081642<br>C -3.1039972925 -1.6589044282 0.2745576023<br>H -3.9168306376 -1.8093866049 0.964505916<br>Cl -2.9215711365 -2.914393871 -0.8994804971
conformation α	20<br>Ac-(E)-ΔAla(βCl)-NHMe (2) gas phase α M062X/6-311+G(d,p) Charge = 0 Multiplicity = 1 Electronic Energy= -954.147460081 Electronic_Energy_with_ZeroPoint_Correction= -953.992905 Gibbs_Free_Energy= -954.033653 LowF= 37.1103 NImag= 0<br>N -0.0213968558 -0.0206462096 -0.0057655077<br>H -0.0285952812 -0.0422777244 1.0007927761<br>C 1.2521982946 0.0136581339 -0.7011653231<br>H 1.9976942632 -0.505305268 -0.1011083985<br>H 1.1498455767 -0.4887313645 -1.662095737<br>H 1.5749189835 1.0425700367 -0.8815845003<br>C -1.1390411468 0.4122948102 -0.6438892631<br>O -1.1801111157 0.6551815398 -1.828556006<br>C -2.364001648 0.510476167 0.2361524164<br>N -2.1689602709 0.8699404017 1.5958788882<br>H -2.7719931962 0.4395144711 2.2813166063<br>C -1.5027489227 2.0302333586 1.9615418281<br>O -0.8998924406 2.6982009456 1.1583848194<br>C -1.5958611313 2.3722880028 3.4309994023<br>H -0.7312227219 2.9730948404 3.7037219716<br>H -2.4957933881 2.9700135925 3.5944276441<br>H -1.6480132484 1.4846343524 4.0626630143<br>C -3.5973277466 0.216806026 -0.1738269211

	H -4.4445648854 0.3288787529 0.4912309837<br>Cl -4.025525077 -0.4153905454 -1.7120023451
**Chloroform**	
conformation C5	20<br>Ac-(E)-ΔAla(βCl)-NHMe (2) chloroform C5 M062X/6-311+G(d,p) Charge = 0 Multiplicity = 1 Electronic Energy= -954.169746411 Electronic_Energy_with_ZeroPoint_Correction= -954.014752 Gibbs_Free_Energy= -954.055501 LowF= 29.7346 NImag= 0<br>N -0.0197190252 -0.1058132276 -0.0119511207<br>H -0.044988163 -0.3291269759 0.9690525132<br>C 1.2565976444 -0.0994401283 -0.7093528708<br>H 2.0436802136 -0.2944465028 0.0146609422<br>H 1.2746271228 -0.8680779461 -1.483518<br>H 1.4265167804 0.870303297 -1.1782148227<br>C -1.1511838973 0.1366007901 -0.6822624094<br>O -1.1600960609 0.3591909475 -1.8889390206<br>C -2.4831278431 0.1071129893 0.0528276341<br>N -3.5140203676 0.048021428 -0.8953536198<br>H -3.1698665225 0.1086226625 -1.8470939723<br>C -4.8617730536 -0.0334475061 -0.68733004<br>O -5.3645461118 -0.0723635038 0.4228617756<br>C -5.6978630467 -0.0947485918 -1.9426876492<br>H -6.5112405782 0.6241821435 -1.849355345<br>H -5.1310184522 0.1089983905 -2.8496133827<br>H -6.1363870276 -1.0917969404 -2.0118840641<br>C -2.7266933475 0.1624566318 1.3693335846<br>H -3.7312500691 0.1503075423 1.7553148248<br>Cl -1.5337420869 0.2889306519 2.6250078267
conformation β	20<br>Ac-(E)-ΔAla(βCl)-NHMe (2) chloroform β M062X/6-311+G(d,p) Charge = 0 Multiplicity = 1 Electronic Energy= -954.166358734 Electronic_Energy_with_ZeroPoint_Correction= -954.012118 Gibbs_Free_Energy= -954.052688 LowF= 47.3827 NImag= 0<br>N 0.0159344143 -0.0614983575 -0.0383449595<br>H 0.0247554985 -0.1719773187 0.9636297885<br>C 1.2672670768 -0.069077374 -0.7754398631<br>H 2.0642437907 -0.37852192 -0.1036328932<br>H 1.2070750536 -0.768435284 -1.6099980703<br>H 1.4889357881 0.9252179175 -1.1686385153<br>C -1.1389927179 0.2377046954 -0.6477621699<br>O -1.2384452299 0.4874265025 -1.8381642164<br>C -2.3771334156 0.2282586791 0.2264414912<br>N -3.2106779126 1.3522700134 0.090905315<br>H -4.2058924042 1.2091307063 0.0032421426<br>C -2.7089731704 2.6284888865 0.0435626942<br>O -1.5255480157 2.8490364376 0.2146670978<br>C -3.7160272084 3.7059994058 -0.2634944669<br>H -3.4016377997 4.6291275439 0.2185646581<br>H -3.7284817486 3.8645813716 -1.344243166<br>H -4.722227201 3.4402678319 0.0596922712<br>C -2.7861461329 -0.8008445586 0.9672142318<br>H -3.7066069131 -0.768671782 1.5340994115<br>Cl -1.9821277359 -2.332338995 1.0559836763
conformation α	20<br>Ac-(E)-ΔAla(βCl)-NHMe (2) chloroform α M062X/6-311+G(d,p) Charge = 0 Multiplicity = 1 Electronic Energy= -954.164624522 Electronic_Energy_with_ZeroPoint_Correction= -954.010348 Gibbs_Free_Energy= -954.051228 LowF= 38.1378 NImag= 0<br>N -0.0227839625 -0.057261555 -0.012933627<br>H -0.0330497985 -0.1462593625 0.9911023167<br>C 1.2443423897 -0.0253986848 -0.7209778154<br>H 2.0356159254 -0.3131291296 -0.0327550085<br>H 1.2243367464 -0.7229114149 -1.5588505958<br>H 1.4452342662 0.9762512991 -1.1074710365<br>C -1.1746531054 0.2090052412 -0.6577373853<br>O -1.2432334041 0.3962074541 -1.8600572238<br>C -2.3983791943 0.2196842295 0.2279605908<br>N -2.2448654641 0.7632098618 1.5254954419<br>H -2.7251698807 0.3120501976 2.2914134028<br>C -1.7443858866 2.0324134142 1.7218606803<br>O -1.3170282872 2.6930424699 0.7974312043<br>C -1.7792694266 2.5221182648 3.1465755872

	H -0.9481307756 3.2065662525 3.3028706791<br>H -2.7113931217 3.0711586025 3.2989435236<br>H -1.732700433 1.7064558608 3.8672746701<br>C -3.5694716037 -0.313179135 -0.1151658905<br>H -4.4239956572 -0.2708229854 0.5471048015<br>Cl -3.8757887828 -1.1904598591 -1.5676128164
conformation C7	20<br>Ac-(E)-ΔAla(βCl)-NHMe (2) chloroform C7 M062X/6-311+G(d,p) Charge = 0 Multiplicity = 1 Electronic Energy= -954.164378798 Electronic_Energy_with_ZeroPoint_Correction= -954.010048 Gibbs_Free_Energy= -954.051637 LowF= 24.2276 NImag= 0<br>N -0.0124352556 -0.0530041219 0.0570149683<br>H -0.0239108524 -0.0732983078 1.0705658287<br>C 1.2398899178 -0.0223530514 -0.6783880412<br>H 2.0605960481 -0.0234842536 0.0348737127<br>H 1.3243818381 -0.895044846 -1.3280845384<br>H 1.2986745686 0.8751159224 -1.2966163317<br>C -1.1869097608 -0.0470830416 -0.5895690312<br>O -1.2959845061 -0.032444081 -1.8069317414<br>C -2.4107408038 -0.0367510434 0.3058417219<br>N -2.6084979658 1.0910655984 1.1591215785<br>H -3.303833555 1.7717735174 0.8884800513<br>C -1.9216213775 1.2698090042 2.3216235388<br>O -1.070278574 0.4752475753 2.6949048316<br>C -2.2647553537 2.5064920084 3.1096252548<br>H -1.3852494517 3.1516475538 3.1317010975<br>H -3.1065598808 3.0589871084 2.6957378766<br>H -2.4887797066 2.2089992042 4.1338456958<br>C -3.3421942173 -0.9815298534 0.2834131825<br>H -4.2206755237 -0.9389113752 0.9130556261<br>Cl -3.2639019972 -2.377444836 -0.7266666687
conformation β2	20<br>Ac-(E)-ΔAla(βCl)-NHMe (2) chloroform β2 M062X/6-311+G(d,p) Charge = 0 Multiplicity = 1 Electronic Energy= -954.163997806 Electronic_Energy_with_ZeroPoint_Correction= -954.009433 Gibbs_Free_Energy= -954.051019 LowF= 19.0846 NImag= 0<br>N 0.0349851546 -0.1732363132 -0.0488692946<br>H 0.1012247473 -0.483564788 0.9092579976<br>C 1.2502034671 -0.0278685159 -0.8347105688<br>H 2.0983970943 0.0254147397 -0.1563620045<br>H 1.3800465467 -0.8714237854 -1.5156091359<br>H 1.1989234055 0.8886580961 -1.4219968862<br>C -1.1769180656 -0.1390069611 -0.6418501652<br>O -1.3369801987 0.0631590286 -1.8310471422<br>C -2.3388428556 -0.318233171 0.3142959941<br>N -2.300844373 0.5790077873 1.3950950766<br>H -1.6274576166 1.3285573865 1.3217684062<br>C -3.120455833 0.5848285374 2.4974927384<br>O -3.9869898084 -0.2498739202 2.6708368874<br>C -2.8790673208 1.7184556305 3.4632275618<br>H -2.9962795516 1.3393163816 4.4769290427<br>H -1.8963377098 2.1742615937 3.3503777961<br>H -3.6427668062 2.4805760735 3.2942614329<br>C -3.2751256468 -1.2515743881 0.1305215558<br>H -4.110462581 -1.3979930224 0.7924725272<br>Cl -3.2060398224 -2.3827089108 -1.1839501739
**Water**	
conformation C5	20<br>Ac-(E)-ΔAla(βCl)-NHMe (2) water C5 M062X/6-311+G(d,p) Charge = 0 Multiplicity = 1 Electronic Energy= -954.172459818 Electronic_Energy_with_ZeroPoint_Correction= -954.017642 Gibbs_Free_Energy= -954.058533 LowF= 25.6187 NImag= 0<br>N 0.0060406824 -0.0350162675 -0.0019503024<br>H 0.0107109964 -0.089536617 1.0037870187<br>C 1.2725971428 -0.0240041891 -0.7177387387<br>H 2.0737655752 -0.1714680172 0.0016070446<br>H 1.2954521133 -0.8265126807 -1.4555104002<br>H 1.4146091355 0.9280608424 -1.231518205<br>C -1.1459933497 0.0962750801 -0.6642410101<br>O -1.1946899581 0.2472558977 -1.8819545754<br>C -2.4502984796 0.106953469 0.1151459162<br>N -3.4234671684 0.7756321061 -0.6414584588

	H -3.1029961271 1.0153120854 -1.5725383461<br>C -4.7154172983 1.0609339628 -0.3044278669<br>O -5.1990376596 0.7718814208 0.7786294321<br>C -5.5182942263 1.7516031329 -1.3787282427<br>H -6.0641465355 2.5776908298 -0.9244941676<br>H -4.9079737741 2.1228334423 -2.2002123615<br>H -6.2472433007 1.0393972181 -1.7701431947<br>C -2.7163029382 -0.4548993414 1.3013947472<br>H -3.6948367821 -0.4024936432 1.7458546353<br>Cl -1.5903043441 -1.3624642422 2.2629896507
conformation β	20<br>Ac-(E)-ΔAla(βCl)-NHMe (2) water β M062X/6-311+G(d,p) Charge = 0 Multiplicity = 1 Electronic Energy= -954.171095493 Electronic_Energy_with_ZeroPoint_Correction= -954.016843 Gibbs_Free_Energy= -954.057366 LowF= 47.3011 NImag= 0<br>N 0.0412125016 -0.1991745347 -0.1513170098<br>H 0.0597148914 -0.6281665046 0.761173191<br>C 1.242622318 -0.1957838211 -0.9680015691<br>H 2.0029009375 -0.7899799895 -0.4674769832<br>H 1.0349861183 -0.6272575548 -1.9480363211<br>H 1.6114346346 0.8225813544 -1.1049506713<br>C -1.0697638869 0.4296591781 -0.5513851865<br>O -1.1681806755 1.0353108864 -1.6092678649<br>C -2.2608003451 0.3357636069 0.379673169<br>N -2.9110929742 1.5523939412 0.6493686468<br>H -3.9208346516 1.5658950199 0.6620109<br>C -2.2269016148 2.7053535527 0.9304137114<br>O -1.0120089978 2.7165997515 1.0212637962<br>C -3.0764427965 3.9399728129 1.078272348<br>H -2.6021664888 4.6104407004 1.7918436576<br>H -3.1235075328 4.4414183226 0.1088685729<br>H -4.0919071682 3.7113352722 1.3997026585<br>C -2.8011257877 -0.8019271991 0.8151694113<br>H -3.6844682764 -0.8157923064 1.438953931<br>Cl -2.2469593696 -2.3842308919 0.3796951139
conformation α	20<br>Ac-(E)-ΔAla(βCl)-NHMe (2) water α M062X/6-311+G(d,p) Charge = 0 Multiplicity = 1 Electronic Energy= -954.17072048 Electronic_Energy_with_ZeroPoint_Correction= -954.016516 Gibbs_Free_Energy= -954.0574 LowF= 35.7979 NImag= 0<br>N 0.0311431741 0.1127804201 0.0364683434<br>H 0.0115296535 0.2495473621 1.0356939812<br>C 1.3064285352 0.02757178 -0.6530034626<br>H 2.1028790733 0.0561139166 0.0863126857<br>H 1.3729686568 -0.9029950624 -1.2183140096<br>H 1.4239430191 0.8646895541 -1.3439587587<br>C -1.1257660837 0.099033047 -0.6457948675<br>O -1.1914365937 -0.041491105 -1.8579489163<br>C -2.3591630461 0.2439871408 0.2143037096<br>N -2.2822220338 1.1581867475 1.2891467098<br>H -2.6887800663 0.8954787811 2.1761292547<br>C -1.8816305267 2.4587665258 1.0998366845<br>O -1.5459122084 2.8605493365 0.00070933<br>C -1.8381307214 3.3094376091 2.3408822739<br>H -0.8351068784 3.2415853206 2.7685289742<br>H -2.0274288834 4.3443851786 2.0643207596<br>H -2.5573140044 2.9860136964 3.0923810423<br>C -3.4623166072 -0.4854664825 0.0614156058<br>H -4.3280349724 -0.3441022969 0.6944059456<br>Cl -3.6416489591 -1.781942967 -1.0648258202
conformation β2	20<br>Ac-(E)-ΔAla(βCl)-NHMe (2) water β2 M062X/6-311+G(d,p) Charge = 0 Multiplicity = 1 Electronic Energy= -954.168891211 Electronic_Energy_with_ZeroPoint_Correction= -954.01431 Gibbs_Free_Energy= -954.055626 LowF= 25.2846 NImag= 0<br>N 0.0264306313 -0.1664739822 -0.0576812812<br>H 0.0845083726 -0.4687620054 0.9038854364<br>C 1.2496566423 -0.0215298197 -0.8314992907<br>H 2.0947416166 -0.0372717379 -0.1480156115<br>H 1.3514386325 -0.833032291 -1.5544114181<br>H 1.2365477678 0.9269825857 -1.3682834075<br>C -1.1797334521 -0.0709005475 -0.644616136

	O -1.3362683389 0.1647804949 -1.8313555145<br>C -2.3501256551 -0.2316647476 0.3051736891<br>N -2.2807507971 0.6293174851 1.4116647558<br>H -1.5711264689 1.3478802225 1.3697084407<br>C -3.0985851923 0.6304921939 2.5140538448<br>O -4.0030445413 -0.1723039768 2.6551987985<br>C -2.808568657 1.7139151605 3.5211188468<br>H -2.9444152256 1.3032641925 4.5201204586<br>H -1.8061211438 2.1278321106 3.4247676334<br>H -3.5361389216 2.5159974729 3.379525349<br>C -3.3229428312 -1.1204805127 0.0904892249<br>H -4.1668063061 -1.2538778826 0.7438237989<br>Cl -3.2942341027 -2.2177198645 -1.256493084
conformation C7	20<br>Ac-(E)-ΔAla(βCl)-NHMe (2) water C7 M062X/6-311+G(d,p) Charge = 0 Multiplicity = 1 Electronic Energy= -954.168567286 Electronic_Energy_with_ZeroPoint_Correction= -954.014132 Gibbs_Free_Energy= -954.054844 LowF= 33.8518 NImag= 0<br>N -0.0095091606 -0.0168163908 0.0478226987<br>H -0.0168523992 -0.024785539 1.0619250952<br>C 1.2425618426 -0.0109617566 -0.6893558945<br>H 2.0630826231 0.0226587399 0.0230068547<br>H 1.3332014028 -0.9097817684 -1.3016677256<br>H 1.2959238366 0.8611854161 -1.3429407909<br>C -1.185163794 -0.0251871913 -0.5930995297<br>O -1.2968329849 -0.0471052423 -1.8124694339<br>C -2.4061194601 0.018459022 0.3039513631<br>N -2.5610755064 1.1386150271 1.175927641<br>H -3.2471169704 1.8374836285 0.927132631<br>C -1.8825684867 1.2661644045 2.3479763446<br>O -1.0526732859 0.4381857028 2.7040434806<br>C -2.2080582475 2.4811250405 3.1742717528<br>H -1.2902764473 3.0507614869 3.3233317112<br>H -2.9623437954 3.1194149415 2.7179377156<br>H -2.5581366165 2.1452530799 4.1509212361<br>C -3.3739243396 -0.889418839 0.2714427974<br>H -4.2478514186 -0.8208787712 0.9051093648<br>Cl -3.356767564 -2.2762033499 -0.756002424

Table 2S. Structural parameters for the internal hydrogen bond X–H⋯A (X = N, C; A = O, Cl) and C=O ◄⋯► O=C interactions in the M06-2X/6-311+G(d,p) conformers of Ac-(*Z*)-ΔAla(βCl)-NHMe (**1**) in various environment.

**Ac-(*Z*)-ΔAla(βCl)-NHMe (1)**															
	**C7**		**β**			**C5**			**C5'**			**α**		**β2**	
	Gas Phase	*Chloroform*	*Gas Phase*	*Chloroform*	*Water*	*Gas Phase*	*Chloroform*	*Water*	*Gas Phase*	*Chloroform*	*Water*	*Chloroform*	*Water*	*Chloroform*	*Water*
	**Hydrogen bond**														
**N^{N}-H⋯O^{C}**															
r H⋯O						2.27	2.30	2.31	2.30	2.52					
r N⋯C						2.42	2.43	2.43	2.42	2.44					
∠N-H⋯O						104.1	102.7	102.2	100.8	90.9					
∠C=O⋯H						84.8	84.9	84.9	82.5	77.6					
**N^{C}-H⋯O^{N}**															
r H⋯O	1.90	2.03													
r N⋯C	3.18	3.18													
∠N-H⋯O	149.8	142.1													
∠C=O⋯H	102.4	94.0													
**N^{N}-H⋯Cl**															
r H⋯Cl	2.67	2.81	2.75	2.75	2.75							2.79	2.84		
r N⋯Cl	3.00	3.01	3.05	3.05	3.04							3.05	3.05		
∠N-H⋯Cl	99.1	91.7	97.7	97.2	97.1							95.1	92.5		
∠C-Cl⋯H	68.1	67.0	68.8	68.8	68.8							68.1	67.7		
**C$^{\beta}$-H⋯O^{C}**															
r H⋯O	2.38	2.41										2.52	2.55	2.46	2.46
r C⋯O	2.71	2.73										2.79	2.81	2.77	2.77
∠C-H⋯O	96.0	95.4										93.0	92.5	95.0	95.0
∠C=O⋯H	85.7	85.1										81.0	80.2	83.2	83.3
	**C=O ◄⋯► O=C dipole interaction**														
r O^{N}⋯C^{C}	3.29	3.26	2.74	2.77	2.79							3.01	3.00		
r O^{C}⋯C^{N}			3.18	3.17	3.18										
r C^{N}⋯C^{C}	3.33	3.31	2.92	2.95	2.97							3.14	3.12		
r O^{N}⋯O^{C}	4.47	4.42	3.06	3.05	3.06							3.82	3.78		
∠(C=O)^{N}⋯C^{C}	81.3	81.3	86.5	86.2	86.0							84.7	84.2		
∠(C=O)^{C}⋯C^{N}	11.3	12.0	67.0	68.5	68.9							27.8	28.7		
∠O^{C}⋯(C=O)^{N}	80.2	78.5	73.6	73.1	73.1							65.1	64.4		
∠O^{N}⋯(C=O)^{C}	163.7	157.2	93.7	91.1	90.4							123.4	120.9		
**Type**	**I**		**II**									**III**			

Data presented only for X–H⋯A (X = N, C; A = O, Cl) in which *r* H⋯X ≤ 2.7 Å, *r* H⋯Cl ≤ 3.3 Å and ∠X–H⋯A > 90° (Vargas 2002)
Data presented only for the C=O ◄⋯► O=C contacts in which *r* C⋯O < 3.6 Å (Allen 1998)
$^{N, C}$ denote the N-terminal or the C-terminal amide group

Table 3S. Structural parameters for the internal hydrogen bond X–H···A (X = N, C; A = O, Cl) and C=O ◄···► O=C interactions in the M06-2X/6-311+G(d,p) conformers of Ac-(*E*)-ΔAla(βCl)-NHMe (**2**) in various environment.

Ac-(*E*)-ΔAla(βCl)-NHMe (2)															
	**C5**			**β**			**C7**			**β2**			**α**		
	Gas Phase	*Chloroform*	*Water*	*Gas Phase*	*Chloroform*	*Water*	*Gas Phase*	*Chloroform*	*Water*	*Gas Phase*	*Chloroform*	*Water*	*Gas Phase*	*Chloroform*	*Water*
	**Hydrogen bond**														
**N^N-H···O^C**															
r H···O	1.99	2.03	2.08												
r N···C	2.37	2.37	2.38												
∠N-H···O	112.5	111.3	109.8												
∠C=O···H	88.9	88.0	86.2												
**N^C-H···O^N**															
r H···O							2.05	2.01	2.00						
r N···C							3.27	3.24	3.23						
∠N-H···O							140.1	144.0	145.5						
∠C=O···H							109.3	107.1	105.6						
**N^C-H···Cl**															
r H···Cl	2.25	2.31	2.40	2.95	2.95	2.92									
r N···Cl	3.06	3.07	3.07	3.22	3.22	3.21									
∠N-H···Cl	137.1	130.9	123.3	96.1	96.1	97.1									
∠C-Cl···H	86.2	84.5	82.2	70.5	70.5	70.8									
**$C^β$-H···O^N**															
r H···O	2.10	2.12	2.14							2.20	2.20	2.20			
r C···O	2.80	2.81	2.82							2.83	2.82	2.82			
∠C-H···O	120.3	119.7	118.6							115.4	114.2	114.1			
∠C=O···H	104.6	104.6	104.6							105.5	106.0	106.2			
	**C=O ◄···► O=C dipole interaction**														
r O^N···C^C				2.81	2.78	2.78	3.32	3.33	3.33				2.92	2.88	2.87
r O^C···C^N				3.14	3.21	3.22							4.04	3.97	3.93
r C^N···C^C				2.91	2.94	2.95	3.26	3.28	3.29				3.09	3.05	3.03
r O^N···O^C				3.21	3.14	3.13	4.50	4.54	4.55				3.63	3.51	3.46
∠$(C=O)^N$···C^C				82.4	85.5	86.0	76.5	77.1	77.3				86.4	86.1	85.8
∠$(C=O)^C$···C^N				68.1	66.5	66.5	25.9	22.5	20.9				32.2	34.9	36.0
∠O^C···$(C=O)^N$				82.4	76.0	74.7	91.3	89.7	89.0				61.6	59.5	58.8
∠O^N···$(C=O)^C$				97.7	95.7	94.8	164.4	169.8	172.4				116.7	111.3	109.1
**Type**					**II**			**I**						**III**	

Data presented only for X–H···A (X = N, C; A = O, Cl) in which *r* H···X ≤ 2.7 Å, *r* H···Cl ≤ 3.3 Å and ∠X–H···A > 90° (Vargas 2002)
Data presented only for the C=O ◄···► O=C contacts in which *r* C···O < 3.6 Å (Allen 1998)
N, C denote the N-terminal or the C-terminal amide group

Table 4S. Crystal Parameters and Experimental Details of X-Ray Data Collection for the Studied Compounds.

	Ac-(Z)-ΔAla(βCl)-NHMe (**1**)	Cbz-Gly-(Z)-ΔAla(βCl)-Gly-OMe (**3**)	Boc-(Z)-ΔAla(βCl)-OMe (**4**)	Ac-(E)-ΔAla(βCl)-NHMe (**2**)	Ac-ΔAla(βCl₂)-NHMe (**5**)
Chemical formula	$C_6H_9ClN_2O_2$	$C_{16}H_{18}ClN_3O_6$	$C_{11}H_{17}ClN_2O_5$	$C_6H_9ClN_2O_2$	$C_6H_8Cl_2N_2O_2$
M_r	176.60	383.78	292.71	176.60	211.04
Crystal system, space group	Monoclinic, $P2_1/c$	Triclinic, $P\bar{1}$	Triclinic, $P\bar{1}$	Triclinic, $P\bar{1}$	Monoclinic, $P2_1/c$
a, b, c (Å)	13.0735 (3), 14.2235 (2), 9.1292 (2)	4.9591 (1), 12.7541 (4), 14.7235 (8)	8.8797 (5), 9.3366 (5), 9.6867 (6)	8.7340 (7), 8.7933 (7), 11.7526 (10)	6.8554 (1), 15.3174 (3), 8.8543 (2)
α, β, γ (°)	90, 100.795 (2), 90	76.590 (4), 88.533 (3), 79.015 (2)	84.211 (5), 76.033 (5), 65.201 (5)	91.920 (7), 91.512 (7), 115.592 (8)	90, 99.030 (2), 90
V (Å^3)	1667.54 (6)	889.09 (6)	707.48 (8)	812.76 (13)	918.24 (3)
Z	8	2	2	4	4
μ (mm^{-1})	3.71	2.26	2.57	3.81	6.09
Crystal size (mm)	0.2 × 0.15 × 0.1	0.15 × 0.12 × 0.10	0.20 × 0.17 × 0.15	0.30 × 0.22 × 0.15	0.22 × 0.16 × 0.10
T_{min}, T_{max}	0.907, 1.000	0.754, 1.000	0.908, 1.000	0.694, 1.000	0.662, 1.000
No. of measured, independent and observed [$I > 2\sigma(I)$] reflections	10717, 3196, 3093	9575, 3352, 2774	7449, 2664, 2519	11162, 3119, 2954	4949, 1755, 1663
R_{int}	0.015	0.034	0.017	0.024	0.013
(sin θ/λ)$_{max}$ (Å^{-1})	0.621	0.622	0.623	0.624	0.621
$R[F^2 > 2\sigma(F^2)]$, $wR(F^2)$, S	0.041, 0.100, 1.08	0.069, 0.198, 1.05	0.028, 0.073, 1.07	0.115, 0.283, 1.16	0.026, 0.069, 1.05
No. of reflections	3196	3352	2664	3119	1755
No. of parameters	217	244	184	203	115
Δρ$_{max}$, Δρ$_{min}$ (e Å^{-3})	1.22, −0.49	1.37, −0.40	0.28, −0.28	1.51, −0.74	0.25, −0.30

Table 5S. Bond distances (Å), bond angles (°), and torsion angles (°) of the studied compounds as determined by X-ray method.

**Ac-(Z)-ΔAla(βCl)-NHMe (1)**			
Cl1—C3	1.720 (2)	O1—C1	1.233 (3)
Cl1'—C3'	1.722 (2)	O1'—C1'	1.238 (3)
N1—C1	1.323 (3)	O2—C4	1.233 (3)
N1—C6	1.445 (3)	O2'—C4'	1.232 (3)
N1'—C1'	1.329 (3)	C1—C2	1.517 (3)
N1'—C6'	1.450 (3)	C1'—C2'	1.511 (3)
N2—C4	1.345 (3)	C2—C3	1.328 (3)
N2—C2	1.402 (3)	C2'—C3'	1.331 (3)
N2'—C4'	1.357 (3)	C4—C5	1.518 (3)
N2'—C2'	1.400 (3)	C4'—C5'	1.501 (3)
C1—N1—C6	120.8 (2)	N2—C2—C1	116.40 (18)
C1'—N1'—C6'	122.9 (2)	C3'—C2'—N2'	123.69 (19)
C4—N2—C2	120.83 (19)	C3'—C2'—C1'	118.70 (19)
C4'—N2'—C2'	121.79 (19)	N2'—C2'—C1'	116.81 (18)
O1—C1—N1	125.1 (2)	C2—C3—Cl1	122.89 (18)
O1—C1—C2	118.91 (19)	C2'—C3'—Cl1'	122.53 (18)
N1—C1—C2	115.94 (19)	O2—C4—N2	121.8 (2)
O1'—C1'—N1'	124.7 (2)	O2—C4—C5	121.4 (2)
O1'—C1'—C2'	120.36 (19)	N2—C4—C5	116.80 (19)
N1'—C1'—C2'	114.90 (18)	O2'—C4'—N2'	121.3 (2)
C3—C2—N2	123.7 (2)	O2'—C4'—C5'	122.8 (2)
C3—C2—C1	119.2 (2)	N2'—C4'—C5'	115.95 (19)
C6—N1—C1—O1	-3.7 (3)	O1'—C1'—C2'—C3'	129.0 (2)
C6—N1—C1—C2	174.4 (2)	N1'—C1'—C2'—C3'	−48.0 (3)

C6'—N1'—C1'—O1'	−3.6 (4)	O1'—C1'—C2'—N2'	−41.2 (3)
C6'—N1'—C1'—C2'	173.3 (2)	N1'—C1'—C2'—N2'	141.8 (2)
C4—N2—C2—C3	142.3 (2)	N2—C2—C3—Cl1	−7.8 (3)
C4—N2—C2—C1	−47.5 (3)	C1—C2—C3—Cl1	−177.74 (15)
O1—C1—C2—C3	137.9 (2)	N2'—C2'—C3'—Cl1'	−4.1 (3)
N1—C1—C2—C3	−40.3 (3)	C1'—C2'—C3'—Cl1'	−173.59 (15)
O1—C1—C2—N2	−32.8 (3)	C2—N2—C4—O2	−12.5 (3)
N1—C1—C2—N2	149.00 (19)	C2—N2—C4—C5	166.96 (19)
C4'—N2'—C2'—C3'	148.8 (2)	C2'—N2'—C4'—O2'	−7.8 (3)
C4'—N2'—C2'—C1'	−41.6 (3)	C2'—N2'—C4'—C5'	172.91 (19)
**Boc-(*Z*)-ΔAla(βCl)-OMe (4)**			
Cl1—C3	1.7148 (12)	O4—C6	1.2293 (14)
N2—C4	1.3600 (15)	O5—C6	1.3421 (14)
N2—C2	1.4114 (15)	O5—C7	1.4743 (14)
N3—C6	1.3415 (16)	C1—C2	1.4936 (16)
N3—C5	1.4458 (15)	C2—C3	1.3282 (17)
O1—C1	1.2030 (15)	C4—C5	1.5254 (15)
O2—C4	1.2213 (15)	C7—C8	1.5150 (18)
O3—C1	1.3370 (15)	C7—C9	1.5199 (18)
O3—C11	1.4465 (15)	C7—C10	1.5213 (18)
C4—N2—C2	121.52 (10)	O2—C4—C5	122.28 (10)
C6—N3—C5	120.71 (10)	N2—C4—C5	114.32 (10)
C1—O3—C11	115.53 (10)	N3—C5—C4	112.03 (9)
C6—O5—C7	121.78 (9)	O4—C6—N3	123.74 (11)
O1—C1—O3	124.30 (11)	O4—C6—O5	125.63 (11)
O1—C1—C2	123.86 (11)	N3—C6—O5	110.63 (10)
O3—C1—C2	111.79 (10)	O5—C7—C8	110.59 (10)
C3—C2—N2	123.85 (11)	O5—C7—C9	109.80 (10)
C3—C2—C1	118.81 (11)	C8—C7—C9	112.82 (11)
N2—C2—C1	116.99 (10)	O5—C7—C10	102.23 (10)
C2—C3—Cl1	122.86 (10)	C8—C7—C10	110.43 (11)
O2—C4—N2	123.37 (11)	C9—C7—C10	110.48 (11)
C11—O3—C1—O1	3.88 (17)	C2—N2—C4—C5	−175.85 (10)
C11—O3—C1—C2	−173.74 (10)	C6—N3—C5—C4	83.69 (13)
C4—N2—C2—C3	−130.35 (13)	O2—C4—C5—N3	37.79 (15)
C4—N2—C2—C1	56.51 (15)	N2—C4—C5—N3	−144.11 (10)
O1—C1—C2—C3	−159.77 (12)	C5—N3—C6—O4	3.78 (17)
O3—C1—C2—C3	17.86 (16)	C5—N3—C6—O5	−176.20 (9)
O1—C1—C2—N2	13.73 (17)	C7—O5—C6—O4	7.36 (17)
O3—C1—C2—N2	−168.64 (10)	C7—O5—C6—N3	−172.67 (10)
N2—C2—C3—Cl1	1.31 (18)	C6—O5—C7—C8	−64.46 (14)
C1—C2—C3—Cl1	174.34 (9)	C6—O5—C7—C9	60.67 (14)
C2—N2—C4—O2	2.23 (18)	C6—O5—C7—C10	177.96 (10)
**Cbz-Gly-(*Z*)-ΔAla(βCl)-Gly-OMe (3)**			
Cl1—C3	1.717 (3)	O6—C15	1.330 (4)
N1—C1	1.342 (4)	O6—C16	1.451 (4)
N1—C14	1.443 (4)	C1—C2	1.506 (4)
N2—C4	1.369 (4)	C2—C3	1.336 (4)
N2—C2	1.401 (4)	C4—C5	1.520 (4)
N3—C6	1.325 (4)	C7—C8	1.504 (4)
N3—C5	1.442 (4)	C8—C9	1.350 (5)
O1—C1	1.227 (4)	C8—C13	1.367 (5)
O2—C4	1.217 (3)	C9—C10	1.397 (6)

O3—C6	1.231 (4)	C10—C11	1.341 (6)
O4—C6	1.354 (3)	C11—C12	1.337 (7)
O4—C7	1.451 (4)	C12—C13	1.388 (6)
O5—C15	1.211 (4)	C14—C15	1.511 (4)
C1—N1—C14	121.2 (3)	O3—C6—N3	125.7 (3)
C4—N2—C2	120.6 (2)	O3—C6—O4	123.1 (3)
C6—N3—C5	120.0 (2)	N3—C6—O4	111.1 (2)
C6—O4—C7	115.0 (2)	O4—C7—C8	106.9 (2)
C15—O6—C16	115.2 (3)	C9—C8—C13	117.7 (3)
O1—C1—N1	124.8 (3)	C9—C8—C7	121.8 (3)
O1—C1—C2	120.1 (2)	C13—C8—C7	120.5 (3)
N1—C1—C2	114.9 (2)	C8—C9—C10	120.7 (4)
C3—C2—N2	123.3 (3)	C11—C10—C9	120.8 (4)
C3—C2—C1	118.4 (3)	C12—C11—C10	119.0 (4)
N2—C2—C1	117.7 (2)	C11—C12—C13	120.9 (4)
C2—C3—Cl1	122.8 (2)	C8—C13—C12	120.8 (4)
O2—C4—N2	122.8 (3)	N1—C14—C15	114.1 (2)
O2—C4—C5	122.7 (3)	O5—C15—O6	122.9 (3)
N2—C4—C5	114.5 (2)	O5—C15—C14	123.7 (3)
N3—C5—C4	111.1 (2)	O6—C15—C14	113.4 (3)
C14—N1—C1—O1	5.3 (4)	C7—O4—C6—N3	−178.0 (2)
C14—N1—C1—C2	−170.2 (2)	C6—O4—C7—C8	174.3 (2)
C4—N2—C2—C3	−147.3 (3)	O4—C7—C8—C9	106.2 (4)
C4—N2—C2—C1	41.8 (4)	O4—C7—C8—C13	−74.4 (5)
O1—C1—C2—C3	−130.6 (3)	C13—C8—C9—C10	1.0 (8)
N1—C1—C2—C3	45.2 (4)	C7—C8—C9—C10	−179.6 (4)
O1—C1—C2—N2	40.8 (4)	C8—C9—C10—C11	−0.7 (9)
N1—C1—C2—N2	−143.5 (3)	C9—C10—C11—C12	1.1 (9)
N2—C2—C3—Cl1	5.1 (4)	C10—C11—C12—C13	−1.9 (9)
C1—C2—C3—Cl1	175.9 (2)	C9—C8—C13—C12	−1.8 (9)
C2—N2—C4—O2	8.5 (4)	C7—C8—C13—C12	178.9 (5)
C2—N2—C4—C5	−170.1 (2)	C11—C12—C13—C8	2.3 (10)
C6—N3—C5—C4	77.8 (3)	C1—N1—C14—C15	120.1 (3)
O2—C4—C5—N3	26.7 (4)	C16—O6—C15—O5	−2.4 (5)
N2—C4—C5—N3	−154.7 (2)	C16—O6—C15—C14	177.0 (3)
C5—N3—C6—O3	6.9 (4)	N1—C14—C15—O5	−173.1 (3)
C5—N3—C6—O4	−174.8 (2)	N1—C14—C15—O6	7.5 (4)
C7—O4—C6—O3	0.4 (4)		
**Ac-ΔAla(βCl_2)-NHMe (5)**			
Cl1—C3	1.7240 (16)	O1—C1	1.238 (2)
Cl2—C3	1.7167 (16)	O2—C4	1.225 (2)
N1—C1	1.326 (2)	C1—C2	1.511 (2)
N1—C6	1.455 (2)	C2—C3	1.337 (2)
N2—C4	1.363 (2)	C4—C5	1.498 (2)
N2—C2	1.406 (2)		
C1—N1—C6	121.34 (14)	N2—C2—C1	116.69 (13)
C4—N2—C2	122.21 (14)	C2—C3—Cl2	123.80 (13)
O1—C1—N1	124.26 (15)	C2—C3—Cl1	121.76 (13)
O1—C1—C2	119.51 (14)	Cl2—C3—Cl1	114.34 (9)
N1—C1—C2	116.21 (14)	O2—C4—N2	121.44 (16)
C3—C2—N2	120.62 (14)	O2—C4—C5	123.50 (16)
C3—C2—C1	122.12 (15)	N2—C4—C5	115.01 (15)
C6—N1—C1—O1	2.3 (3)	N1—C1—C2—N2	−129.81 (15)

C6—N1—C1—C2	−176.04 (14)	N2—C2—C3—Cl2	−170.03 (12)
C4—N2—C2—C3	−149.61 (16)	C1—C2—C3—Cl2	1.0 (2)
C4—N2—C2—C1	38.8 (2)	N2—C2—C3—Cl1	6.0 (2)
O1—C1—C2—C3	−119.68 (18)	C1—C2—C3—Cl1	177.07 (12)
N1—C1—C2—C3	58.8 (2)	C2—N2—C4—O2	4.7 (2)
O1—C1—C2—N2	51.7 (2)	C2—N2—C4—C5	−177.87 (14)
**Ac-(*E*)-ΔAla(βCl)-NHMe (2)**			
Cl1—C3	1.738 (8)	C1'—C2'	1.513 (10)
Cl1'—C3'	1.736 (8)	C2—C3	1.320 (11)
N1—C1	1.314 (10)	C2'—C3'	1.313 (11)
N1—C6	1.443 (10)	C3—H3	0.9500
N1—H1	0.8800	C3'—H3'	0.9500
N1'—C1'	1.321 (10)	C4—C5	1.519 (11)
N1'—C6'	1.455 (10)	C4'—C5'	1.502 (11)
N1'—H1'	0.8800	C5—H5C	0.9800
N2—C4	1.340 (10)	C5—H5B	0.9800
N2—C2	1.415 (9)	C5—H5A	0.9800
N2—H2	0.8800	C5'—H5B'	0.9800
N2'—C4'	1.350 (10)	C5'—H5A'	0.9800
N2'—C2'	1.425 (9)	C5'—H5C'	0.9800
N2'—H2'	0.8800	C6—H6B	0.9800
O1—C1	1.245 (9)	C6—H6A	0.9800
O1'—C1'	1.246 (9)	C6—H6C	0.9800
O2—C4	1.236 (10)	C6'—H6A'	0.9800
O2'—C4'	1.237 (10)	C6'—H6C'	0.9800
C1—C2	1.513 (10)	C6'—H6B'	0.9800
C1—N1—C6	121.7 (7)	O2—C4—N2	122.4 (7)
C1—N1—H1	119.2	O2—C4—C5	121.5 (7)
C6—N1—H1	119.2	N2—C4—C5	116.1 (7)
C1'—N1'—C6'	119.3 (7)	O2'—C4'—N2'	120.6 (7)
C1'—N1'—H1'	120.4	O2'—C4'—C5'	121.7 (7)
C6'—N1'—H1'	120.4	N2'—C4'—C5'	117.7 (7)
C4—N2—C2	121.5 (6)	C4—C5—H5C	109.5
C4—N2—H2	119.2	C4—C5—H5B	109.5
C2—N2—H2	119.2	H5C—C5—H5B	109.5
C4'—N2'—C2'	121.7 (6)	C4—C5—H5A	109.5
C4'—N2'—H2'	119.2	H5C—C5—H5A	109.5
C2'—N2'—H2'	119.2	H5B—C5—H5A	109.5
O1—C1—N1	122.4 (7)	C4'—C5'—H5B'	109.5
O1—C1—C2	119.3 (7)	C4'—C5'—H5A'	109.5
N1—C1—C2	118.2 (7)	H5B'—C5'—H5A'	109.5
O1'—C1'—N1'	122.8 (7)	C4'—C5'—H5C'	109.5
O1'—C1'—C2'	119.3 (6)	H5B'—C5'—H5C'	109.5
N1'—C1'—C2'	117.9 (7)	H5A'—C5'—H5C'	109.5
C3—C2—N2	117.9 (7)	N1—C6—H6B	109.5
C3—C2—C1	127.4 (7)	N1—C6—H6A	109.5
N2—C2—C1	114.1 (6)	H6B—C6—H6A	109.5
C3'—C2'—N2'	118.8 (7)	N1—C6—H6C	109.5
C3'—C2'—C1'	127.8 (7)	H6B—C6—H6C	109.5
N2'—C2'—C1'	113.1 (6)	H6A—C6—H6C	109.5
C2—C3—Cl1	123.2 (6)	N1'—C6'—H6A'	109.5
C2—C3—H3	118.4	N1'—C6'—H6C'	109.5
Cl1—C3—H3	118.4	H6A'—C6'—H6C'	109.5
C2'—C3'—Cl1'	123.0 (6)	N1'—C6'—H6B'	109.5
C2'—C3'—H3'	118.5	H6A'—C6'—H6B'	109.5
Cl1'—C3'—H3'	118.5	H6C'—C6'—H6B'	109.5

C6—N1—C1—O1	−3.2 (12)	O1'—C1'—C2'—C3'	126.4 (9)
C6—N1—C1—C2	178.3 (7)	N1'—C1'—C2'—C3'	−52.5 (11)
C6'—N1'—C1'—O1'	3.4 (11)	O1'—C1'—C2'—N2'	−47.0 (9)
C6'—N1'—C1'—C2'	−177.7 (6)	N1'—C1'—C2'—N2'	134.1 (7)
C4—N2—C2—C3	−138.4 (8)	N2—C2—C3—Cl1	−169.5 (5)
C4—N2—C2—C1	49.7 (9)	C1—C2—C3—Cl1	1.1 (12)
O1—C1—C2—C3	−123.5 (9)	N2'—C2'—C3'—Cl1'	172.3 (5)
N1—C1—C2—C3	55.1 (11)	C1'—C2'—C3'—Cl1'	−0.8 (12)
O1—C1—C2—N2	47.4 (9)	C2—N2—C4—O2	6.2 (11)
N1—C1—C2—N2	−134.0 (7)	C2—N2—C4—C5	−172.2 (7)
C4'—N2'—C2'—C3'	136.4 (8)	C2'—N2'—C4'—O2'	−7.1 (11)
C4'—N2'—C2'—C1'	−49.6 (9)	C2'—N2'—C4'—C5'	171.6 (7)

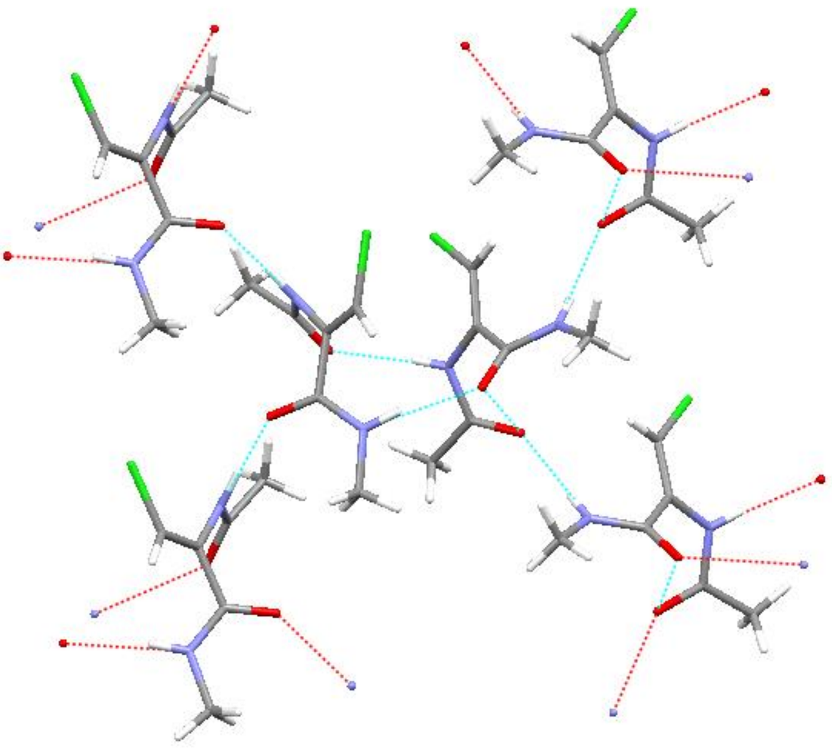

Figure 1S. Molecular interactions of the Ac-(Z)-ΔAla(βCl)-NHMe (**1**) molecules with visualization of the hydrogen bonds (dotted lines): Mercury 3.8 (Build RC2) (http://www.ccdc.cam.ac.uk/mercury/) was applied. The hydrogen bonds rely on the sum of van der Waals radii of the atom involved. Each molecule creates four N-H…O hydrogen bonds using both amide groups, and each amide plays both donor and acceptor role. Two similar conformations, lets name as β^1 (φ, ψ = -47.5°, 149.0°) and β^2 (φ, ψ = -41.6°, 141.75°) are present and the molecule with the conformation β^1 creates two N-H…O hydrogen bonds with the molecule with the conformation β^2, one is created between the N-terminal and another between the C-terminal amide groups. In the molecule β^1, the N-terminal amide is the acceptor and the C-terminal amide is the donor. On the contrary, in the molecule β^2, the N-terminal amide is the donor and the C-terminal amide is the acceptor. Additionally, each molecule β1 creates with two molecules -β1 (φ, ψ = 47.5°, -149.0°) two other N-H…O hydrogen bonds, in which the N-terminal amide group of the molecule β^1 is a donor for the C-terminal amide group of the molecule -β^1. Analogously, the molecule β^2 creates two N-H…O hydrogen bonds with two molecules -β^2 (φ, ψ = 41.6°, -141.75°).

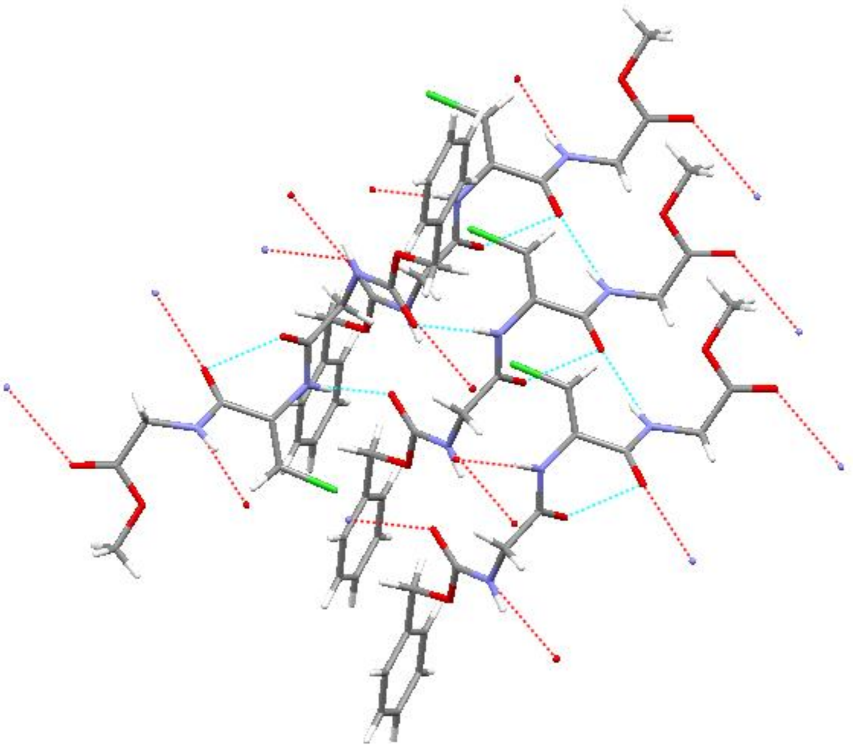

Figure 2S. Molecular interactions of the Cbz-Gly-(Z)-ΔAla(βCl)-Gly-OMe (**3**) molecules with visualization of the hydrogen bonds (dotted lines). Mercury 3.8 (Build RC2) (http://www.ccdc.cam.ac.uk/mercury/) was applied. The hydrogen bonds rely on the sum of van der Waals radii of the atom involved. The molecules with the conformation β (φ, ψ = -41.8°, 143.5°) are placed in parallel, N-terminus to N-terminus. Analogously, the molecules with the conformation -β (φ, ψ = 41.8°, -143.5°) are placed in parallel, but separately. The (Z)-ΔAla(βCl) residue, using the C-terminal amide group acting both as donor and acceptor of the N-H…O hydrogen bond, is connected with parallel residues preceding and following in this molecular ladder. However, the N-terminal amide group is the donor for the urethane group of the molecule with the opposite conformation.

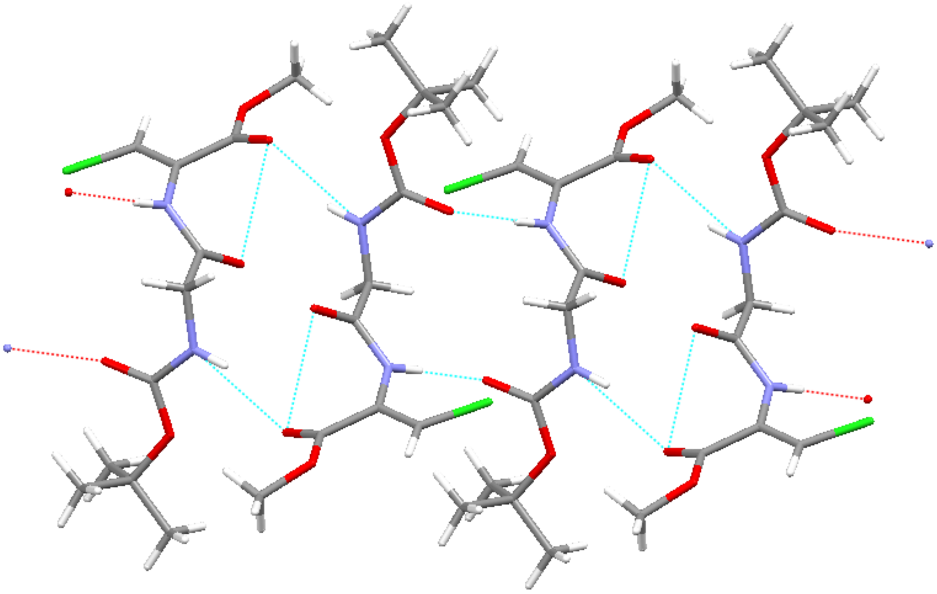

Figure 3S. Molecular interactions of the Boc-(Z)-ΔAla(βCl)-OMe (**4**) molecules with visualization of the hydrogen bonds (dotted lines). Mercury 3.8 (Build RC2) (http://www.ccdc.cam.ac.uk/mercury/) was applied. The hydrogen bonds rely on the sum of van der Waals radii of the atom involved. The molecules with the conformation β (φ, ψ = -56.5°, 168.6°) are placed antiparallel between the molecules with the conformation -β (φ, ψ = 56.5°, -168.6°) and each molecule creates four N-H…O hydrogen bonds, two by two with the preceding and following molecules in such arrangement. The N-terminal amide groups of the Gly and (Z)-ΔAla(βCl) residues are donors for the C=O groups of esters and urethanes of the neighboring molecules. Additionally, the C=O group of the C-terminal ester of the (Z)-ΔAla(βCl) residue is bonded with the N-H group of urethane of another molecule.

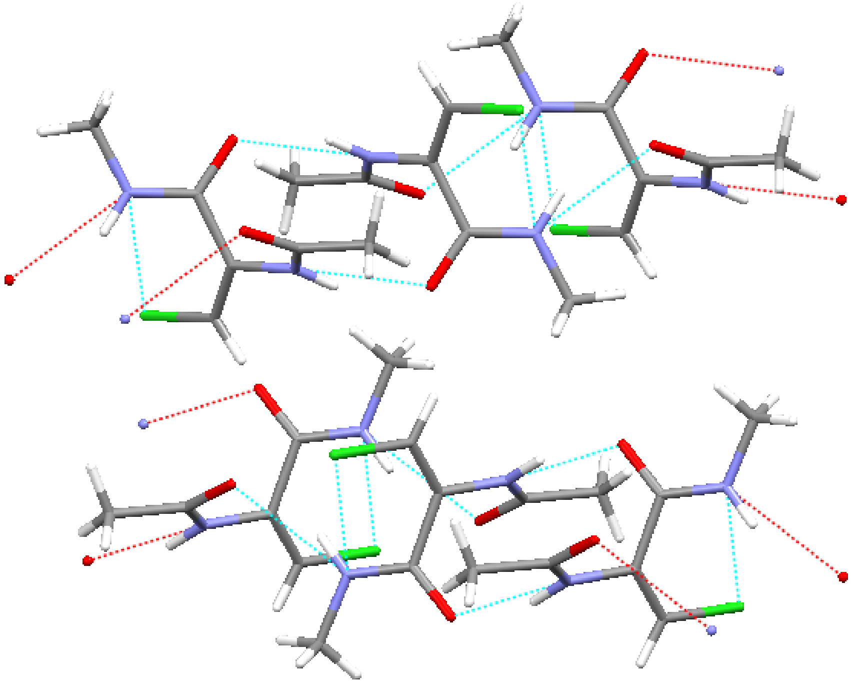

Figure 4S. Molecular interactions of the Ac-(*E*)-ΔAla(βCl)-NHMe (**2**) molecules with visualization of the hydrogen bonds (dotted lines). Mercury 3.8 (Build RC2) (http://www.ccdc.cam.ac.uk/mercury/) was applied. The hydrogen bonds rely on the sum of van der Waals radii of the atom involved. The molecules are in a linear arrangement. The molecules with the conformation β are placed with the molecules with the conformation -β alternately, with the (Z)-ΔAla(βCl) side chains upside-down and the N-terminus to C-terminus. Each molecule creates four N-H…O hydrogen bonds, two by two with the preceding and following molecules in an arrangement.

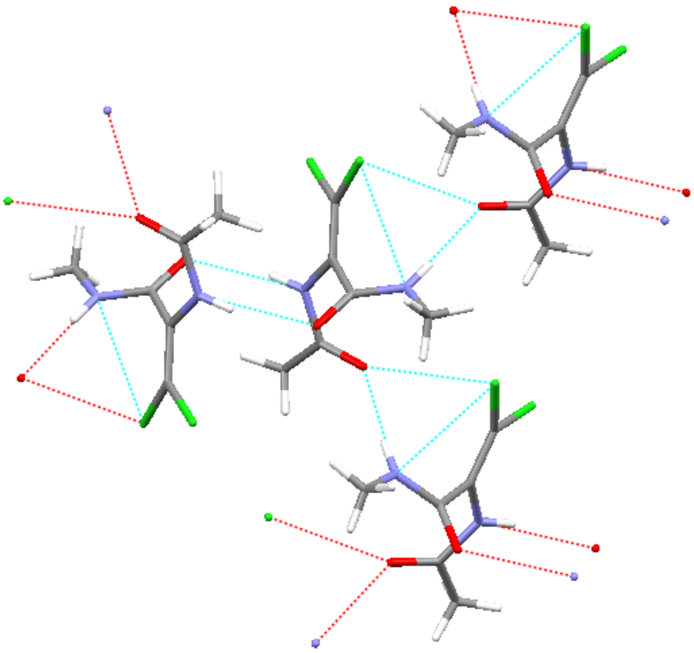

Figure 5S. Molecular interactions of the Ac-ΔAla(βCl_2)-NHMe (**5**) molecules with visualization of the hydrogen bonds (dotted lines). Mercury 3.8 (Build RC2) (http://www.ccdc.cam.ac.uk/mercury/) was applied. The hydrogen bonds rely on the sum of van der Waals radii of the atom involved. For Ac-ΔAla(βCl_2)-NHMe (**5**), the molecules with the opposite conformation, β and –β, and with the ΔAla(βCl_2) side chains upside-down, create centrosymmetric dimmers joined by two N-H…O hydrogen bonds. Additionally, each molecule is joined with two other molecules with the opposite conformations, creating the N-H…O hydrogen bond with each one.

Table 6S. Selected hydrogen-bond parameters (Å, º) of the studied compounds as determined by X-ray method.

**D—H···A**	**D—H (Å)**	**H···A (Å)**	**D···A (Å)**	**D—H···A (°)**
**Ac-(*Z*)-ΔAla(βCl)-NHMe (1)**				
C3'—H3'···O2'^{xv}	0.95	2.57	3.142 (3)	119.1
C5—H5B···O2^{xvi}	0.98	2.65	3.560 (3)	154.1
C5'—H5'C···O2^{xvii}	0.98	2.50	3.383 (3)	149.3
C5'—H5'B···O2	0.98	2.61	3.468 (3)	145.6
C6—H6A···Cl1^{xviii}	0.98	2.90	3.454 (3)	116.6
C6'—H6'B···Cl1^{xix}	0.98	2.97	3.909 (3)	161.7
N1—H1···O1'	0.92 (3)	2.01 (3)	2.923 (3)	170 (3)
N1'—H1'···O2'^{xv}	0.83 (3)	2.04 (3)	2.856 (3)	166 (3)
N2—H2···O1^{xvi}	0.90 (3)	1.92 (3)	2.791 (2)	161 (3)
N2'—H2'···O2	0.82 (3)	2.05 (3)	2.861 (2)	173 (3)
**Boc-(*Z*)-ΔAla(βCl)-OMe (4)**				
N3—H3A···O1^{i}	0.814 (16)	2.244 (16)	3.0211 (14)	159.7 (15)
C9—H9A···O4	0.98	2.53	3.0549 (15)	113.8
C8—H8C···O4	0.98	2.44	3.0230 (16)	118.1
C3—H3···O2^{ii}	0.95	2.39	3.1164 (15)	133.1
C5—H5A···O2^{i}	0.99	2.54	3.1962 (14)	123.5
N2—H2···O4^{iii}	0.842 (17)	2.037 (18)	2.8770 (13)	174.7 (16)
**Cbz-Gly-(*Z*)-ΔAla(βCl)-Gly-OMe (3)**				
N3—H3A···O5^{iii}	0.88	2.09	2.909 (3)	155.4
C5—H5B···O3^{iv}	0.99	2.62	3.437 (4)	139.9
C7—H7A···Cl1^{v}	0.99	2.89	3.548 (3)	124.6
C14—H14B···O2^{iii}	0.99	2.53	3.261 (4)	130.9
C14—H14A···O2^{i}	0.99	2.38	3.066 (4)	126.0
N1—H1···O1^{vi}	0.88 (4)	2.05 (4)	2.919 (3)	168 (3)
N2—H2···O3^{iv}	0.87 (4)	2.09 (4)	2.952 (3)	169 (3)
**Ac-ΔAla(βCl_2)-NHMe (5)**				
N1—H1···O2^{vii}	0.88	2.04	2.9122 (18)	172.2
N2—H2···O1^{i}	0.85 (2)	2.07 (2)	2.8958 (18)	163 (2)
C5—H5A···Cl2^{viii}	0.98	2.81	3.7197 (18)	154.4
C5—H5B···O1^{ix}	0.98	2.32	3.224 (2)	152.7
**Ac-(*E*)-ΔAla(βCl)-NHMe (2)**				
N1—H1···O2^{i}	0.88	2.09	2.914 (8)	154.9
N1'—H1'···O2'^{x}	0.88	2.03	2.821 (8)	148.6
N2—H2···O1^{xi}	0.88	2.01	2.863 (8)	162.9
N2'—H2'···O1'^{xii}	0.88	2.03	2.866 (8)	159.0
C3—H3···O1'	0.95	2.34	3.149 (9)	142.9
C3'—H3'···O2^{xiii}	0.95	2.54	3.289 (10)	135.8
C5—H5C···O1^{xi}	0.98	2.64	3.486 (10)	145.0
C5—H5B···O2^{ii}	0.98	2.54	3.495 (10)	164.1
C5'—H5B'···O1'^{xii}	0.98	2.60	3.443 (10)	143.8
C5'—H5C'···O2'^{xiv}	0.98	2.54	3.512 (10)	172.3
Symmetry code(s): (i) −*x*+1, −*y*+1, −*z*+1; (ii) −*x*+1, −*y*, −*z*+1; (iii) −*x*, −*y*+1, −*z*+1; (iv) −*x*, −*y*+2, −*z*+1; (v) −*x*+1, −*y*+2, −*z*+1; (vi) *x*+1, *y*, *z*; (vii) *x*, −*y*+3/2, *z*−1/2; (viii) *x*, *y*, *z*+1; (ix) *x*−1, *y*, *z*; (x) −*x*+1, −*y*, −*z*; (xi) −*x*+2, −*y*+1, −*z*+1; (xii) −*x*+1, −*y*+1, −*z*; (xiii) *x*, *y*, *z*−1; (xiv) −*x*, −*y*, −*z*; (xv) *x*, −*y*+1/2, *z*−1/2; (xvi) *x*, −*y*+3/2, *z*−1/2; (xvii) −*x*+1, −*y*+1, −*z*+1; (xviii) *x*, *y*, *z*+1; (xix) *x*, −*y*+1/2, *z*+1/2.				

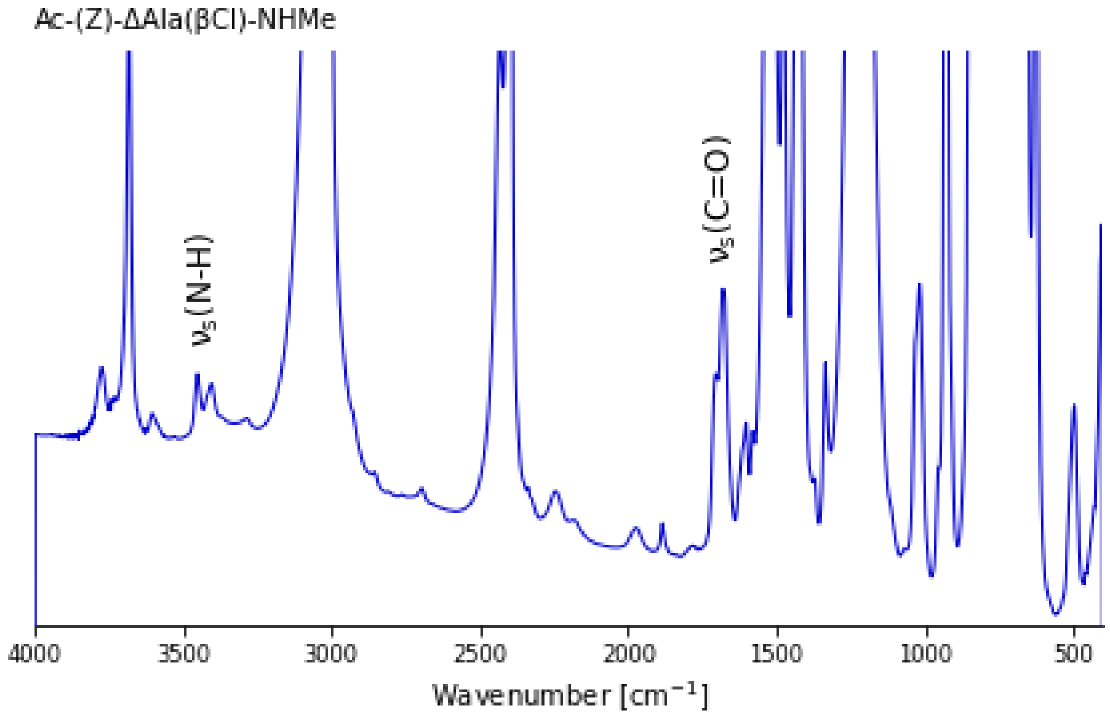

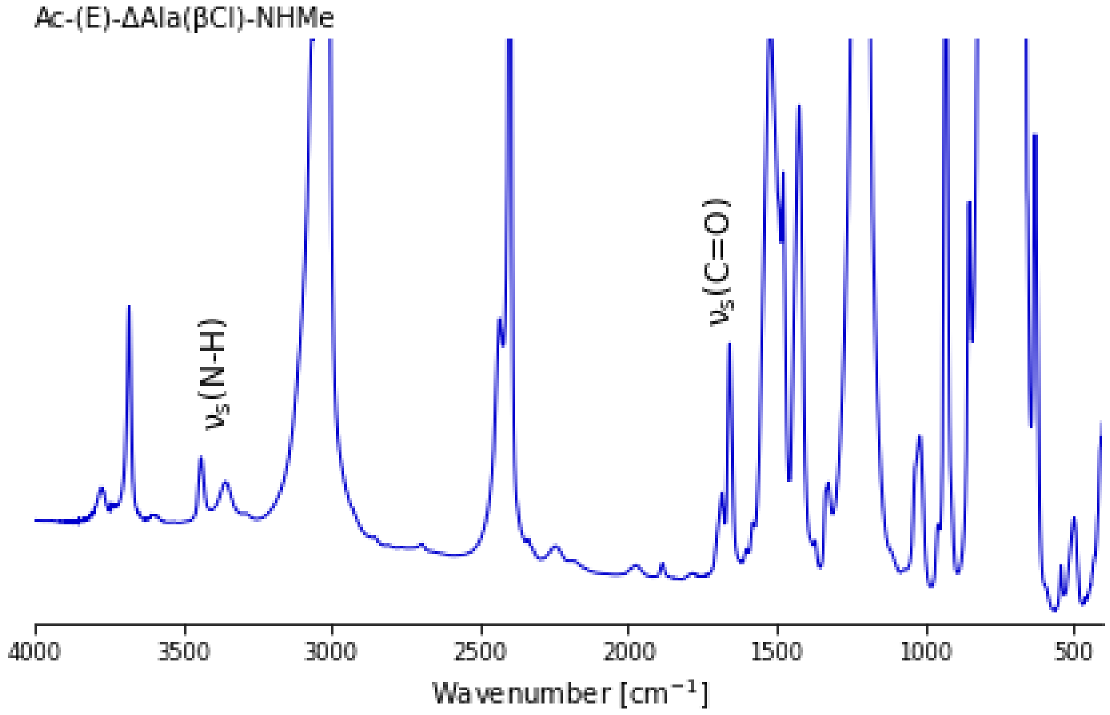

Figure 6S. FTIR spectra for the Ac-(*Z*)-ΔAla(βCl)-NHMe (**1**) and Ac-(*E*)-ΔAla(βCl)-NHMe (**2**) in $CHCl_3$.

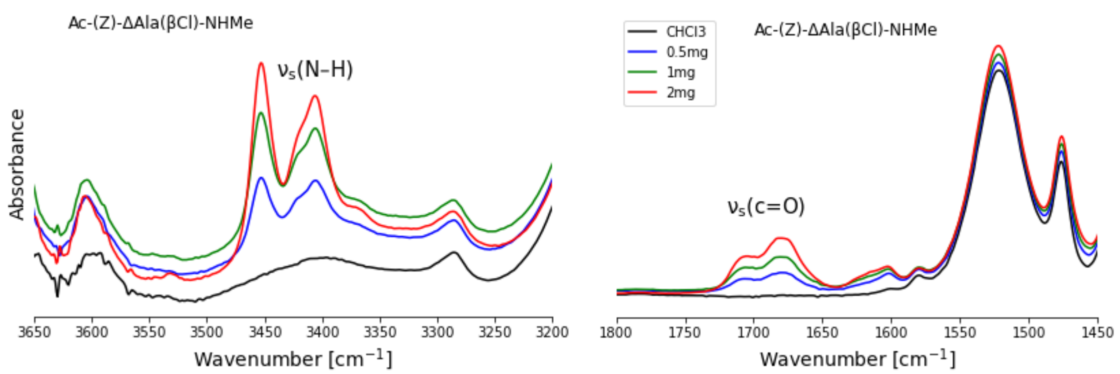

a)

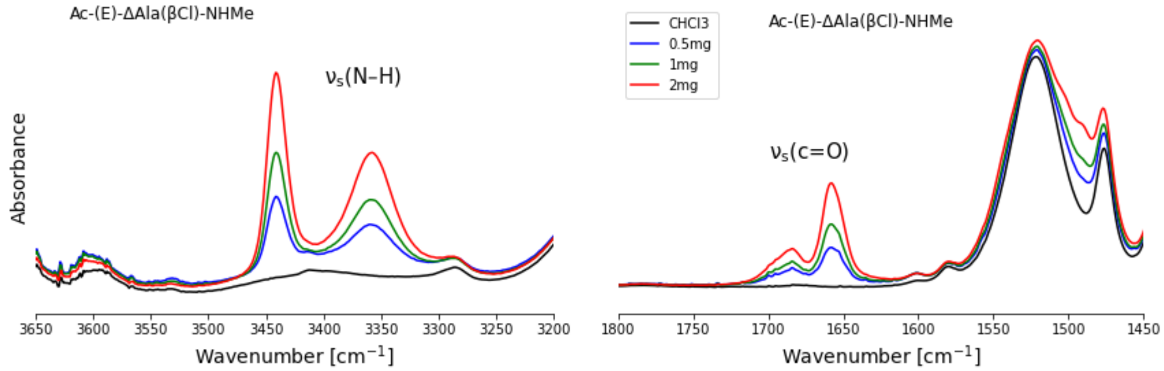

b)

Figure 7S. FTIR spectra for the Ac-(*Z*)-ΔAla(βCl)-NHMe (**1**) and Ac-(*E*)-ΔAla(βCl)-NHMe (**2**) in $CHCl_3$, region ν_s(N–H) and ν_s(C=O): a) isomer *Z*, b) isomer *E*.

Table 7S. Theoretical frequencies obtained by M06-2X/6-311+G(d,p) method for the conformations of the Ac-(*Z*)-ΔAla(βCl)-NHMe (**1**) and Ac-(*E*)-ΔAla(βCl)-NHMe (**2**) in chloroform (CPCM).

Conformation			Frequencies						RMS	
Code	φ (°)	ψ (°)	Experimental		Calculated		Scaled (0.936)		I	II
			C-terminal	N-terminal	C-terminal	N-terminal	C-terminal	N-terminal		
Ac-(*Z*)-ΔAla(βCl)-NHMe (**1**)			3452	3405						
			3422	3377						
α	-61.8	-23.1			3675	3615	3440	3384	14.0	20.0
β	-42.7	138.7			3667	3636	3432	3403	17.4	13.4
β2	-116.6	9.8			3667	3626	3432	3394	16.0	14.0
C7	-68.2	16.6			3617	3621	3386	3389	48.3	27.2
C5	-127.7	155.1			3674	3603	3439	3372	24.8	12.4
C5’	-121.0	-157.5			3677	3622	3442	3390	12.8	16.7
Ac-(*E*)-ΔAla(βCl)-NHMe (**2**)			3441	3358						
C5	-177.9	165.1			3678	3584	3443	3355	2.6	
β	-44.4	132.3			3670	3651	3435	3417	42.2	
α	-54.5	-37.6			3669	3638	3434	3405	33.7	
C7	-77.7	63.1			3572	3642	3343	3409	77.8	
β2	-172.7	53.1			3655	3641	3421	3408	38.0	

Scaling factor was established by the best match of the spectra (**2**) with the conformation C5 (Table 2).
RMS - Root mean squared error between experimental and calculated (scaled) frequencies.

Synthesis

All reaction mixtures were mixed using a magnetic stirrer. The specific conditions are mentioned at the appropriate description of synthetic procedures. The solvents ethyl acetate (EtOAc), benzene, hexane (Hex), methanol (MeOH), *N*,*N*-dimethylformamide (DFM - extra dry, stored over molecular sieves) were p.a. grade and used without any further purification. Tetrahydrofuran (THF) was refluxed over sodium with benzophenone as an indicator, then distilled and stored over sodium wire. Dichloromethane (DCM) and ethyl acetate (EtOAc) were refluxed over P_2O_5 and distilled. All reactants were used as supplied (Merck Poland). The progress of the reaction was monitored by thin layer chromatography using aluminum SiO_2 plates with a fluorescent indicator (Merck). The chromatograms were additionally visualized using chlorine/*o*-tolidine method. The products were purified by means of flash column chromatography using 60H silica gel with particle size 0.040-0.063 mm (Merck). The NMR analyses were performed on the Bruker Ultrashield 400 spectrometer operating at 400 MHz for ^{1}H and 101 MHz for ^{13}C. Samples were prepared in deuterated d_6-DMSO (99.9% at. D) or deuterated d_1-chloroform (99.8% at. D) with TMS as the internal standard.

Boc-L-Ser-NHMe Boc-L-Ser-OMe (2.107 g, 9.61 mmol) was dissolved in methanol (20 ml) and solution of methylamine in water 41% (11.85 M) (3.2 ml, 50 mmol, 5 eq.) was added. Reaction mixture was stirred for 48 h in room temperature, and then concentrated under reduced pressure. The residue was dissolved in AcOEt (150 ml), then washed with mixture of 1M HCl and brine (1:1, 20 ml). Aqueous layer then extracted with AcOEt (3x10 ml). All organic fractions were combined and dried over anhydrous magnesium sulfate. Product was crystallized from a mixture of AcOEt/Hex. Yeld: 1.378 g (6.32 mmol), 66%.

^{1}H NMR (400 MHz, $CDCl_3$) δ (ppm): 6.93 (1H, s, O=C-N-H), 5.76 (1H, d, O-C(O)-N-H), 4.19 (1H, s, O-H), 4.03 (1H, d, C^{α}-H), 3.83 (1H, s, CH_2), 3.68 (1H, s, CH_2), 2.83 (3H, d, CH_3), 1.45 (9H, s, $C(CH_3)_3$). ^{13}C NMR (101 MHz, $CDCl_3$) δ (ppm): 171.45 (O=C-N-H), 155.80 (O-C(O)-N-H), 80.06 $(C)^{Boc}$, 62.54 (C^{β}), 54.76 (C^{α}), 27.87 $(CH_3)^{Boc}$, 25.82 (CH_3) (Figure 1S). Melting point: 129.0-130.8°C. TLC MeOH/AcOH (1:9) R_f = 0.30, MeOH/DCM (1:9) R_f = 0.34.

Boc-L-Ser(OMs)-NHMe Boc-L-Ser-NHMe (0.2187 g, 1.00 mmol) was dissolved in anhydrous DCM (15 ml) and cooled in ice bath to -10°C. Afterwards 4-dimethylaminopyridine (DMAP) (0.0065 g, 0.053 mmol), triethylamine (0.418 ml, 3.00 mmol) and methanesulfonyl chloride (0.155 ml, 2.00 mmol) were added. Reaction solution was mixed for 80 min and warmed to room temperature. The reaction mixture was diluted with EtOAc (45 ml), washed with saturated solution of ammonium chloride (3x10 ml) and brine (10 ml) and dried with anhydrous magnesium sulfate. Product was purified by column chromatography using an increasing gradient EtOAc in Hex from 50% to 100% as eluent. Yield: 0.2630 g (0.8875 mmol), 89%.

^{1}H NMR (400 MHz, DMSO-d_6) δ (ppm): 8.02 (1H, d, O=C-N-H), 7.18 (1H, d, O-C(O)-N-H), 4.33 (1H, k, CH_2), 4.28 (1H, k, C^{α}-H), 4.23 (1H, k, CH_2), 3.16 (3H, s, $(CH_3)^{Ms}$), 2.60 (3H, d, CH_3), 1.39 (9H, s, $C(CH_3)_3$). ^{13}C NMR (101 MHz, DMSO-d_6) δ (ppm): 168.46 (O=C-N-H), 155.22 (O-C(O)-N-H), 78.64 $(C)^{Boc}$, 69.38 (C^{β}), 53.38 (C^{α}), 36.68 $(CH_3)^{Ms}$, 28.15 $(CH_3)^{Boc}$, 25.79 (CH_3) (Figure 2S). Melting point: 131.7-132.6°C. TLC MeOH/AcOH (1:19) R_f = 0.42.

Boc-ΔAla-NHMe Boc-L-Ser(OMs)-NHMe (0.1403 g, 0.4734 mmol) was dissolved in DCM (8 ml) and DBU (0.08 ml, 0.5 mmol) was added. Reaction mixture was stirred for 2 h in room temperature. The reaction mixture was diluted with Hex (8 ml), and then the products were directly purified by column chromatography using an increasing gradient EtOAc in Hex from 50% to 100% as eluent. Desired product was crystallized from a mixture of AcOEt/Hex. Yield: 0.0086 (0.043 mmol), 9%.

^{1}H NMR (400 MHz, DMSO-d_6) δ (ppm): 8.31 (1H, d, O=C-N-H), 7.89 (1H, s, O-C(O)-N-H), 5.59 (1H, s, (*E*)-C^{β}-H), 5.23 (1H, s, (*Z*)-C^{β}-H), 2.66 (3H, d, CH_3), 1.40 (9H, s, $C(CH_3)_3$). ^{13}C NMR (101 MHz,

DMSO-d_6) δ (ppm): 164.06 (O=C-N-H), 152.29 (O-C(O)-N-H), 135.75 (C^{α}), 99.42 (C^{β}), 79.51 $(C)^{Boc}$, 27.90 $(CH_3)^{Boc}$, 26.16 (CH_3) (Figure 3S). Melting point: 119-120°C. TLC MeOH/AcOH (1:19) R_f = 0.58. The cyclic side product was obtained in yield 0.0059 g (0.047 mmol), 10%. ^{1}H NMR (400 MHz, $CDCl_3$) δ (ppm): 8.26 (1H, s, N-H), 5.47 (1H, d, (*E*)-C^{β}-H), 4.97 (1H, d, (*Z*)-C^{β}-H), 3.10 (3H, s, CH_3). ^{13}C NMR (101 MHz, $CDCl_3$) δ (ppm): 162.64 $(O=C\text{-}N\text{-}H)^C$, 154.75 $(O=C\text{-}N\text{-}H)^N$, 133.67 ($C^{\alpha}$), 96.18 ($C^{\beta}$), 24.14 ($CH_3$) (Figure 4S). TLC MeOH/AcOH (1:19) R_f = 0.62. Unreacted substrate was recovered (0.0265 g, 0.0894 mmol, 19%).

Ac-ΔAla-OH Acetamide (11.81 g, 200 mmol), *p*-toluenesulphonic acid (3.804 g, 20 mmol, 0.1 eg.) and pyruvic acid (26.42 g, 300.0 mmol, 1.5 eq) in benzene (140 ml) were heated in Dean-Stark apparatus for 4 h. The crude product was filtered off and washed with diethyl ether and ethanol. The product was recrystallized from water and dried over phosphorus(V) oxide. Yield: 2.065 g (15.99 mmol), 7%.

^{1}H NMR (400 MHz, DMSO-d_6) δ (ppm): 13.28 (1H, s, O=C-O-H), 9.11 (1H, s, O=C-N-H), 6.25 (1H, s, (*E*)-C^{β}-H), 5.67 (1H, s, (*Z*)-C^{β}-H), 2.03 (3H, s, CH_3). ^{13}C NMR (101 MHz, DMSO-d_6) δ (ppm): 169.36 (O=C-O-H), 165.06 (O=C-N-H), 133.20 (C^{α}), 107.80 (C^{β}), 23.79 (CH_3) (Figure 5S). Melting point: 187.0-188.1°C. TLC chloroform/pyridine/AcOH (21:2:1) R_f = 0.42.

Ac-ΔAla-NHMe Ac-ΔAla-OH (0.258 g, 2.00 mmol) was dissolved in DMF (1 ml) then THF (1 ml) and *N*-methylmorpholine (0.231 ml, 2.1 mmol, 1.05 eq) were added. Solution was cooled in ice bath to -10°C, and isobutyl chloroformate (0.272 ml, 2.1 mmol, 1.05 eq) was added. After 10 min THF saturated with methylamine (3.7 ml, 3 mmol, 1.5 eq) was added dropwise. Reaction mixture was stirred for 3 h and warmed to room temperature. The volatile components were removed under reduced pressure and mixture was applied on column filed with ion exchange resin Dovex 2x8. The crude product was eluted with water then solvent was removed under reduced pressure. Finally, the product was purified by column chromatography using an increasing gradient of EtOAc in Hex from 50% to 100% as eluent, and crystallized from a mixture of EtOAc/Hex. Yield: 0.192 g (1.35 mmol), 34%.

^{1}H NMR (400 MHz, DMSO-d_6) δ (ppm): 9.05 (1H, s, $(O=C\text{-}N\text{-}H)^N$), 8.27 (1H, d, $(O=C\text{-}N\text{-}H)^C$), 5.99 (1H, s, (*E*)-$C^{\beta}$-H), 5.32 (1H, s, (*Z*)-$C^{\beta}$-H), 2.68 (3H, d, $(CH_3)^C$), 2.00 (3H, s, $(CH_3)^N$). ^{13}C NMR (101 MHz, DMSO-d_6) δ (ppm): 169.19 $(O=C\text{-}N\text{-}H)^N$, 164.48 $(O=C\text{-}N\text{-}H)^C$, 136.19 ($C^{\alpha}$), 102.31 ($C^{\beta}$), 26.10 $(CH_3)^C$, 23.90 $(CH_3)^N$ (Figure 6S). Melting point: 113.0-115.8°C. TLC MeOH/AcOEt (5:95) R_f = 0.33.

Ac-(Z)-ΔAla(βCl)-NHMe (***1***) Ac-ΔAla-NHMe (0.083 g, 0.58 mmol) was dissolved in DMF (0.25 ml) and dichloromethane (DCM) (2 ml) was added. Afterwards chlorine, dissolved in DCM, was added to the substrate solution until the light yellow color of the reaction mixture appear. Excess chlorine was removed quickly under reduced pressure and the residue was resuspended in DCM (2 ml). Finally, triethylamine (0.202 ml, 1.46 mmol, 2.5 eq.) was added and the reaction mixture was stirred for 15 min, and then, the solvents were evaporated under reduced pressure. Products **1** and **5** were isolated by column chromatography using an increasing gradient EtOAc in Hex from 50% to 100%, then an increasing gradient of MeOH in EtOAc 1-6%. Both products were finally crystallized from a mixture of AcOEt/Hex. The yield of **1** (main product): 0.054 g (0.31 mmol), 53%.

^{1}H NMR (400 MHz, DMSO-d_6) δ (ppm): 9.41 (1H, s, $(O=C\text{-}N\text{-}H)^N$), 8.05 (1H, d, $(O=C\text{-}N\text{-}H)^C$), 6.78 (1H, s, C^{β}-H), 2.62 (3H, d, $(CH_3)^C$), 1.96 (3H, s, $(CH_3)^N$). ^{13}C NMR (101 MHz, DMSO-d_6) δ (ppm): 168.54 $(O=C\text{-}N\text{-}H)^N$, 162.79 $(O=C\text{-}N\text{-}H)^C$, 134.32 ($C^{\alpha}$), 118.16 ($C^{\beta}$), 26.04 $(CH_3)^C$, 22.63 $(CH_3)^N$ (Figure 7S). Melting point: 170-171°C. TLC MeOH/AcOEt (7:93) R_f = 0.17. The yield of **5** (side product) 0.020 g (0.095 mmol), 16% ^{1}H NMR (400 MHz, DMSO-d_6) δ (ppm): 9.66 (1H, s, $(O=C\text{-}N\text{-}H)^N$), 8.32 (1H, d, $(O=C\text{-}N\text{-}H)^C$), 2.62 (3H, d, $(CH_3)^C$), 1.95 (3H, s, $(CH_3)^N$). ^{13}C NMR (101 MHz, DMSO-d_6) δ (ppm): 168.04 $(O=C\text{-}N\text{-}H)^N$, 161.69 $(O=C\text{-}N\text{-}H)^C$, 132.12 ($C^{\alpha}$), 114.72 ($C^{\beta}$), 25.94 $(CH_3)^C$, 22.45 $(CH_3)^N$ (Figure 12S). TLC MeOH/AcOEt (7:93) R_f = 0.37.

Cbz-Gly-(Z)-ΔAla(βCl)-Gly-OMe (**3**) The synthesis was performed according to the procedure described for compound **1** using Cbz-Gly-ΔAla-Gly-OMe (0.105 g, 0.301 mmol) as substrate. The product was purified by means of column chromatography using increasing gradient of EtOAc in Hex from 30% to 100% and crystallized from EtOAc/Hex mixture. Yield: 0.079 g (0.21 mmol), 69%.

^{1}H NMR (400 MHz, DMSO-d_6) δ (ppm): 9.58 (1H, d, (O=C-N-H)^{N}), 8.54 (1H, t, O=C-N-H)^{C}), 7.53 (1H, t, (O-C(O)-N-H), 7.39-7.29 (5H, m, (C-H)^{bzn}), 6.95 (1H, s, C^{β}-H), 5.04 (2H, s, (CH$_2$)^{bzl}), 3.86 (2H, d, (CH$_2$)^{Gly,N}), 3.83 (2H, d, (CH$_2$)$^{Gly-C}$), 3.63 (3H, s, CH$_3$). ^{13}C NMR (101 MHz, DMSO-d_6) δ (ppm): 169.99 (O-C=O), 168.36 (O=C-N-H)^{N}, 162.61 (O=C-N-H)^{C}, 156.55 (O-C(O)-NH), 137.04 (C^{bzn}), 132.99 (C^{α}), 128.39 (C^{bzn,m}-H), 127.85(C^{bzn,p} -H), 127.77 (C^{bzn,o}-H), 120.66 (C^{β}), 65.54 (CH$_2$)^{bzl}, 51.80 (CH$_3$), 43.39 (CH$_2$)^{Gly,N}, 40.97 (CH$_2$)^{Gly,C} (Figure 14S). Melting point: 144.2-144.9°C. TLC AcOEt R$_f$ = 0.17.

Boc-Gly-(Z)-ΔAla(βCl)-OMe (**4**) The synthesis was performed according to the procedure described for compound **1** using Boc-Gly-ΔAla-OMe (0.129 g, 0.441 mmol) as substrate. The product was purified by means of column chromatography using increasing gradient of EtOAc in Hex from 10% to 80% and crystallized from EtOAc/Hex. mixture. Yield: 0.104 g (0.355 mmol), 71%.

^{1}H NMR (400 MHz, DMSO-d_6) δ (ppm): 9.68 (1H, d, O=C-N-H), 7.15 (1H, d, C^{β}-H), 7.04 (1H, t, O-C(O)-N-H), 3.70 (2H, d, (CH$_2$)^{Gly}), 3.68 (3H, s, CH$_3$), 1.37 (9H, d, C(CH$_3$)$_3$). ^{13}C NMR (101 MHz, DMSO-d_6) δ (ppm): 168.76 (O=C-N-H), 162.76 (O=C-O), 155.81 (O-C(O)-N-H), 130.40 (C^{α}), 122.88 (C^{β}), 78.12 (C)^{Boc}, 52.48 (CH$_3$), 42.79 (CH$_2$)^{Gly}, 28.19 (CH$_3$)^{Boc} (Figure 19S). Melting point: 117.3-118.4°C. TLC Toluene/AcOEt (3:7) R$_f$ = 0.50, AcOEt/Hex (4:6) R$_f$= 0,13.

Ac-(E)-ΔAla(βCl)-NHMe (**2**) Ac-(Z)-ΔAla(βCl)-NHMe (**1**) (0.0367 g, 0.208 mmol) and benzophenone (0.1895 g, 1.040 mmol, 5.0 equiv) were dissolved in methanol (2 ml) and benzene (0.8 ml) was added. Reaction mixture was mixed and illuminated simultaneously with UV light (366 nm) for 5 h with intensity 400-440 μW/cm^2. The volatile components was coevaporated with DCM (15 ml), residue was adsorbed on silica gel and then applied on chromatographic collumn. An increasing gradients EtOAc in Hex from 50% to 100%, then MeOH in EtOAc 0.5%-5% were used as eluent. The product was crystallized from a mixture of AcOEt/Hex. Yield: 0.0074 g (0.042 mM), 20%. Unreacted substrate was recovered (0.0293 g, 0.166 mmol, 80%).

^{1}H NMR (400 MHz, DMSO-d_6) δ (ppm): 9.76 (1H, s, (O=C-N-H)^{N}), 8.33 (1H, d, (O=C-N-H)^{C}), 6.90 (1H, s, C^{β}-H), 2.66 (3H, d, (CH$_3$)^{C}), 1.94 (3H, s, (CH$_3$)^{N}). ^{13}C NMR (101 MHz, DMSO-d_6) δ (ppm): 168.95 (O=C-N-H)^{N}, 162.56 (O=C-N-H)^{C}, 135.35 (C^{α}), 105.78 (C^{β}), 25.69 (CH$_3$)^{C}, 23.13 (CH$_3$)^{N} (Figure 23S). Melting point: 130-131°C. TLC MeOH/AcOEt (6:94) R$_f$ = 0.33.

Cbz-Gly-(E)-ΔAla(βCl)-Gly-OMe Cbz-Gly-(*Z*)-ΔAla(βCl)-Gly-OMe (**3**) (0.0153 g, 0.0399 mmol) and benzophenone (0.0364 g, 0.200 mmol, 5.0 equiv) were dissolved in methanol (1 ml) and benzene (0.4 ml) was added. Then the photoisomerization reaction was carried out in the same manner as for compound **2**. The isomers were separated by column chromatography using an increasing gradient EtOAc in Hex from 50% to 100% as eluent. Yield: 0.0023 g (0.0060 mmol), 15%. Unreacted substrate was recovered (0.0125 g, 0.0326 mmol, 82%).

^{1}H NMR (400 MHz, DMSO-d_6) δ (ppm): 9.80 (1H, d, (O=C-N-H)^{N}), 8.81 (1H, t, (O=C-N-H)^{C}), 7.55 (1H, t, O-C(O)-N-H), 7.40-7.29 (5H, m, (C-H)^{bzn}), 6.98 (1H, s, C^{β}-H), 5.03 (2H, s, (CH$_2$)^{bzl}), 3.93 (2H, d, (CH$_2$)^{Gly,N}), 3.73 (2H, d, (CH$_2$)$^{Gly-C}$), 3.65 (3H, s, CH$_3$). ^{13}C NMR (101 MHz, DMSO-d_6) δ (ppm): 169.64 (O-C=O), 168.71 (O=C-N-H)^{N}, 162.31 (O=C-N-H)^{C}, 156.56 (O-C(O)-NH), 137.01 (C^{bzn}), 133.55 (C^{α}), 128.40 (C^{bzn,m}-H), 127.86(C^{bzn,p} -H), 127.77 (C^{bzn,o}-H), 108.54 (C^{β}), 65.54 (CH$_2$)^{bzl}, 51.78 (CH$_3$), 43.58 (CH$_2$)^{Gly,N}, 40.85 (CH$_2$)^{Gly,C} (Figure 28S). TLC AcOEt R$_f$ = 0.37.

Boc-Gly-(E)-ΔAla(βCl)-OMe The photoisomerization reaction of Boc-Gly-(*Z*)-ΔAla(βCl)-OMe (**4**) (0.0146 g, 0.0499 mmol) was carried out in the same manner as for compound **2**. The isomers were

separated by column chromatography using an increasing gradient EtOAc in Hex from 20% to 100% as eluent. Yield: 0.0041 g (0.014 mmol), 28%. Unreacted substrate was recovered (0.0099 g, 0.034 mmol, 68%).
^{1}H NMR (400 MHz, DMSO-*d$_6$*) δ (ppm): 10.02 (1H, d, O=C-N-H), 7.09 (1H, t, O-C(O)-N-H), 6.77 (1H, d, C^{β}-H), 3.72 (3H, d, CH_3), 3.63 (2H, d, $(CH_2)^{Gly}$), 1.38 (9H, d, $(CH_3)^{Boc}$). ^{13}C NMR (101 MHz, DMSO-*d$_6$*) δ (ppm): 168.65 (O=C-N-H), 162.59 (O=C-O), 155.84 (O-C(O)-N-H), 130.73 (C^{α}), 112.40 (C^{β}), 78.22 $(C)^{Boc}$, 52.29 (CH_3), 42.99 $(CH_2)^{Gly}$, 28.19 $(CH_3)^{Boc}$ (Figure 29S). TLC AcOEt/Hex (4:6) R_f = 0.23.

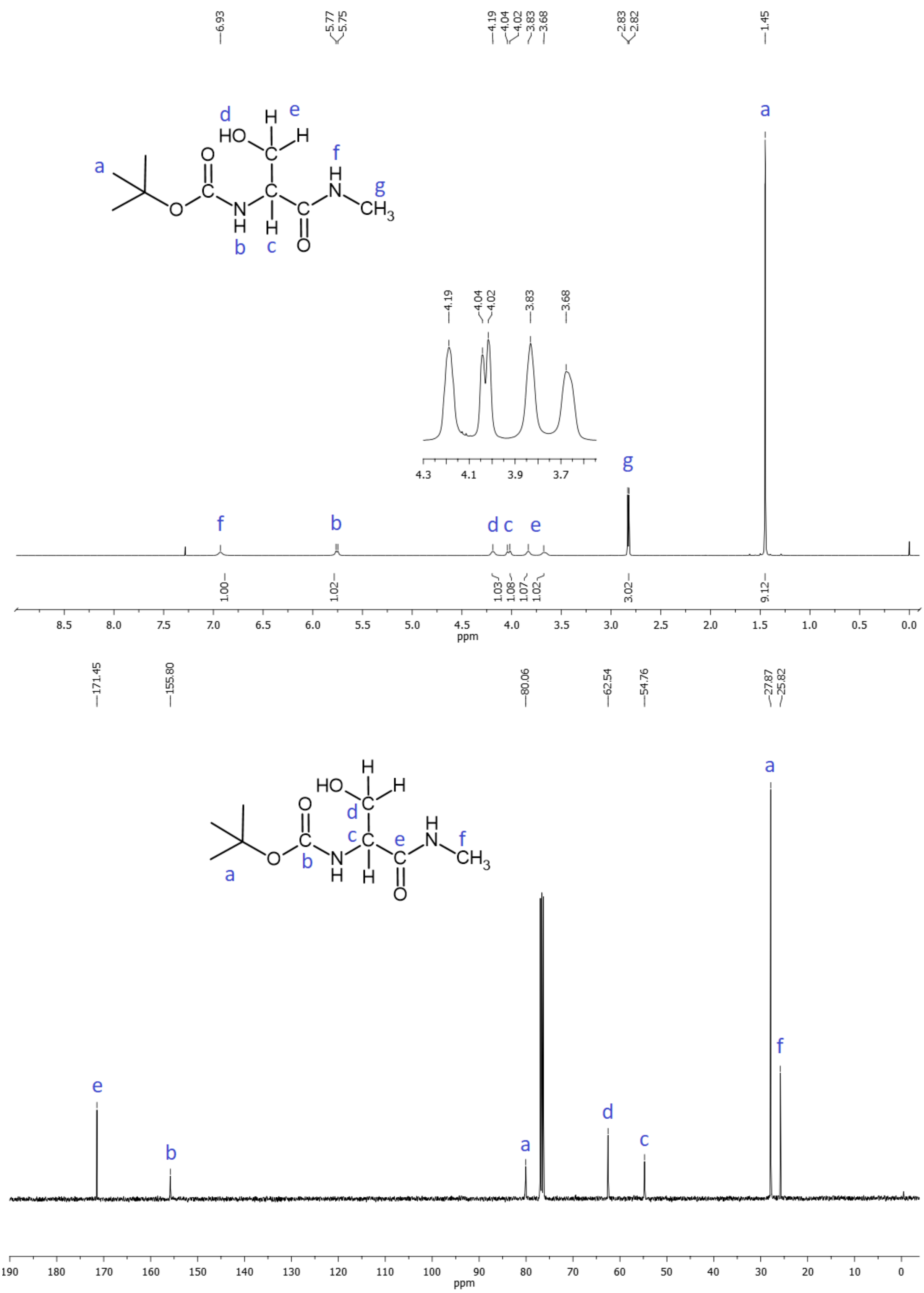

Figure 8S. ^{1}H and ^{13}C NMR spectra of Boc-L-Ser-NHMe in chloroform.

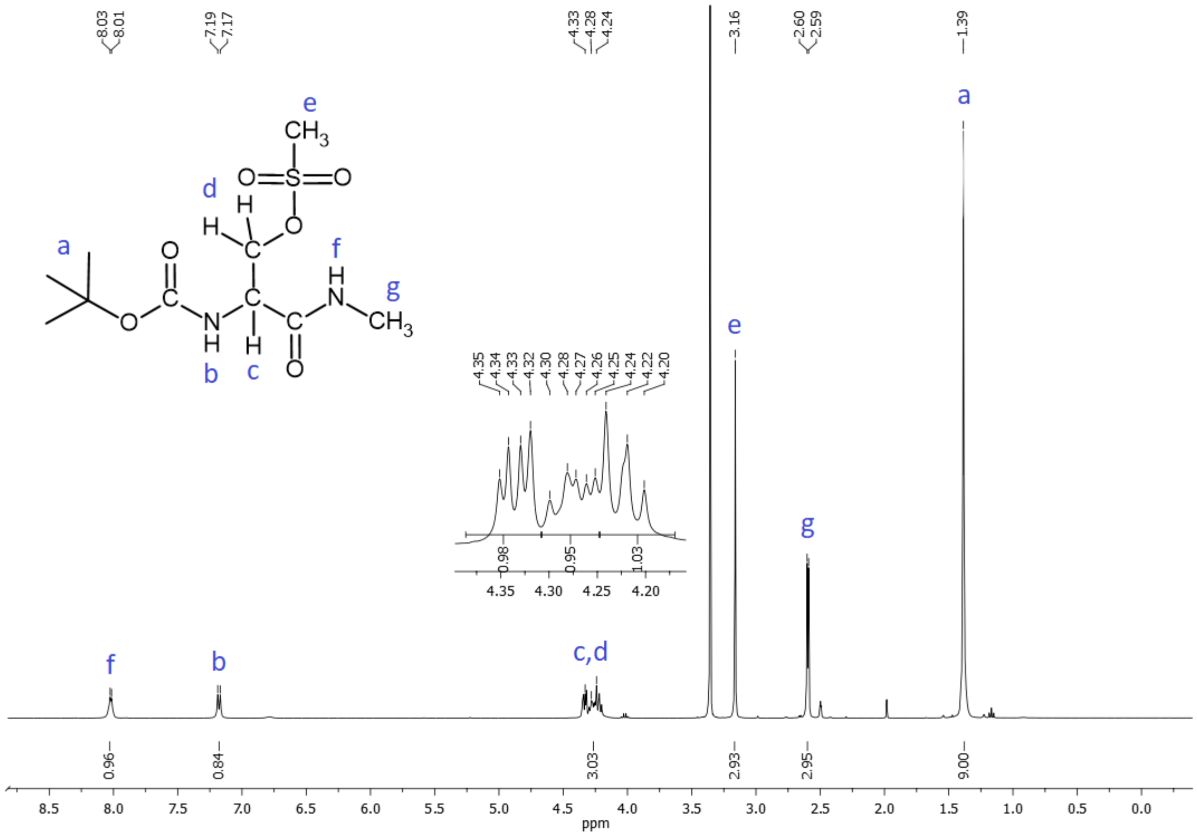

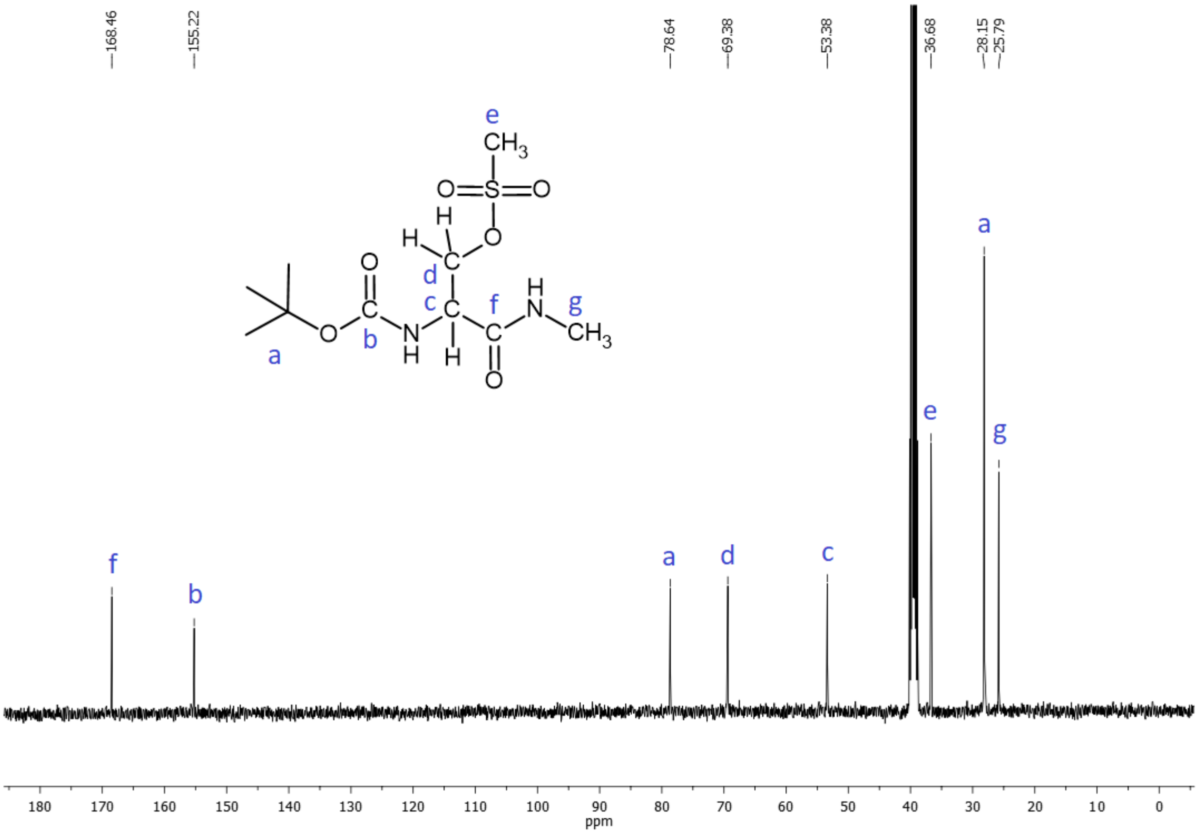

Figure 9S. ^{1}H and ^{13}C NMR spectra of Boc-L-Ser(OMs)-NHMe in DMSO-d_6.

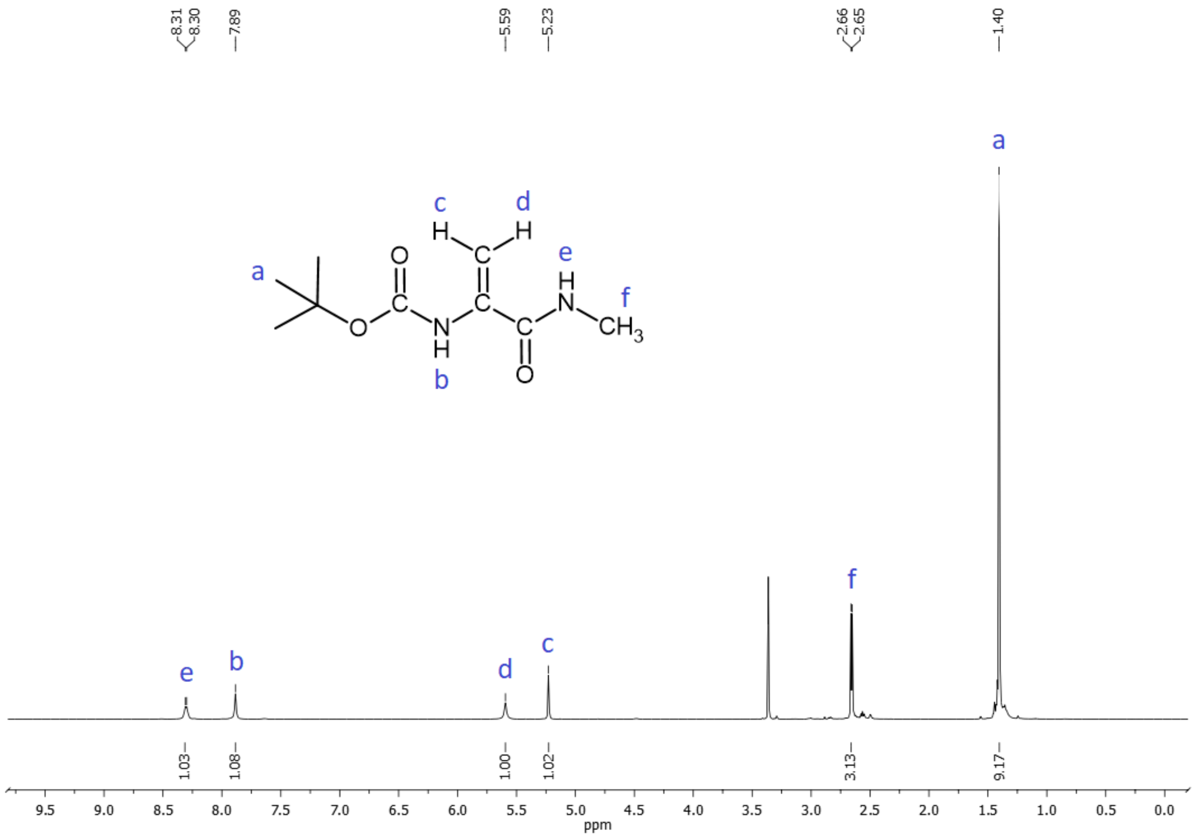

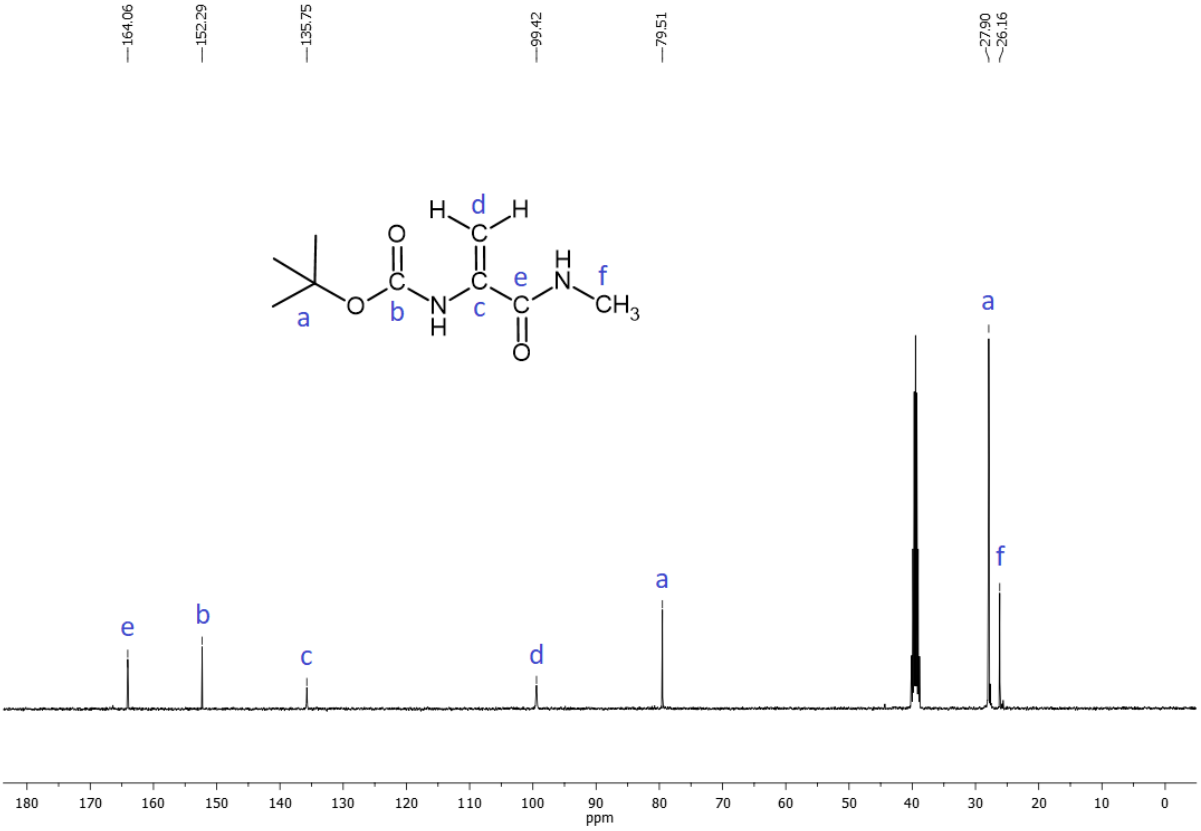

Figure 10S. ^{1}H and ^{13}C NMR spectra of Boc-ΔAla-NHMe in DMSO-d_6.

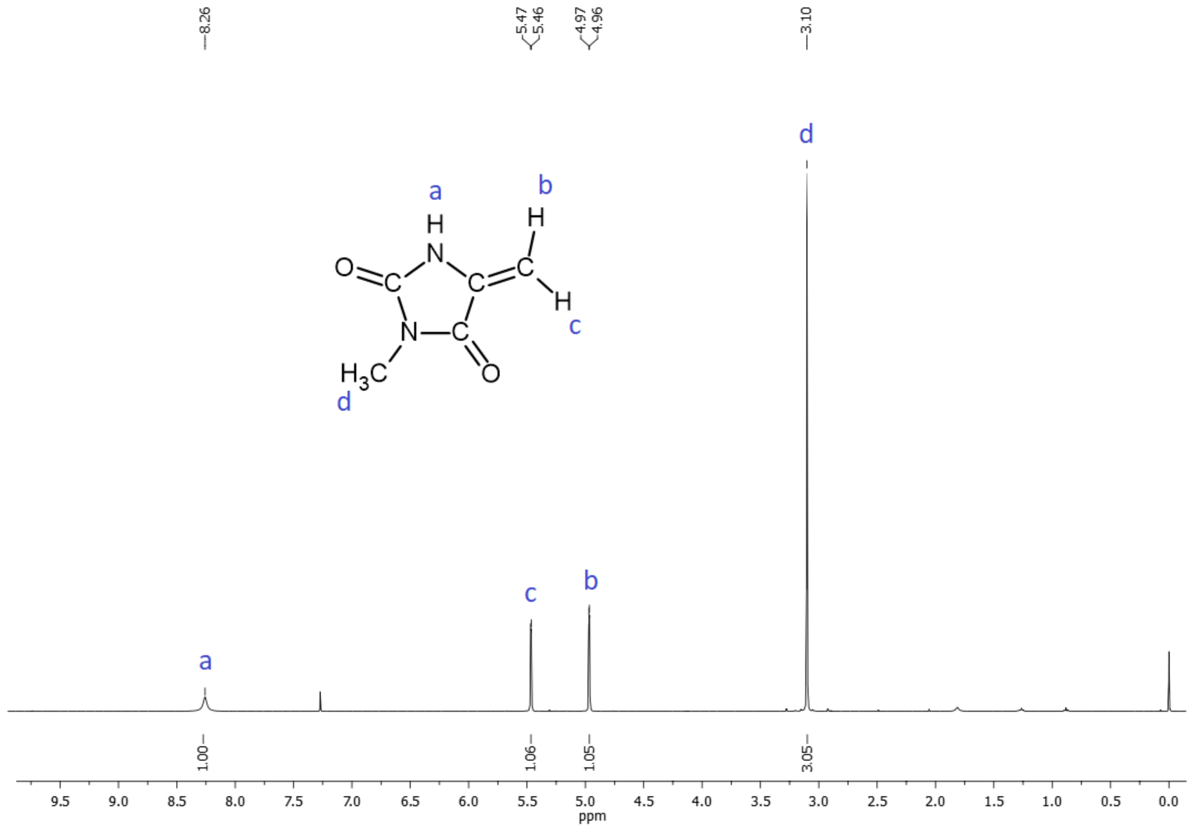

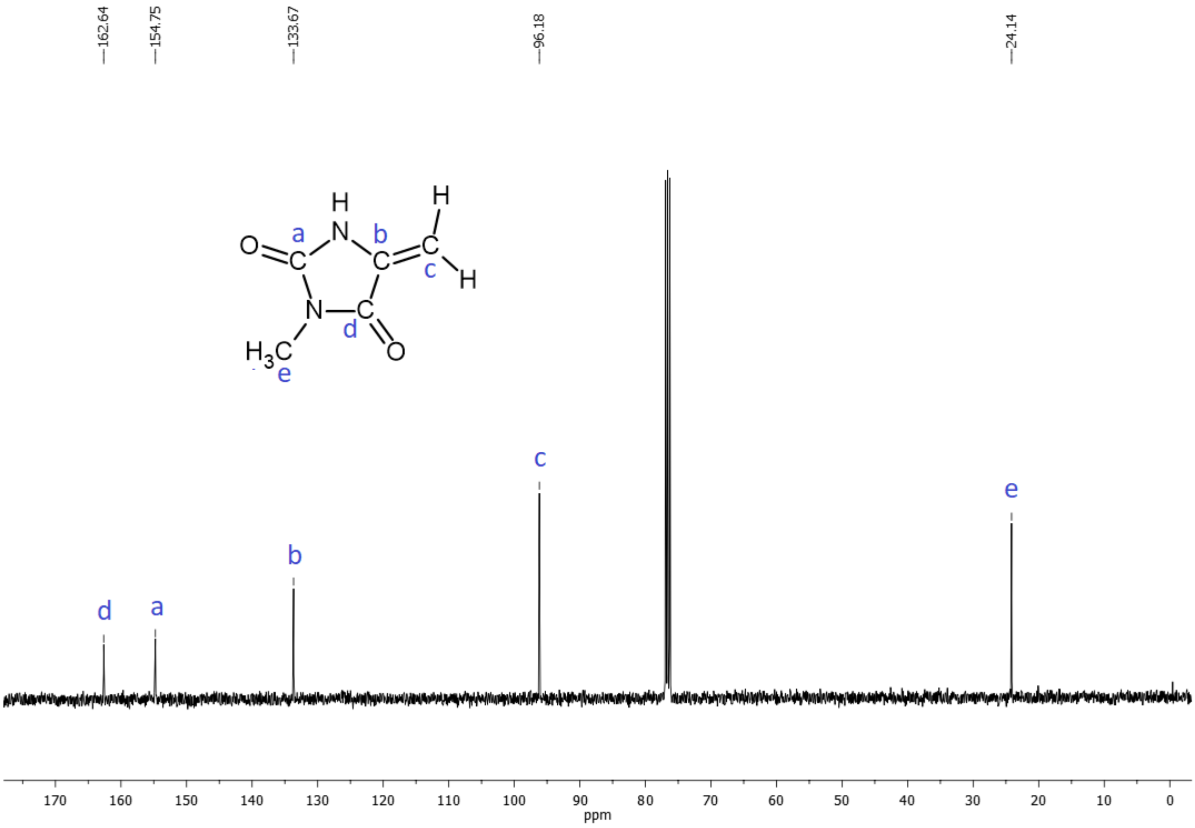

Figure 11S. ^{1}H and ^{13}C NMR spectra of cyclic side product Boc-ΔAla-NHMe synthesis in CD_3Cl.

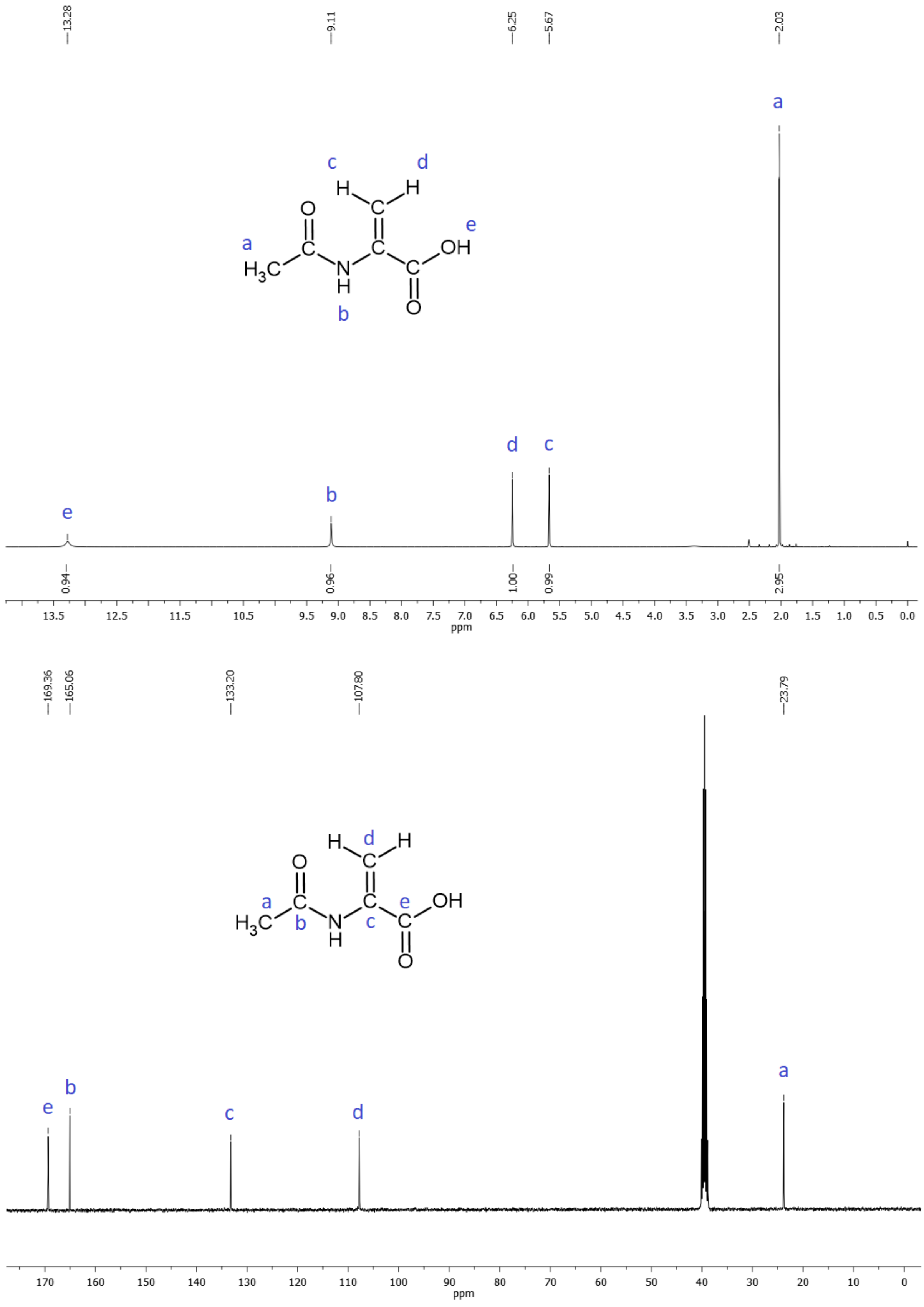

Figure 12S. ^{1}H and ^{13}C NMR spectra of Ac-ΔAla-OH in DMSO-d_6.

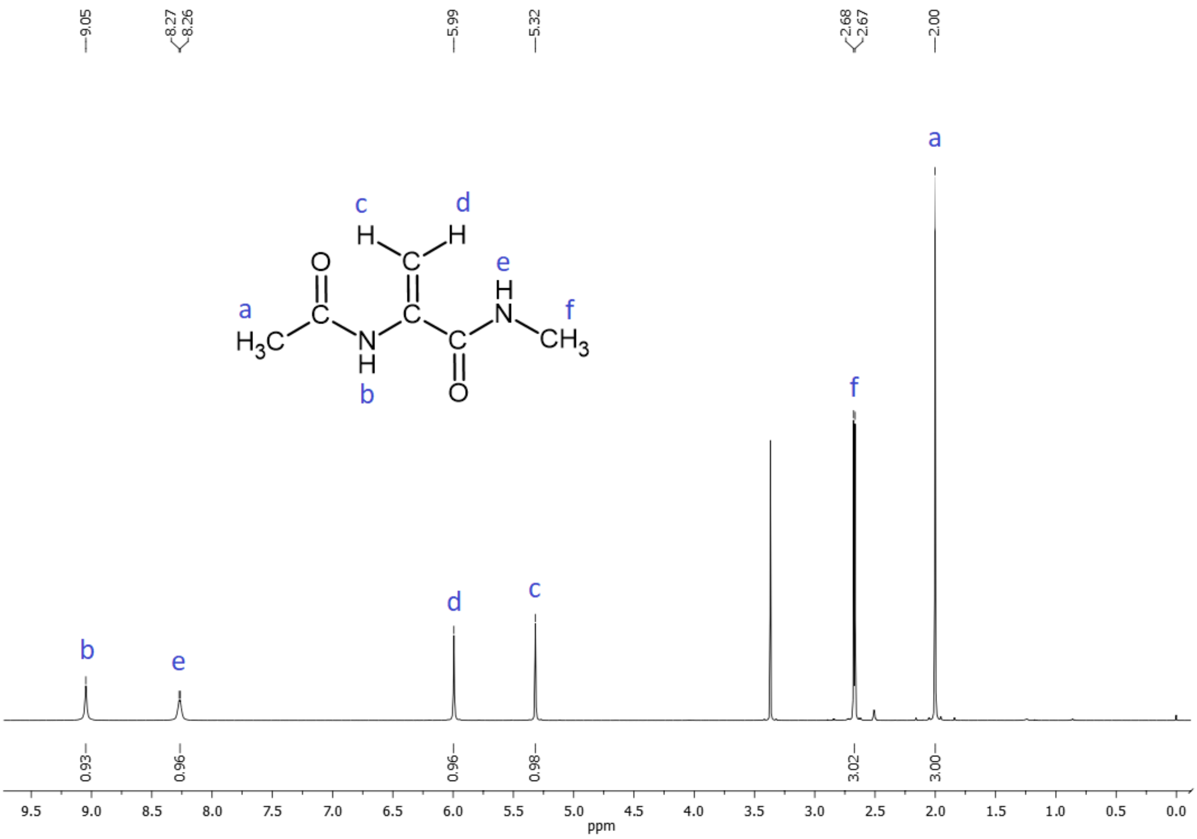

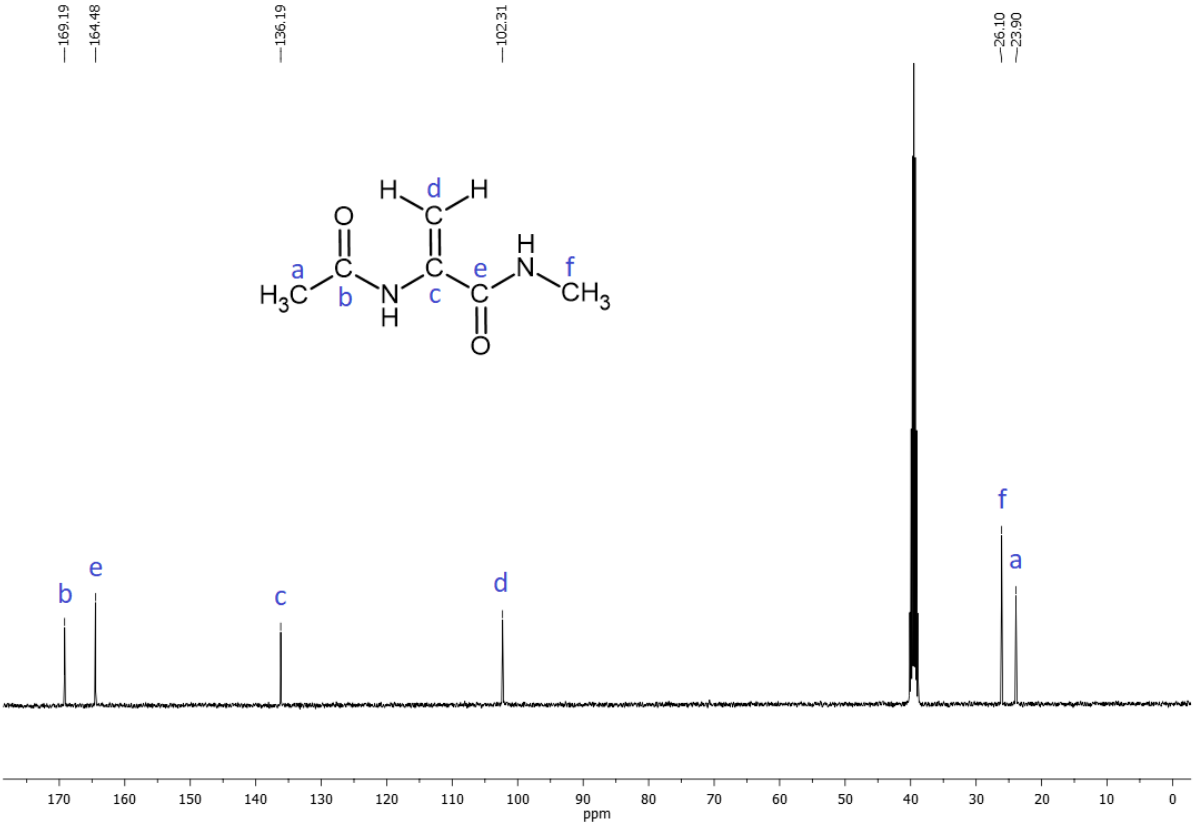

Figure 13S. ^{1}H and ^{13}C NMR spectra of Ac-ΔAla-NHMe in DMSO-d_6.

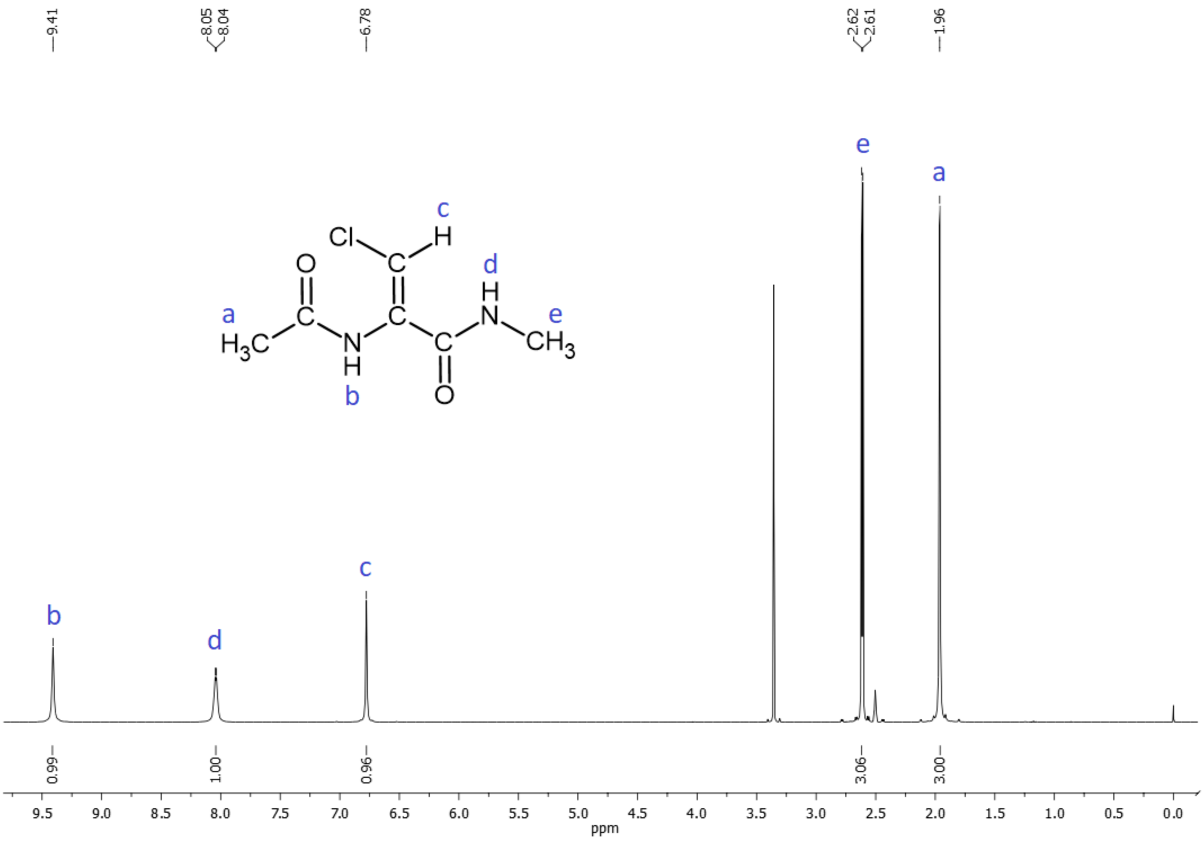

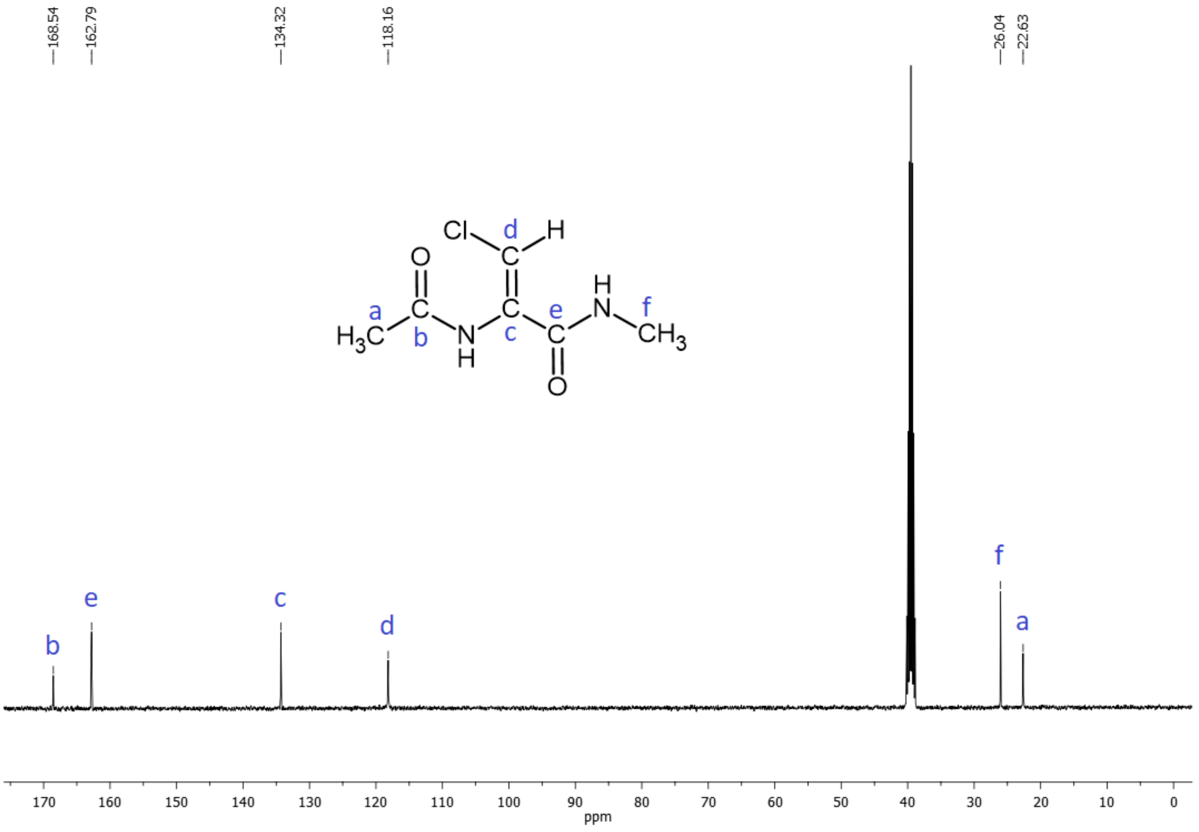

Figure 14S. ^{1}H and ^{13}C NMR spectra of Ac-(*Z*)-ΔAla(βCl)-NHMe (**1**) in DMSO-d_6.

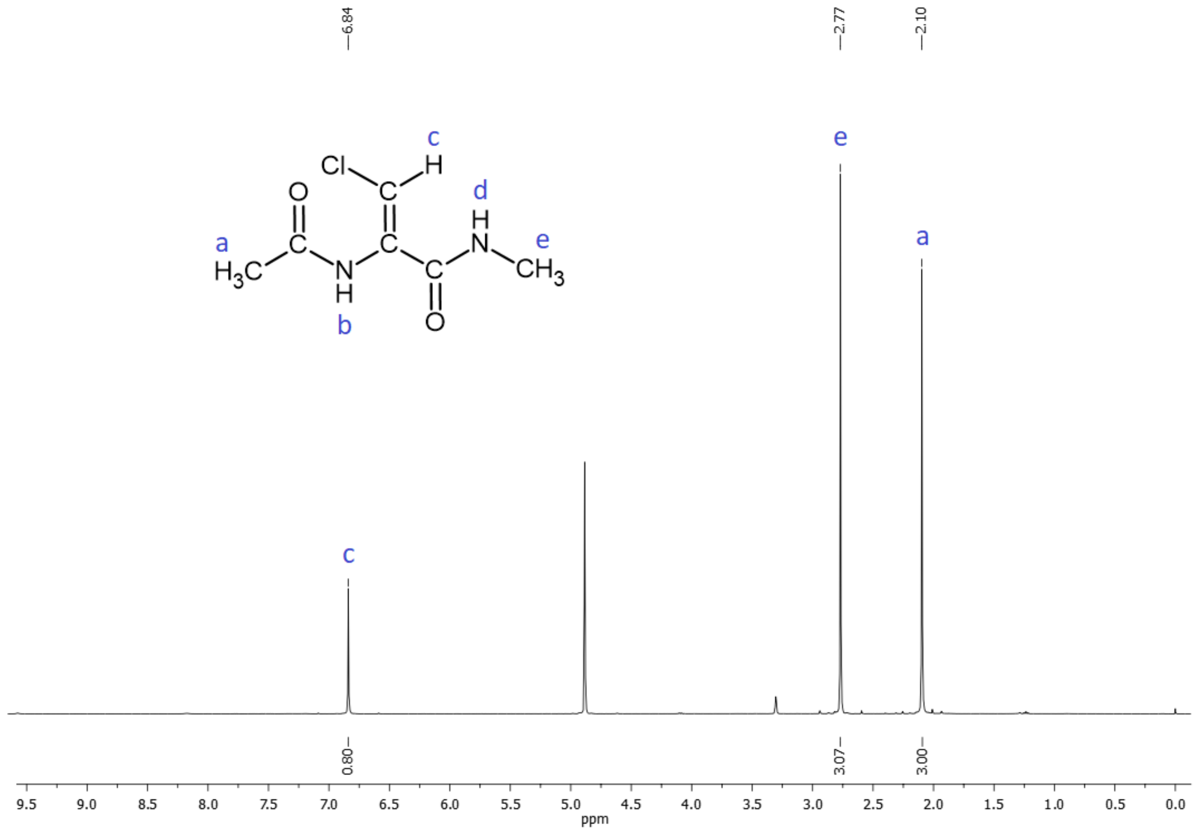

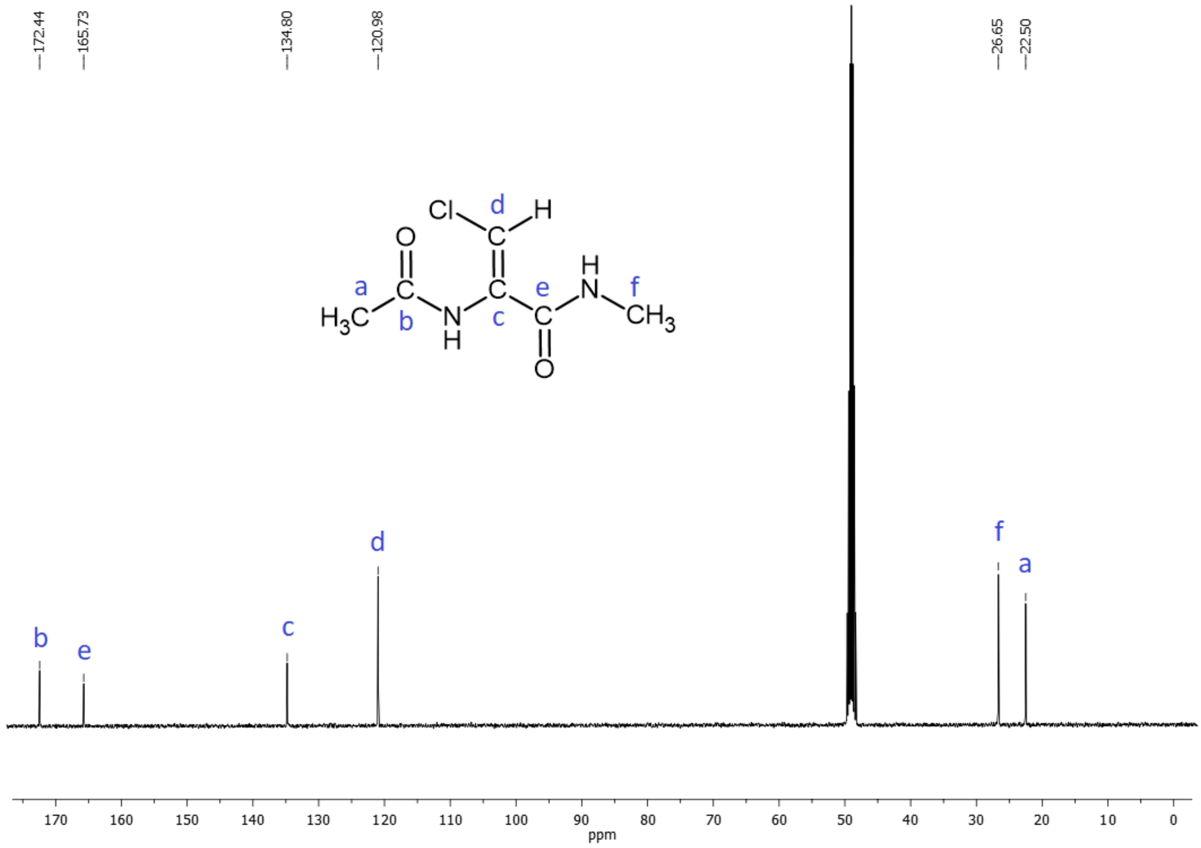

Figure 15S. ^{1}H and ^{13}C NMR spectra of Ac-(*Z*)-ΔAla(βCl)-NHMe (**1**) in CD_3OD.

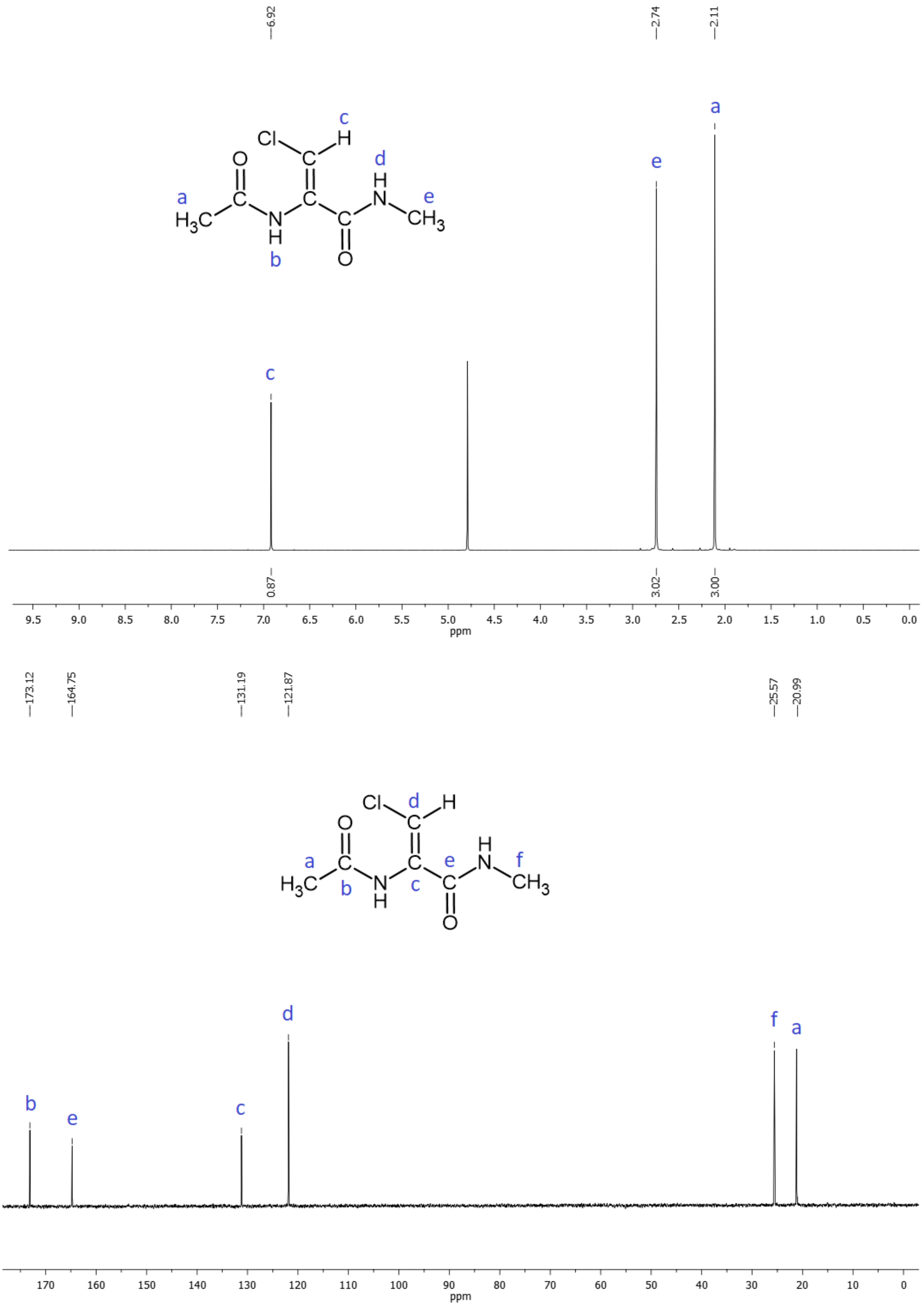

Figure 16S. ^{1}H and ^{13}C NMR spectra of Ac-(*Z*)-ΔAla(βCl)-NHMe (**1**) in D_2O.

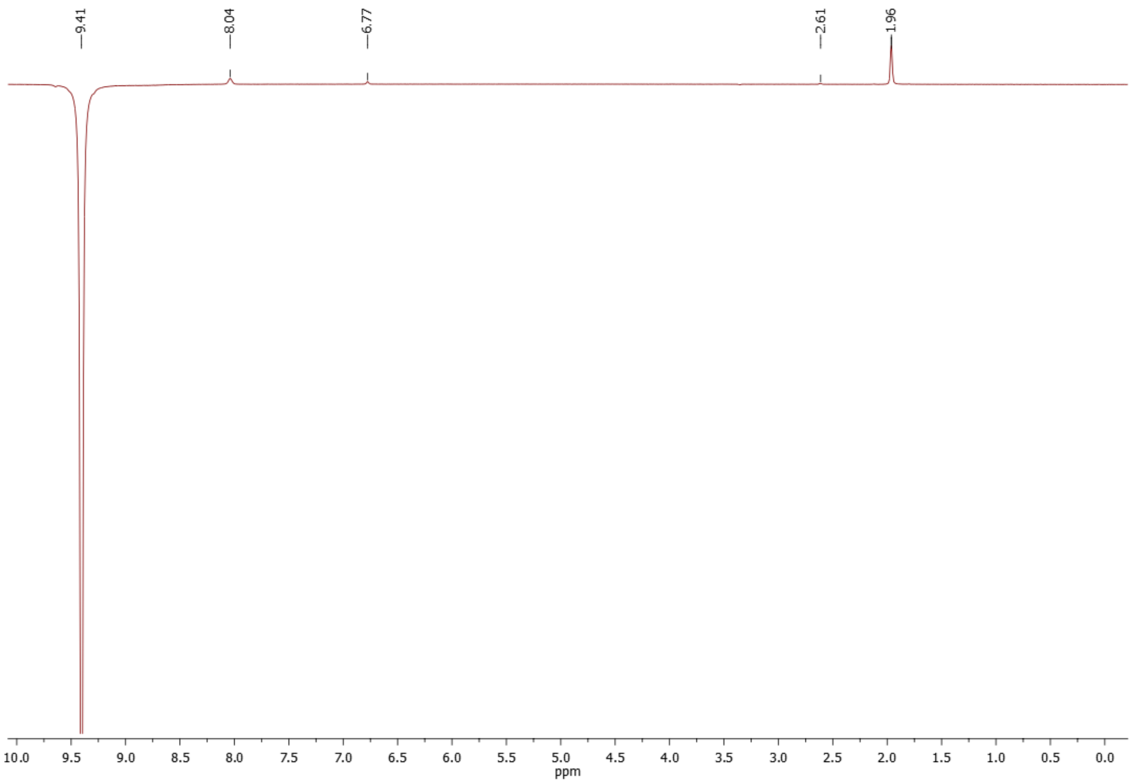

Figure 17S. ^{1}H NMR 1D-NOE spectra obtained by selective excitation of the N-terminal amide H atom of Ac-(*Z*)-ΔAla(βCl)-NHMe (**1**) in DMSO-d_6.

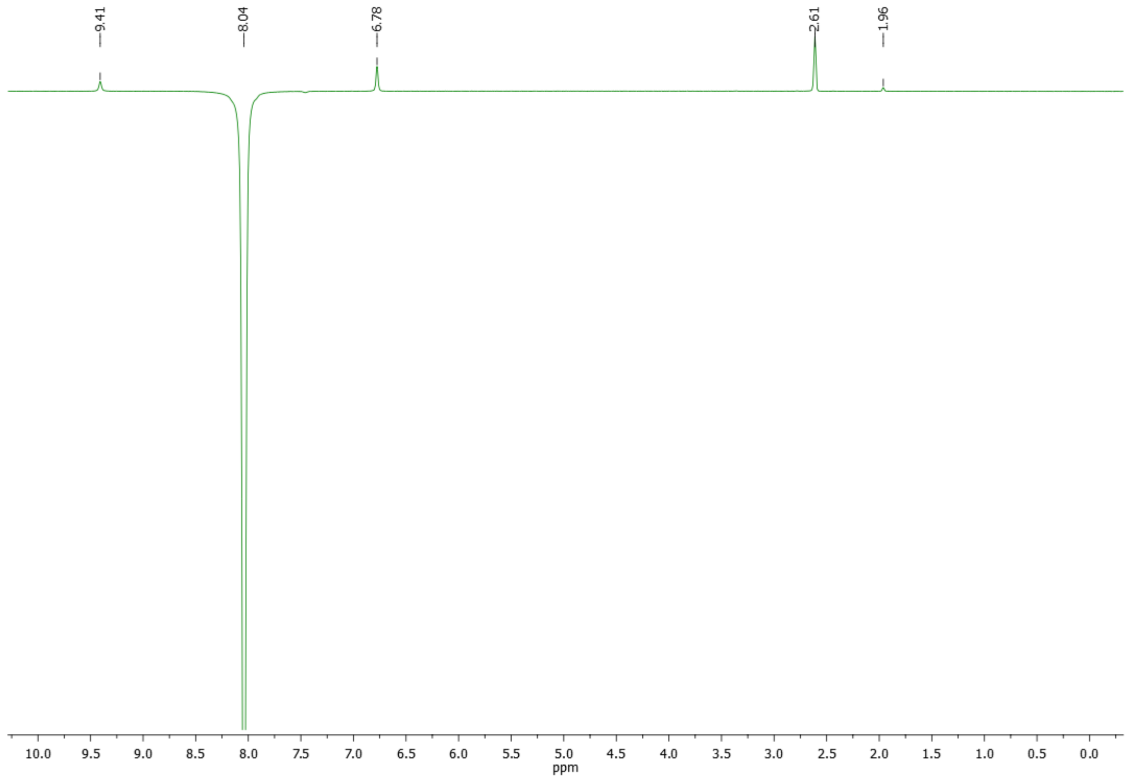

Figure 18S. ^{1}H NMR 1D-NOE spectra obtained by selective excitation of the C-terminal amide H atom of Ac-(*Z*)-ΔAla(βCl)-NHMe (**1**) in DMSO-d_6.

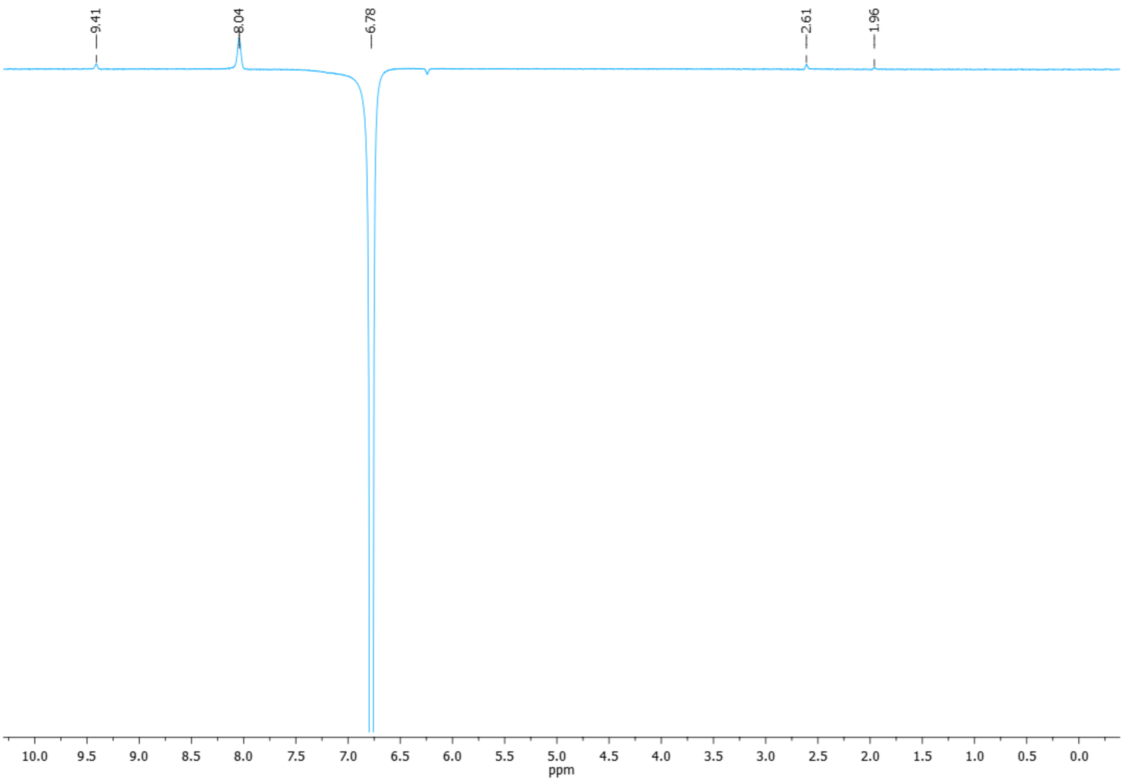

Figure 19S. ^{1}H NMR 1D-NOE spectra obtained by selective excitation of the side chain H atom of Ac-(*Z*)-ΔAla(βCl)-NHMe (**1**) in DMSO-d_6.

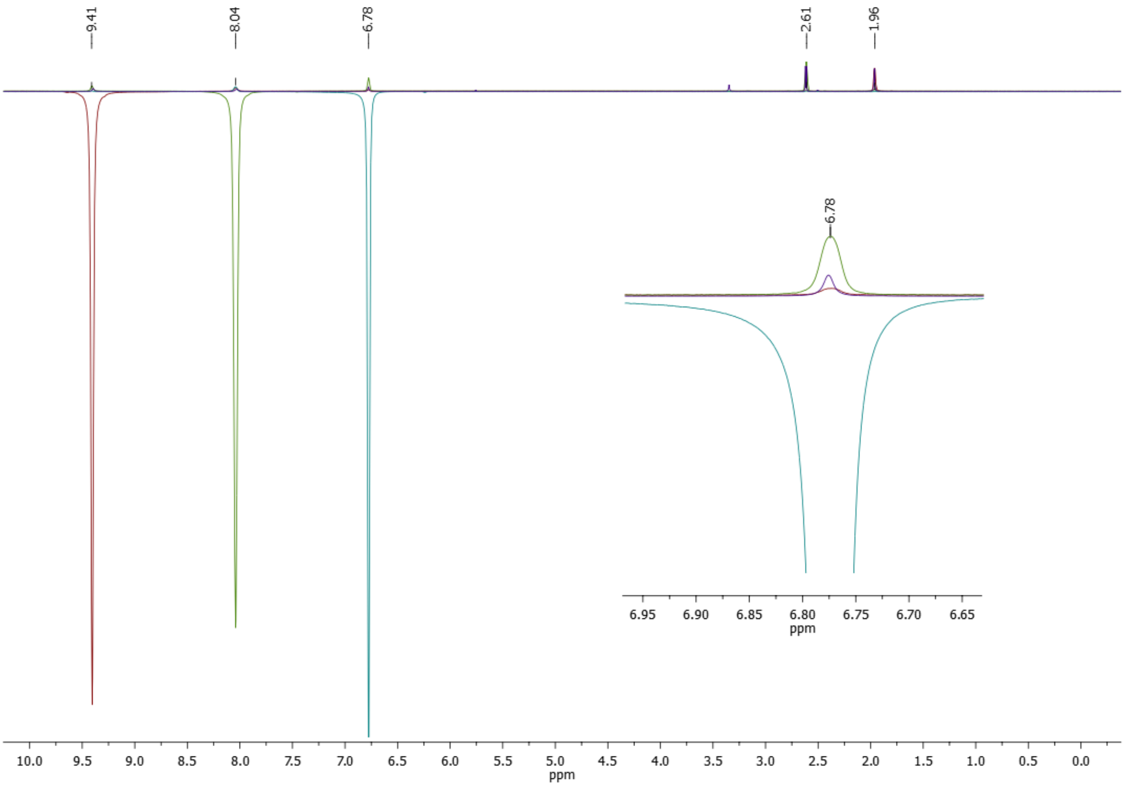

Figure 20S. ^{1}H NMR 1D-NOE spectra obtained by selective excitation of the N-terminal amide H atom (red spectrum), the C-terminal amide H atom (green spectrum) and the side chain H atom (blue spectrum) of Ac-(*Z*)-ΔAla(βCl)-NHMe (**1**) in DMSO-d_6.

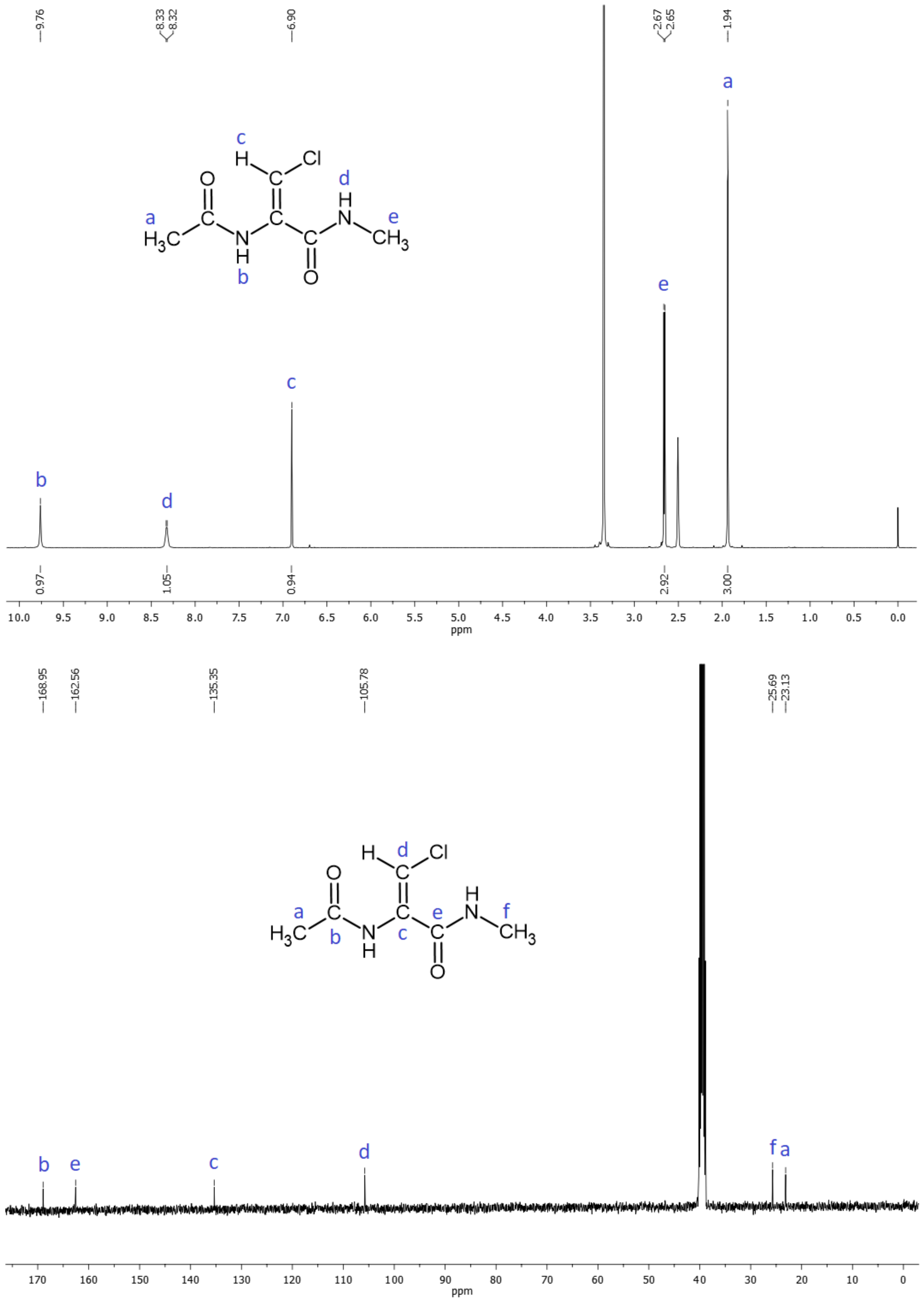

Figure 21S. ^{1}H and ^{13}C NMR spectra of Ac-(*E*)-ΔAla(βCl)-NHMe (**2**) in DMSO-d_6.

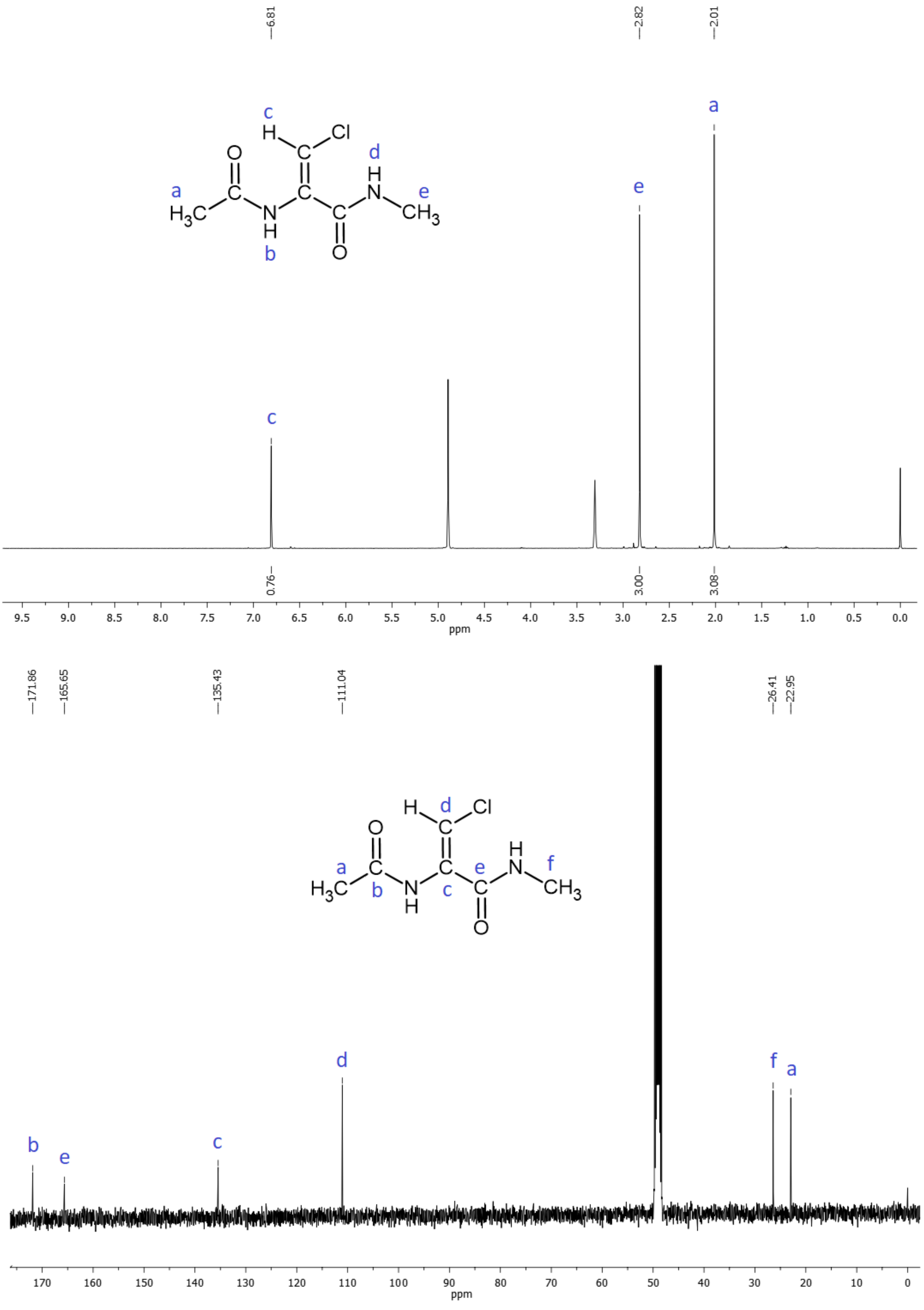

Figure 22S. ^{1}H and ^{13}C NMR spectra of Ac-(*E*)-ΔAla(βCl)-NHMe (**2**) in CD_3OD.

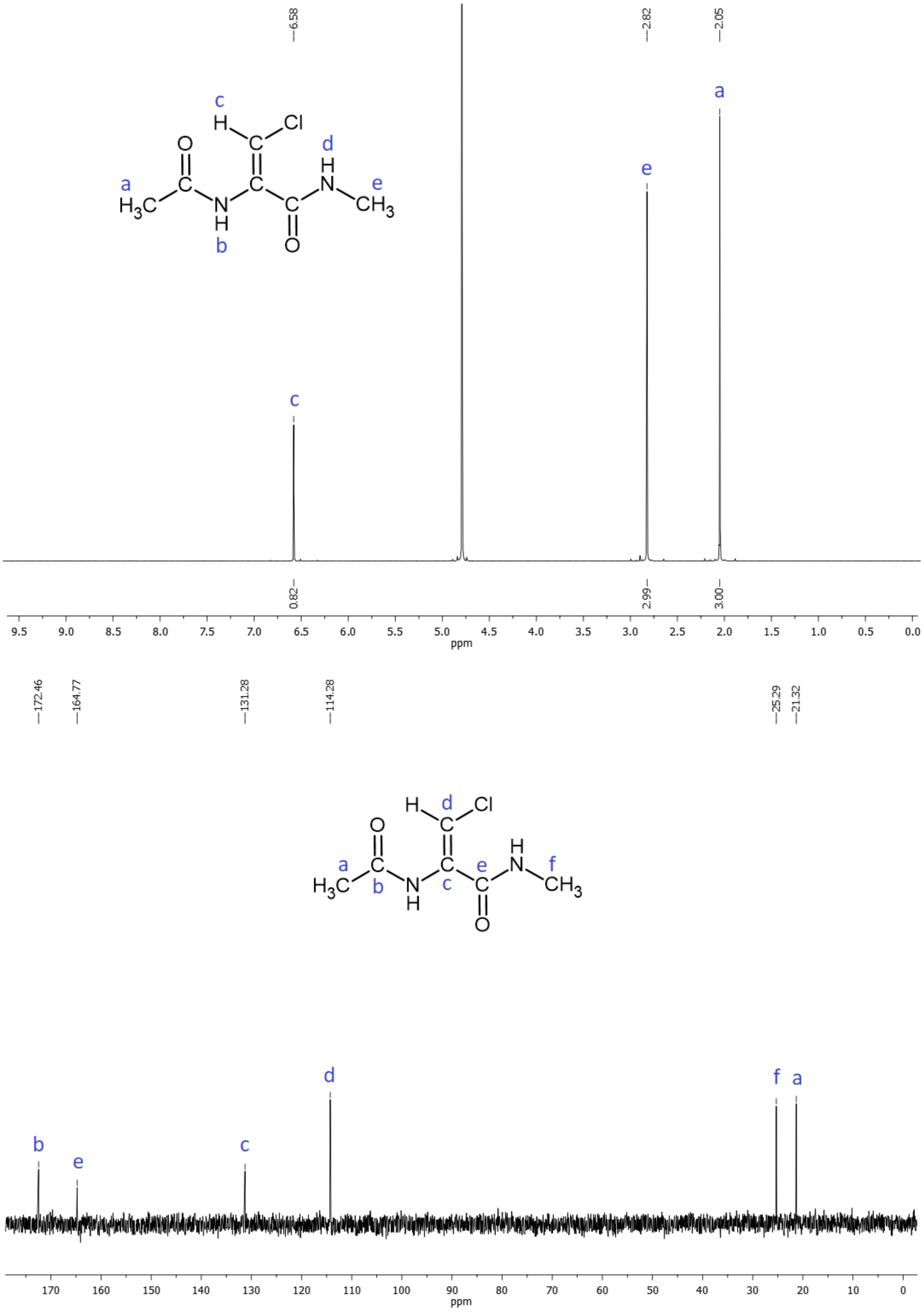

Figure 23S. ^{1}H and ^{13}C NMR spectra of Ac-(*E*)-ΔAla(βCl)-NHMe (**2**) in D_2O.

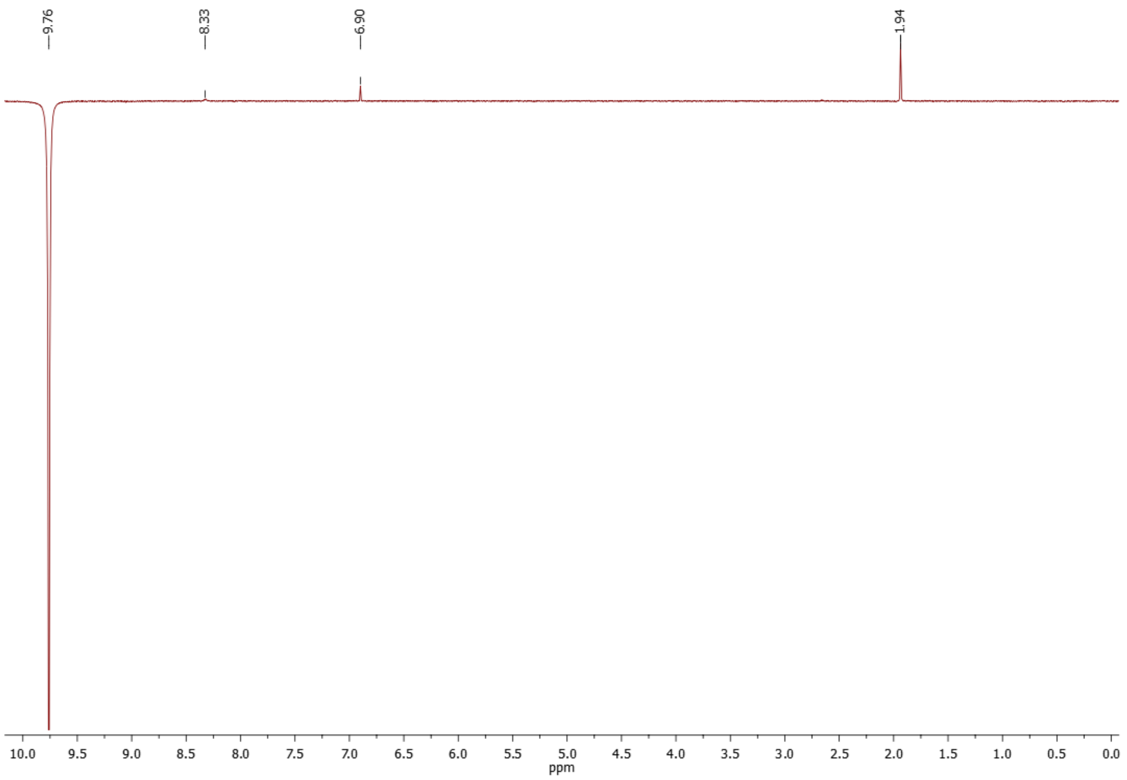

Figure 24S. ^{1}H NMR 1D-NOE spectra obtained by selective excitation of the N-terminal amide H atom of Ac-(*E*)-ΔAla(βCl)-NHMe (**2**) in DMSO-d_6.

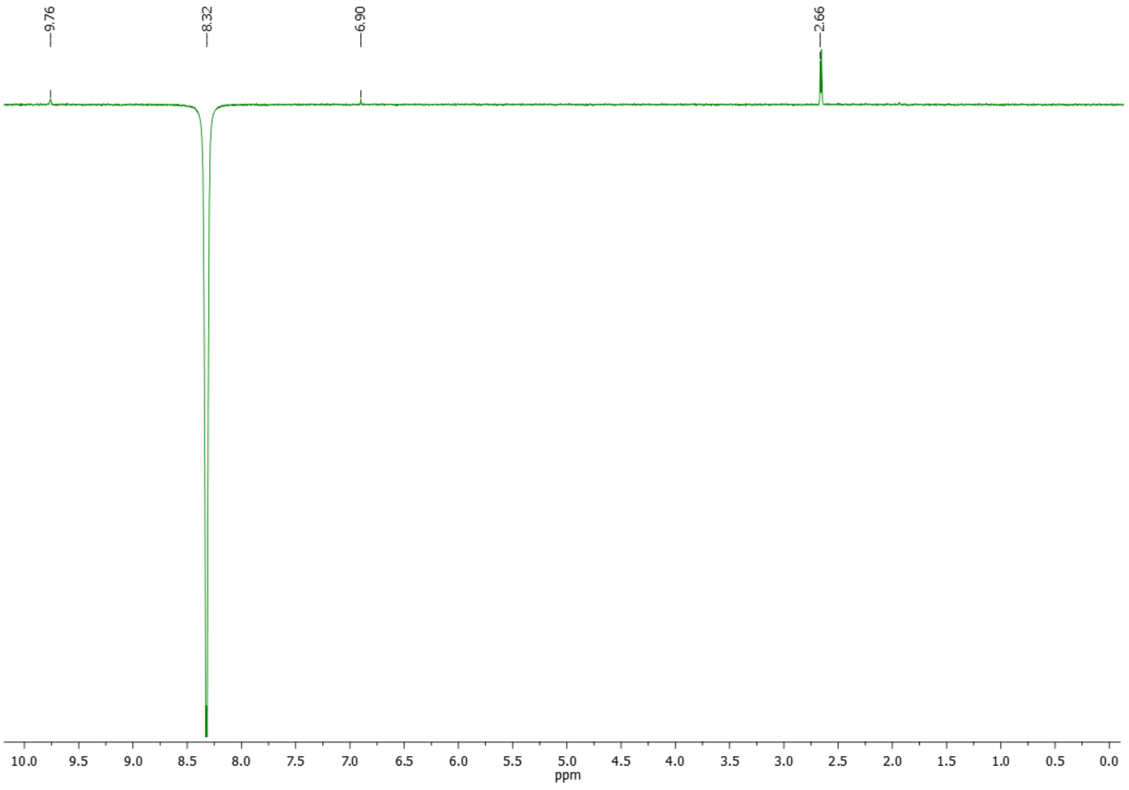

Figure 25S. ^{1}H NMR 1D-NOE spectra obtained by selective excitation of the C-terminal amide H of Ac-(*E*)-ΔAla(βCl)-NHMe (**2**) in DMSO-d_6.

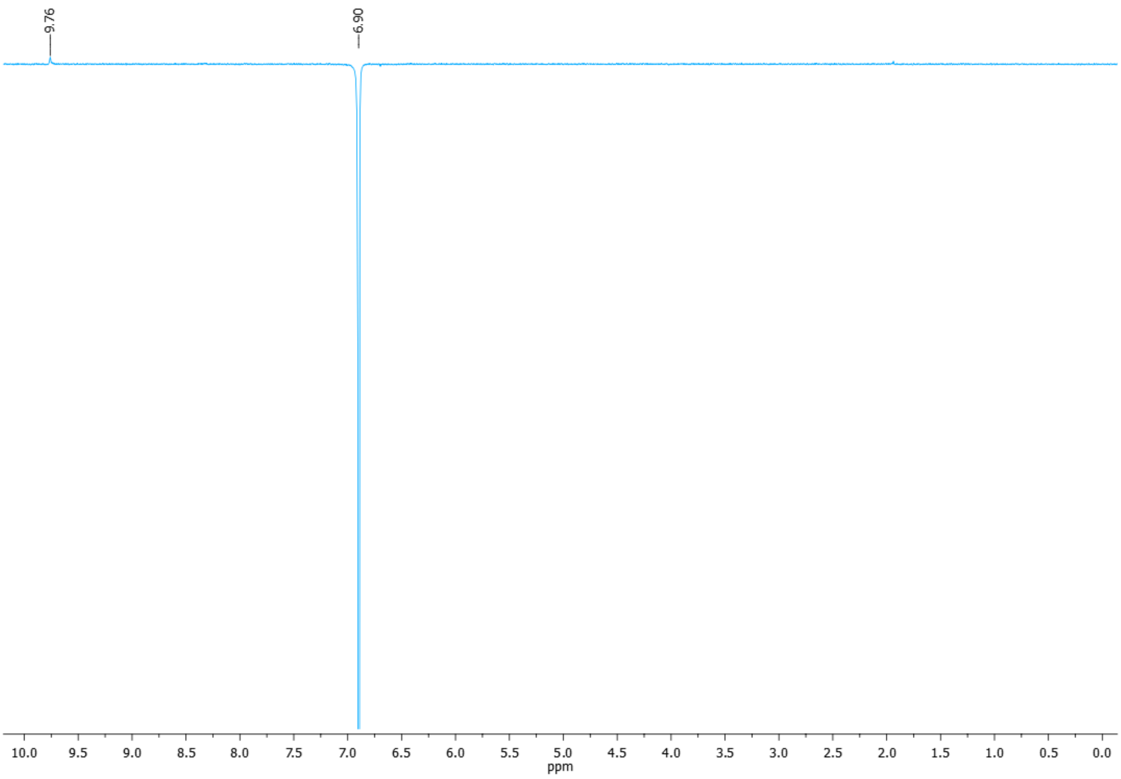

Figure 26S. ^{1}H NMR 1D-NOE spectra obtained by selective excitation of the side chain H atom of Ac-(*E*)-ΔAla(βCl)-NHMe (**2**) in DMSO-d_6.

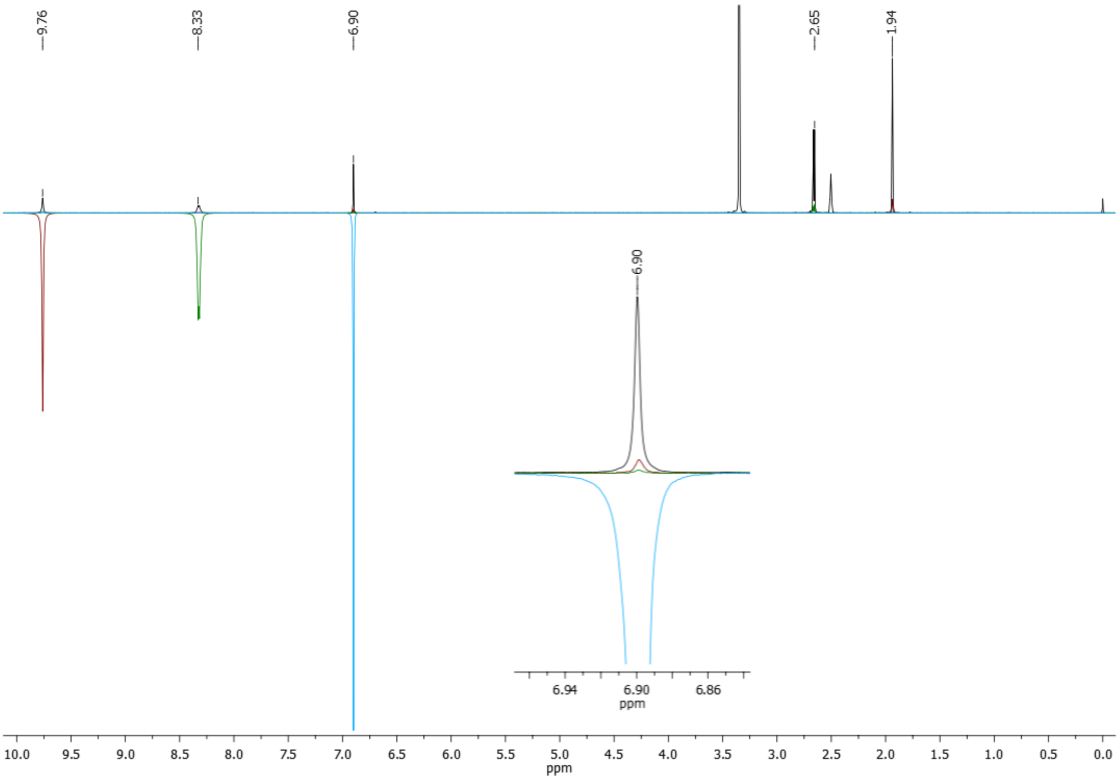

Figure 27S. ^{1}H NMR 1D-NOE spectra obtained by selective excitation of the N-terminal amide H atom (red spectrum), the C-terminal amide H atom (green spectum) and the side chain H atom (blue spectrum) of Ac-(*E*)-ΔAla(βCl)-NHMe (**2**) in DMSO-d_6.

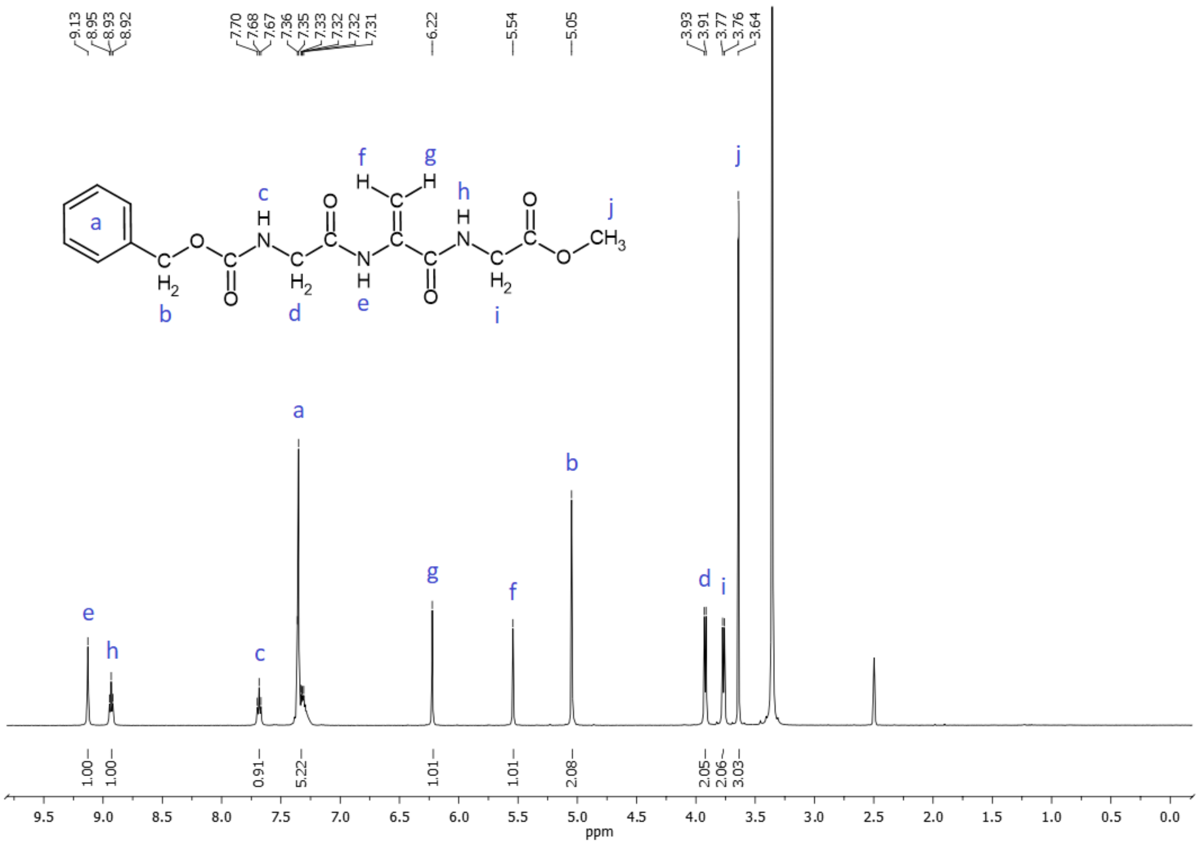

Figure 28S. ^{1}H NMR spectrum of Cbz-Gly-ΔAla-Gly-OMe in DMSO-d_6.

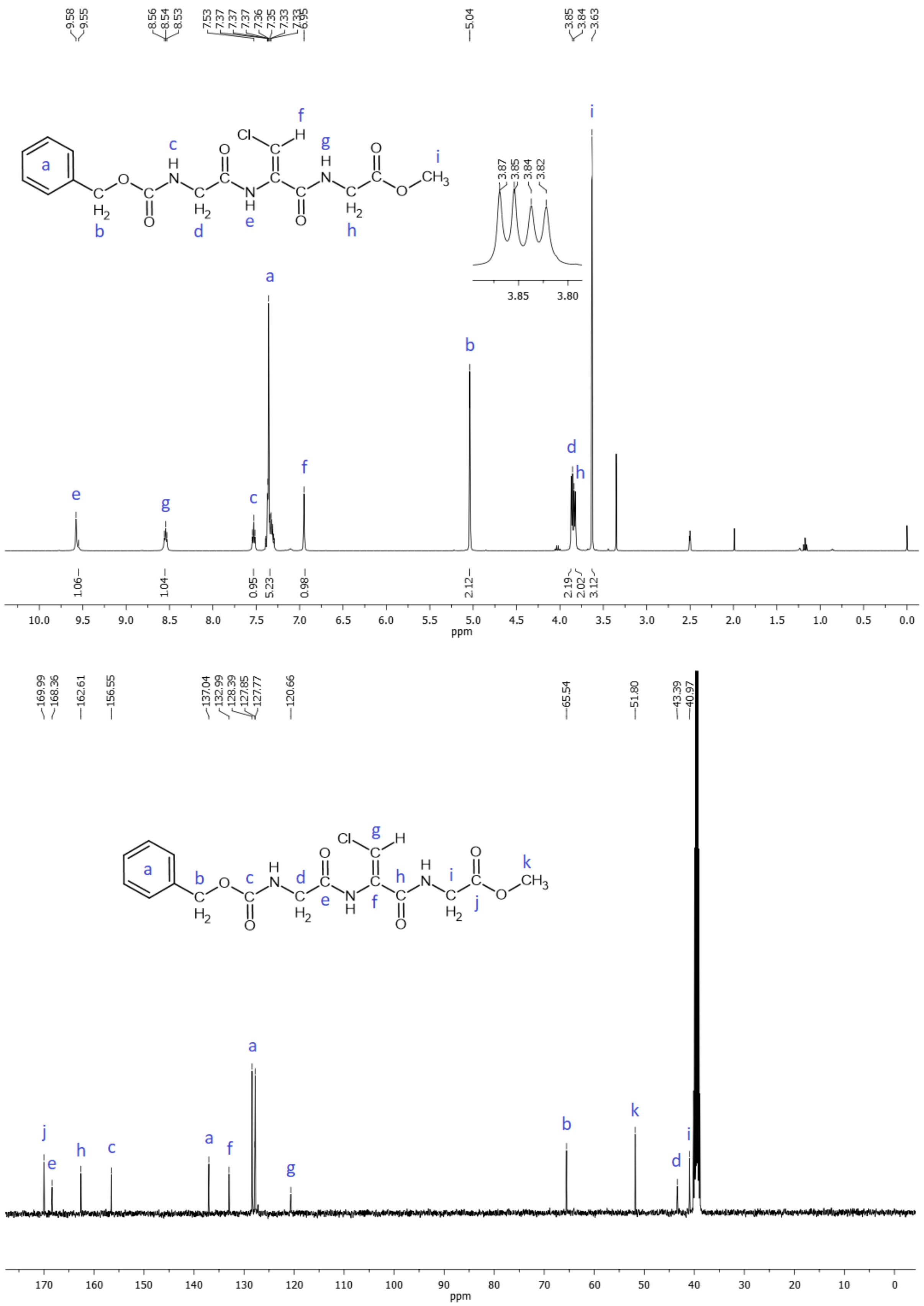

Figure 29S. ^{1}H and ^{13}C NMR spectra of Cbz-Gly-(*Z*)-ΔAla(βCl)-Gly-OMe (**3**) in DMSO-d_6.

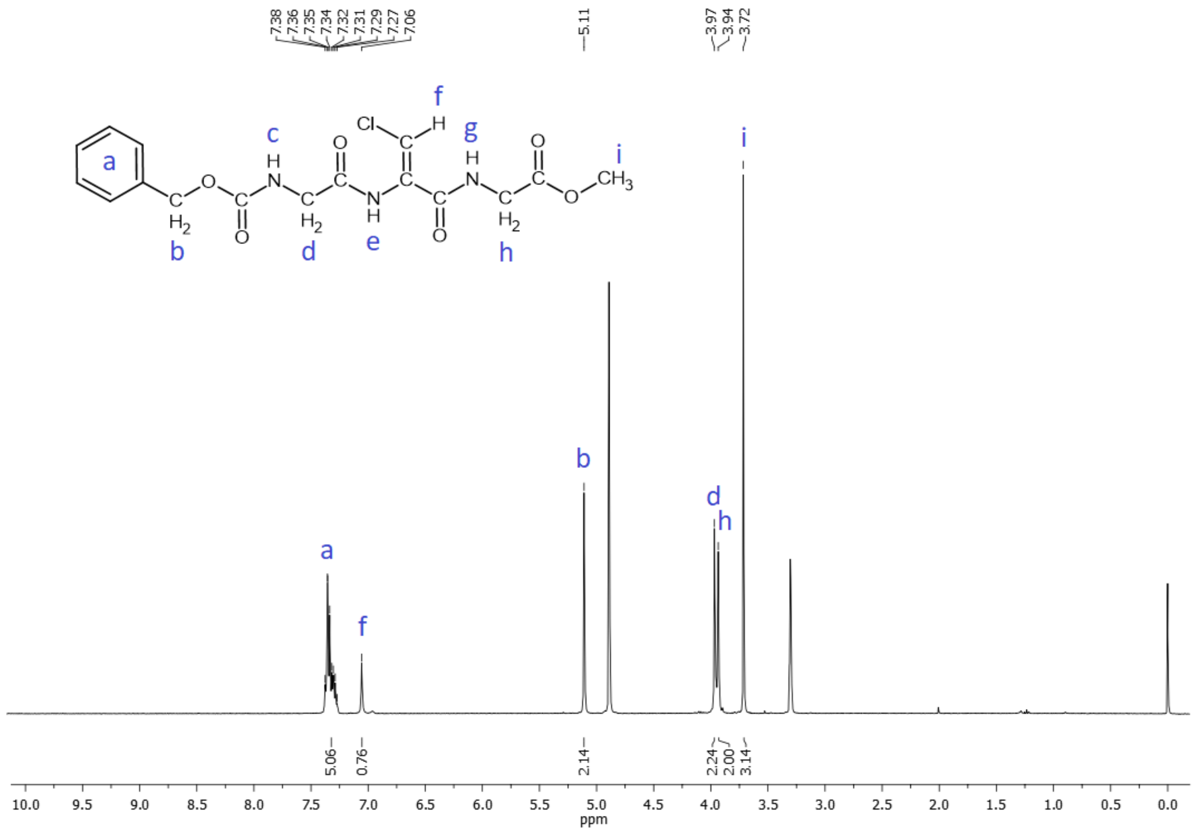

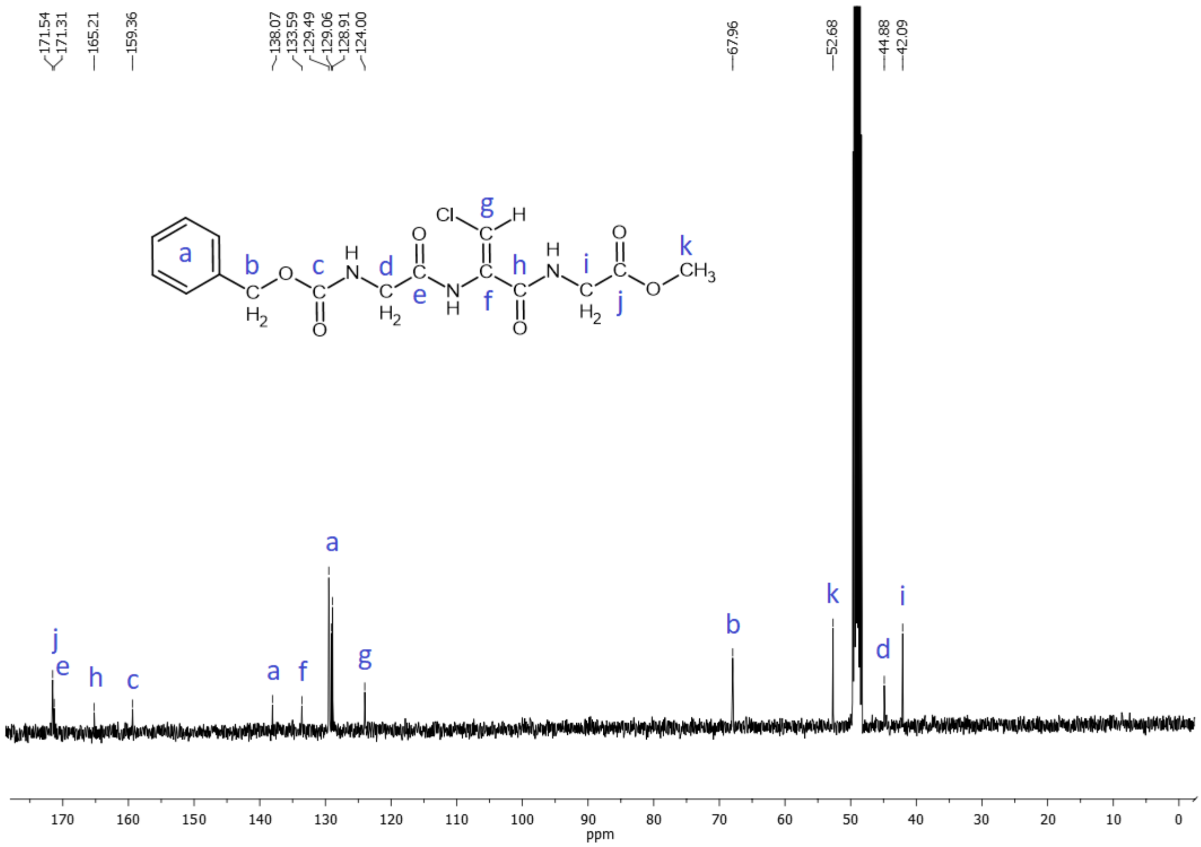

Figure 30S. ^{1}H and ^{13}C NMR spectra of Cbz-Gly-(*Z*)-ΔAla(βCl)-Gly-OMe (**3**) in CD_3OD.

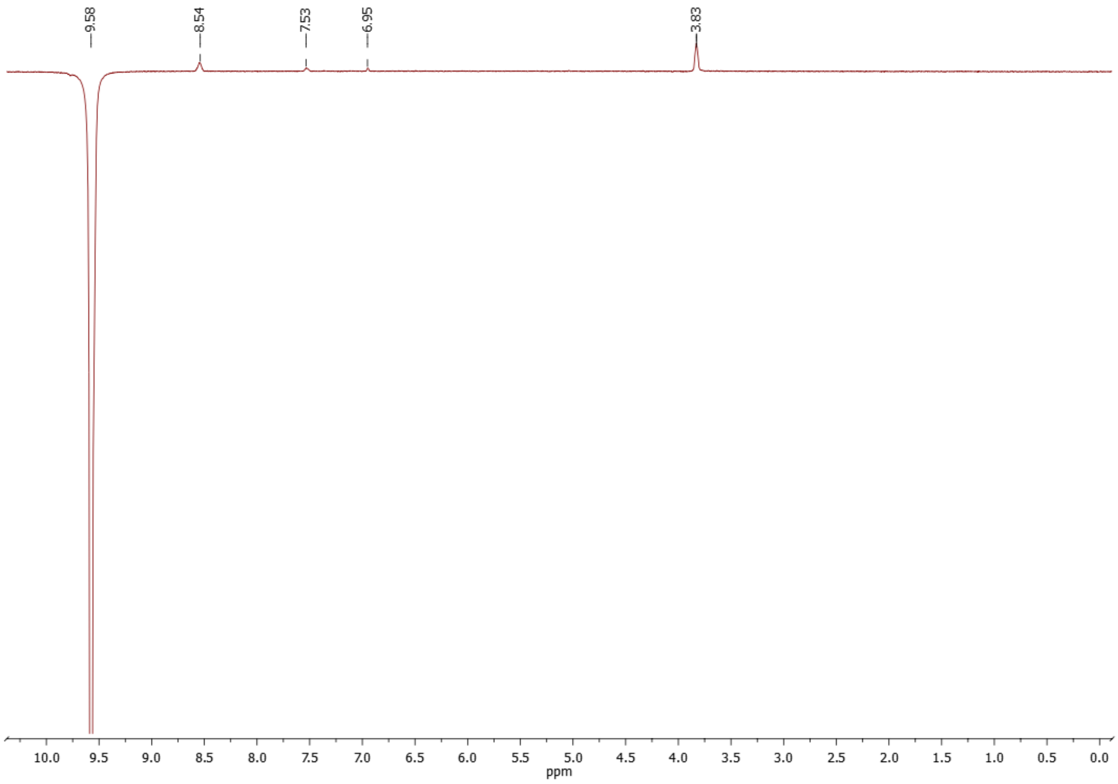

Figure 31S. ^{1}H NMR 1D-NOE spectra obtained by selective excitation of the N-terminal amide H of Cbz-Gly-(*Z*)-ΔAla(βCl)-Gly-OMe (**3**) in DMSO-d_6.

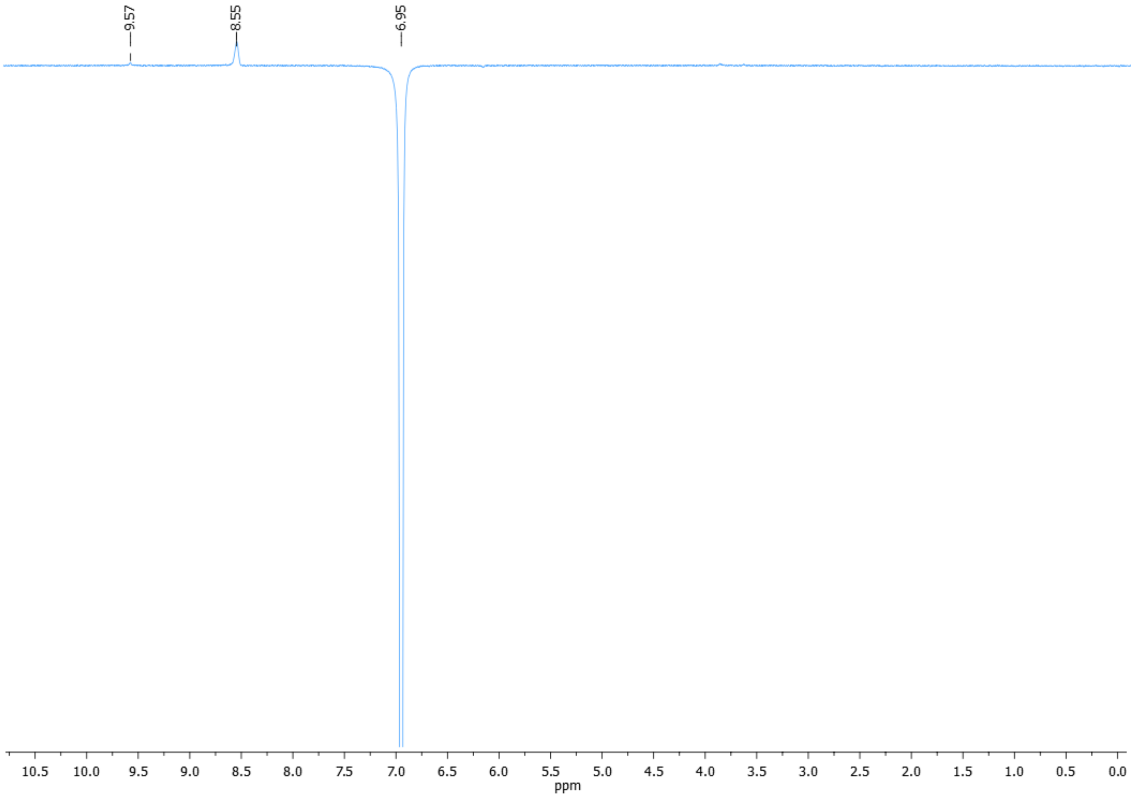

Figure 32S. ^{1}H NMR 1D-NOE spectra obtained by selective excitation side chain H atom (blue spectrum) of Cbz-Gly-(*Z*)-ΔAla(βCl)-Gly-OMe (**3**) in DMSO-d_6.

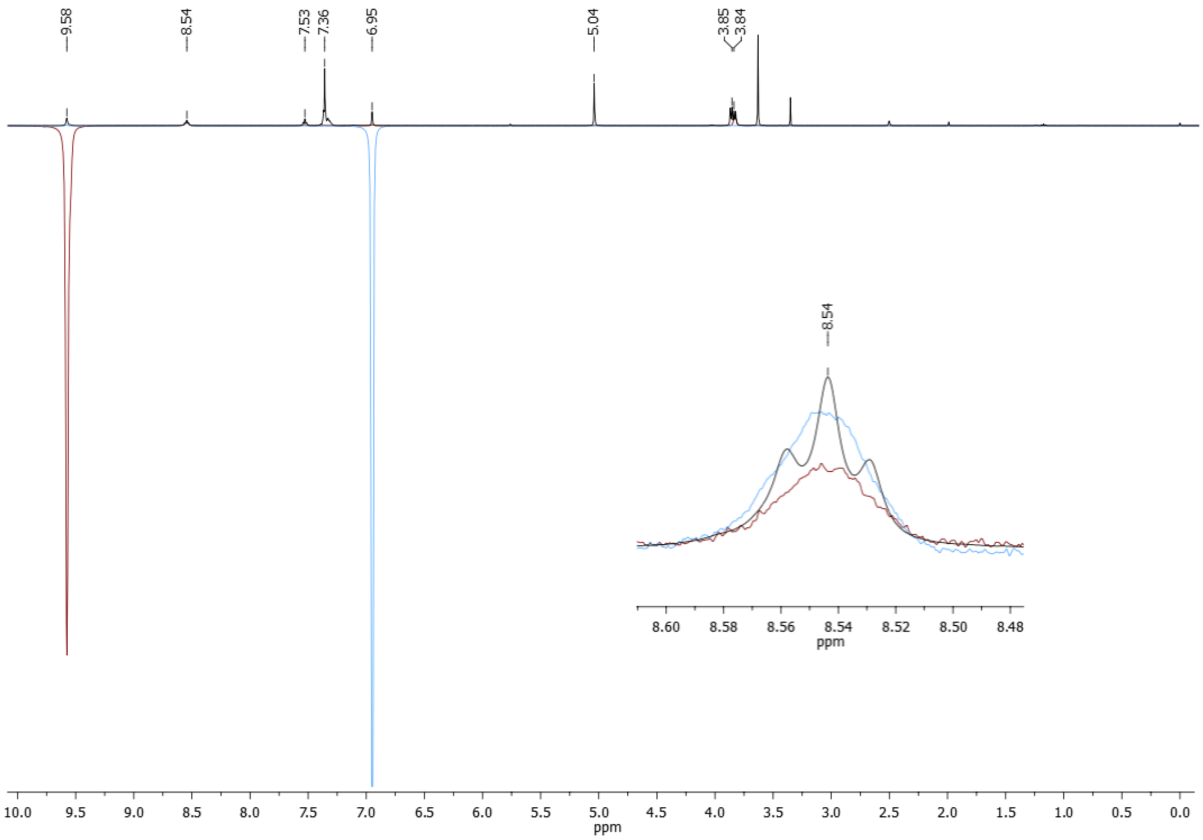

Figure 33S. ^{1}H NMR 1D-NOE spectra obtained by selective excitation of the N-terminal amide H atom (red spectrum) and the side chain H atom (blue spectrum) of Cbz-Gly-(*Z*)-ΔAla(βCl)-Gly-OMe (**3**) in DMSO-d_6.

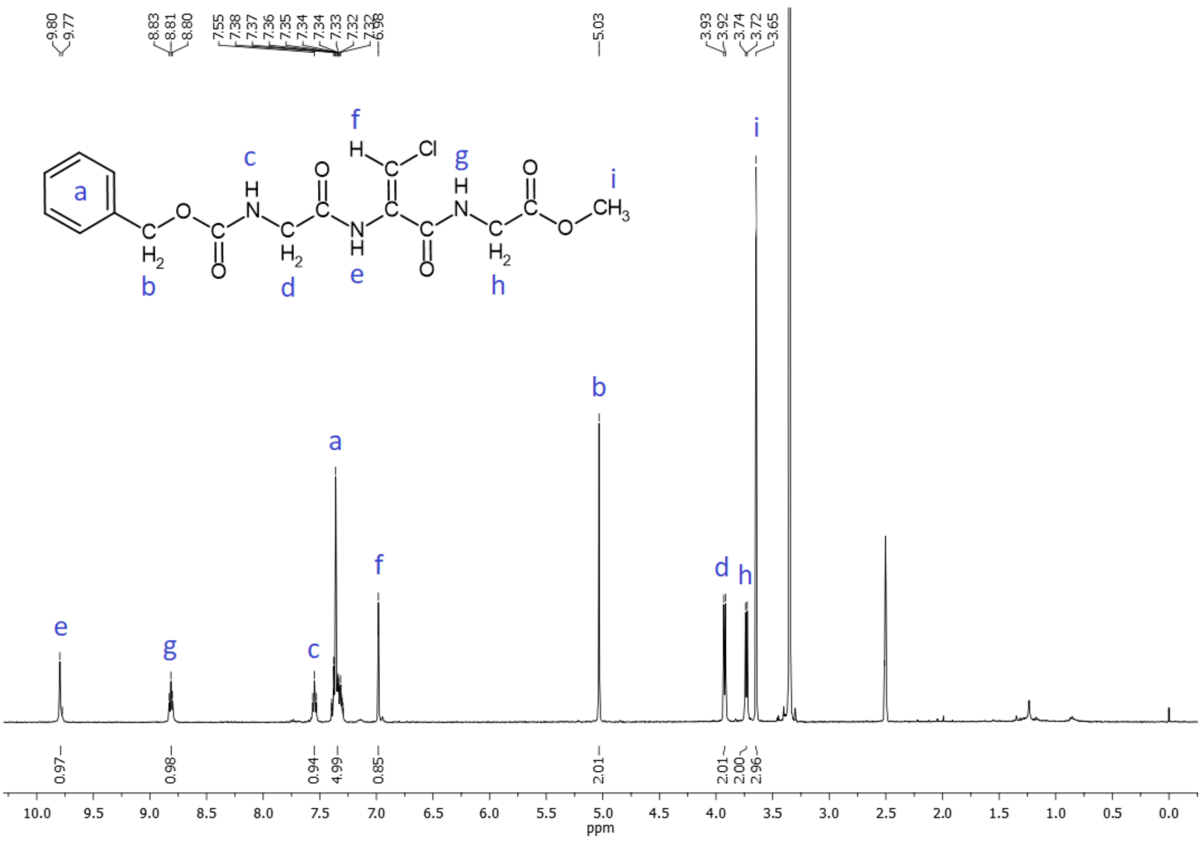

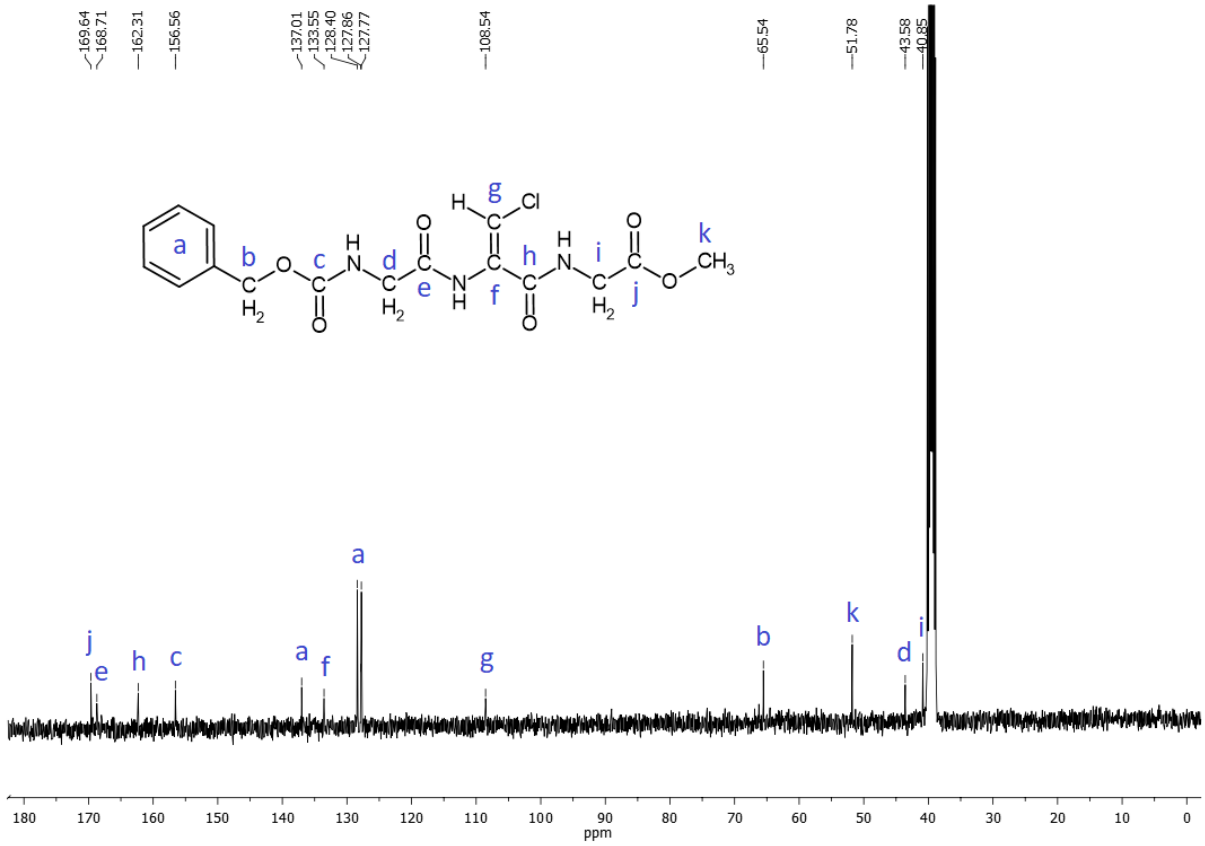

Figure 34S. ^{1}H and ^{13}C NMR spectra of Cbz-Gly-(*E*)-ΔAla(βCl)-Gly-OMe in DMSO-d_6.

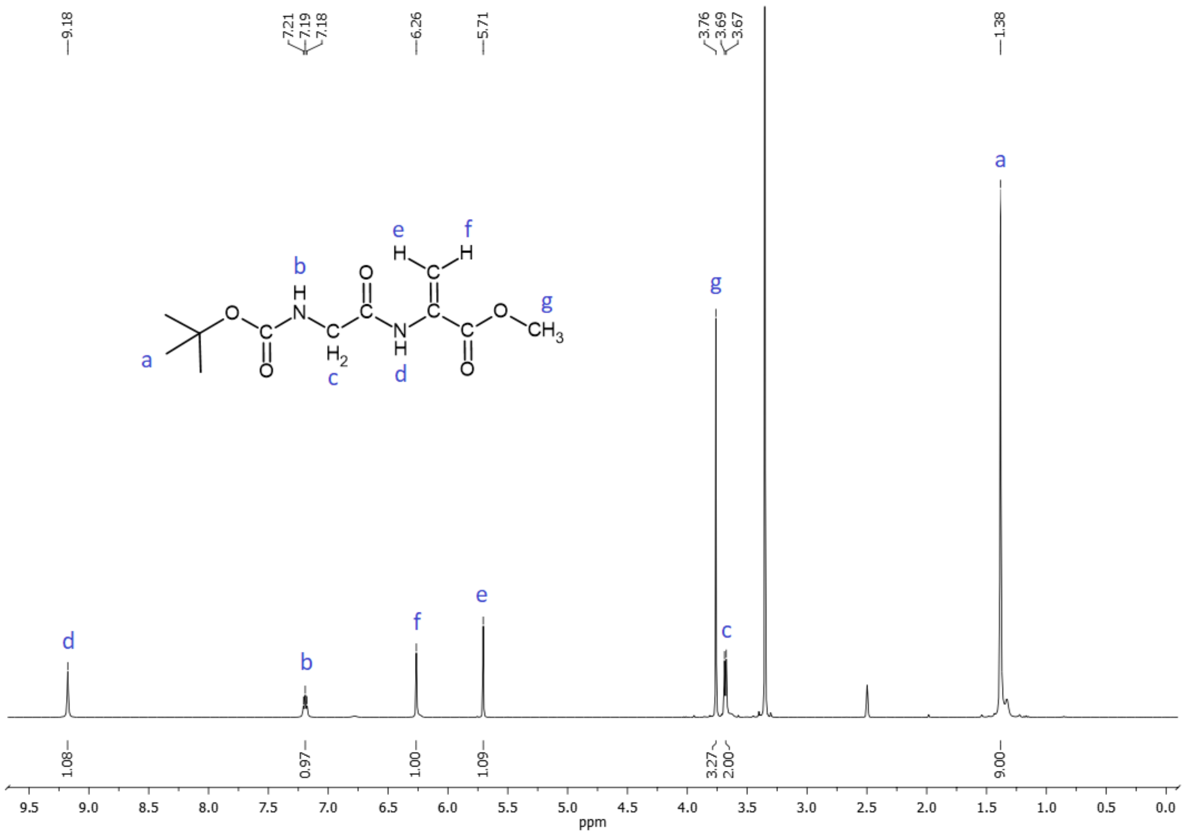

Figure 35S. ^{1}H spectrum of Boc-Gly-ΔAla-OMe in DMSO-d_6.

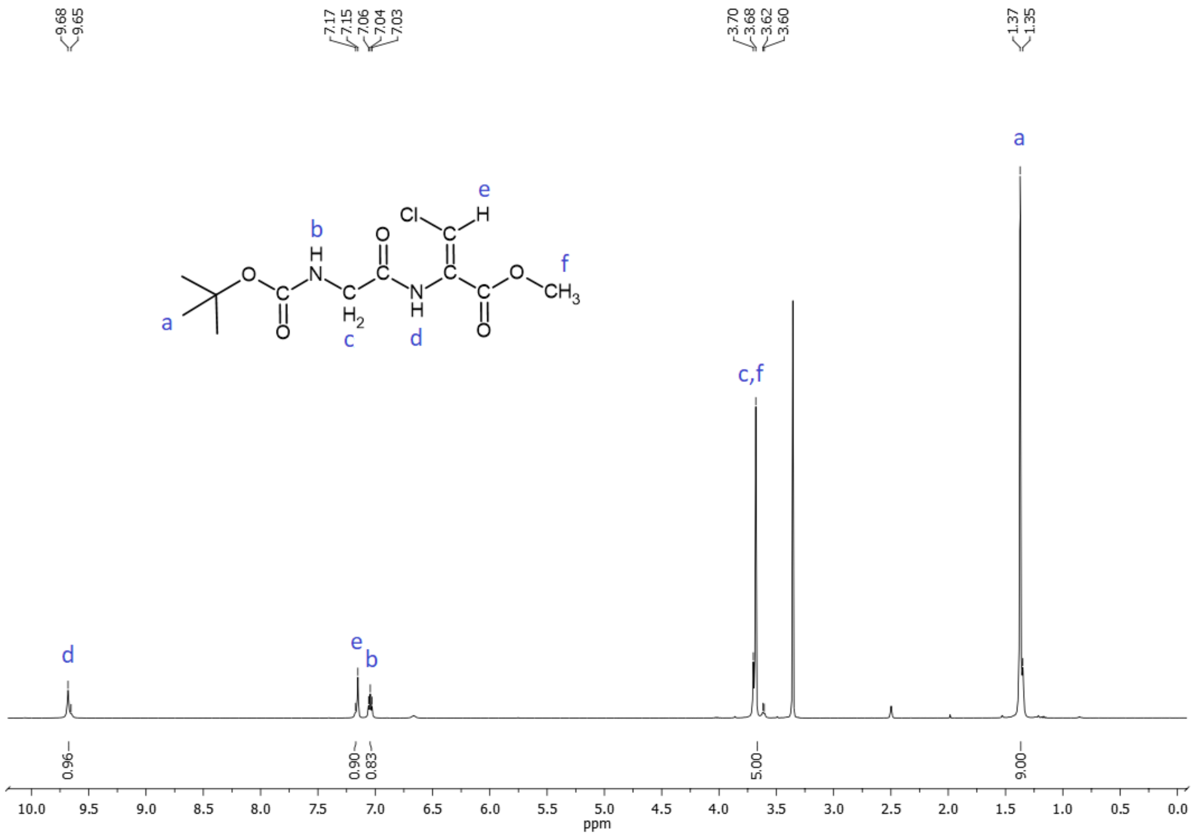

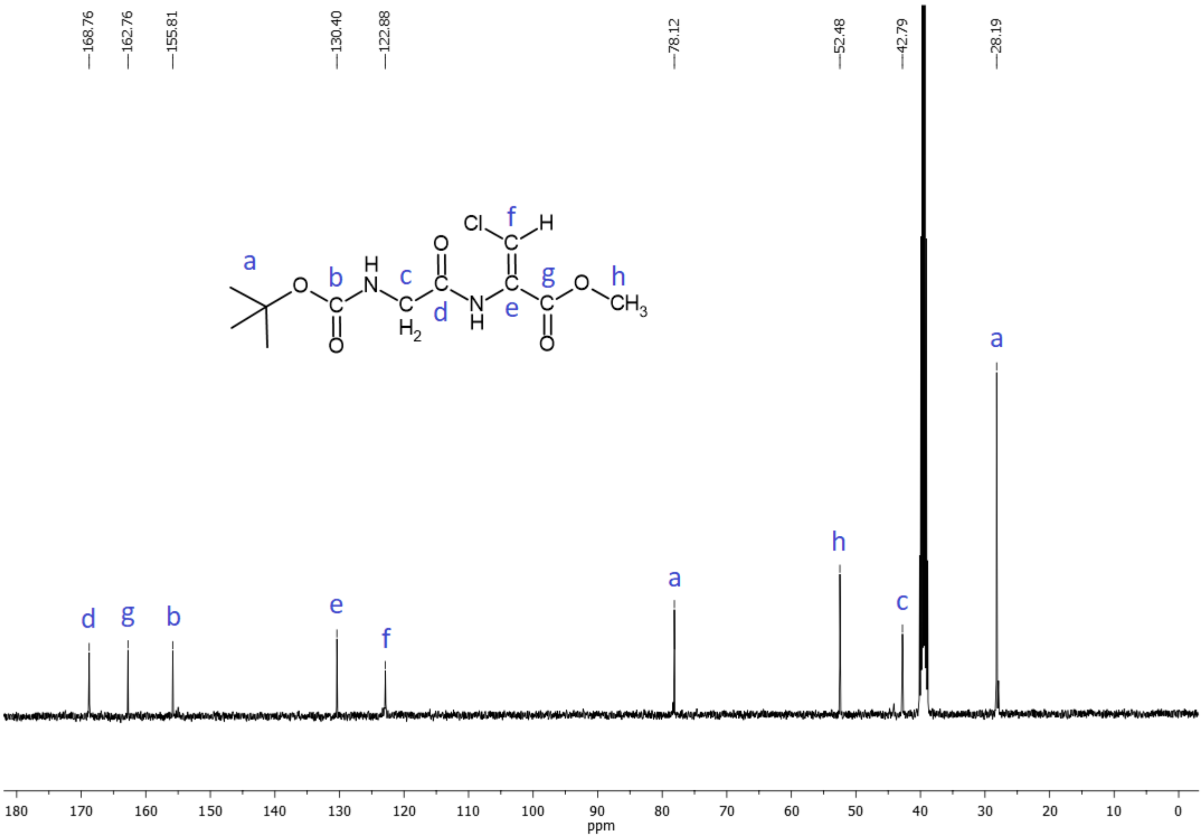

Figure 36S. ^{1}H and ^{13}C NMR spectra of Boc-Gly-(*Z*)-ΔAla(βCl)-OMe (**4**) in DMSO-d_6.

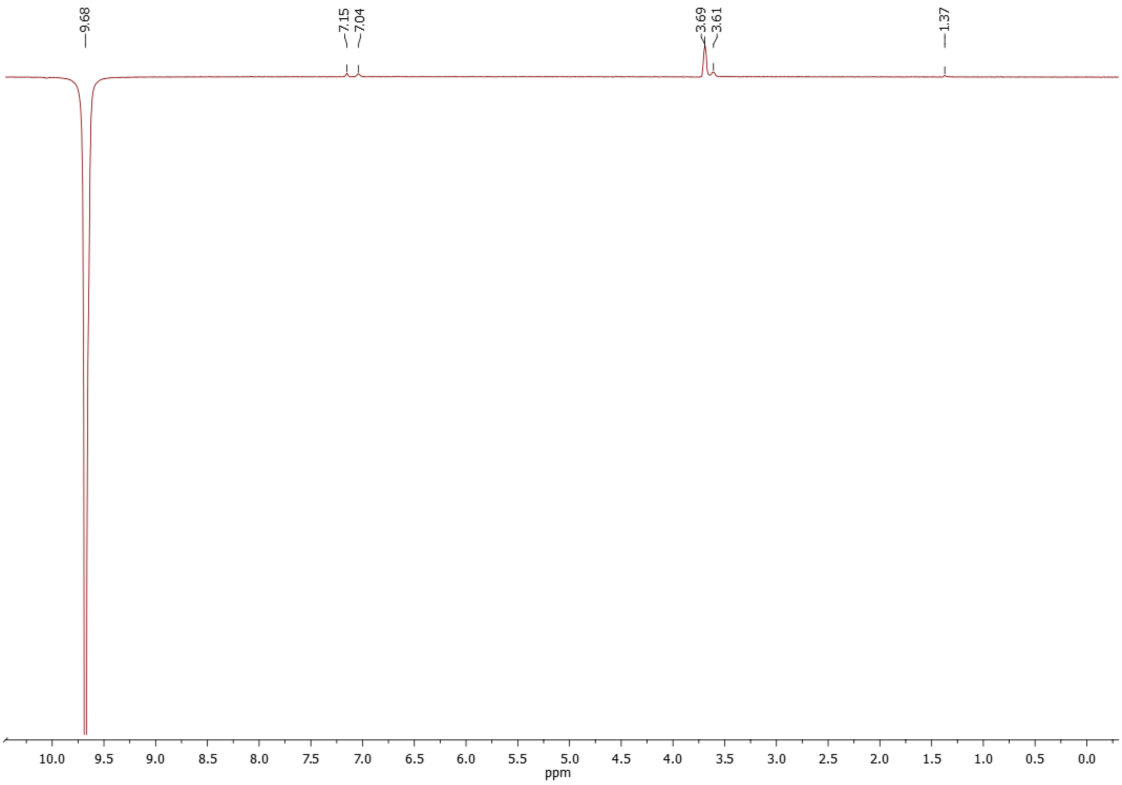

Figure 37S. ^{1}H NMR 1D-NOE spectra obtained by selective excitation of the N-terminal amide H atom of Boc-Gly-(*Z*)-ΔAla(βCl)-OMe (**4**) in DMSO-d_6.

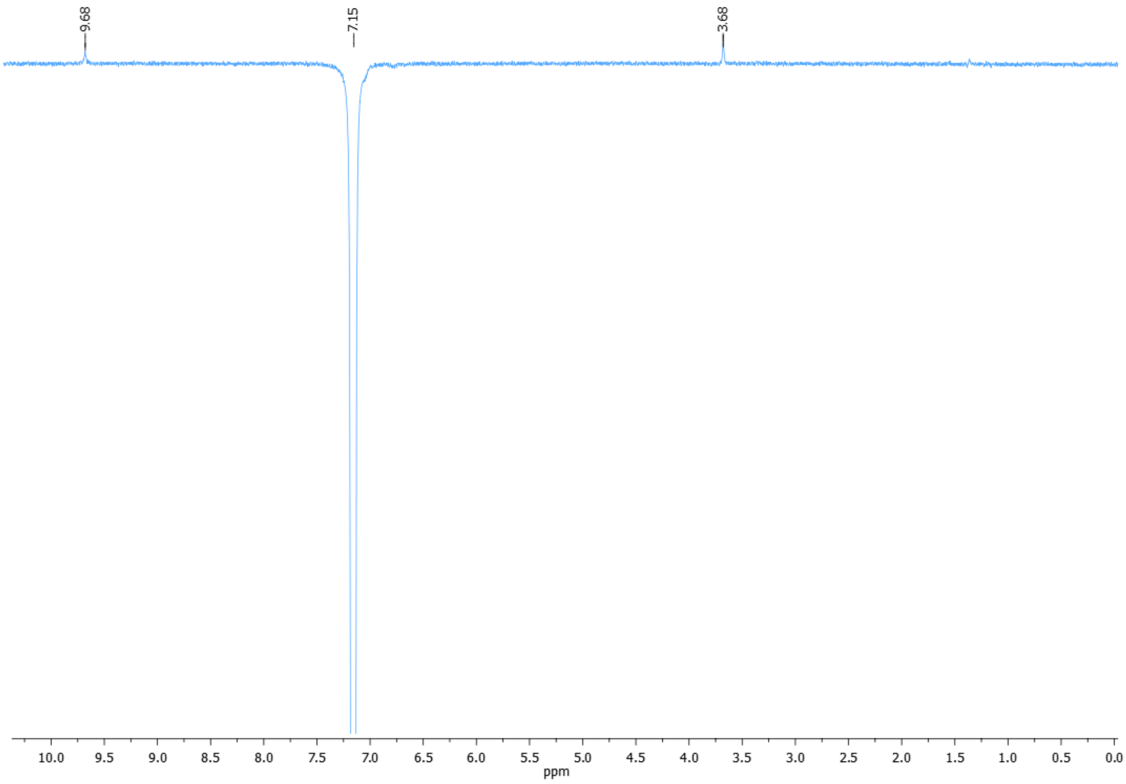

Figure 38S. ^{1}H NMR 1D-NOE spectra obtained by selective excitation of the side chain H atom of Boc-Gly-(*Z*)-ΔAla(βCl)-OMe (**4**) in DMSO-d_6.

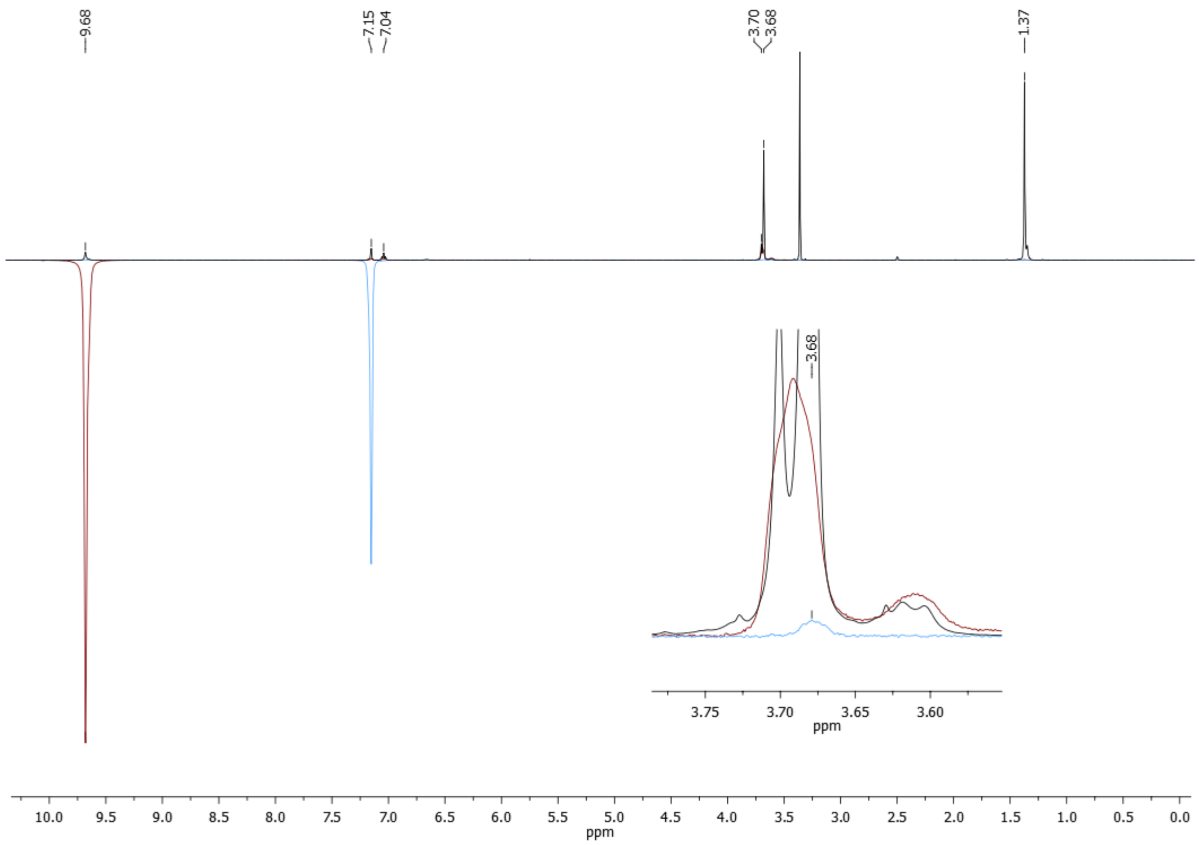

Figure 39S. ^{1}H NMR 1D-NOE spectra obtained by selective excitation of the N-terminal amide H atom (red spectrum) and the side chain H atom (blue spectrum) of Boc-Gly-(*Z*)-ΔAla(βCl)-OMe (**4**) in DMSO-d_6.

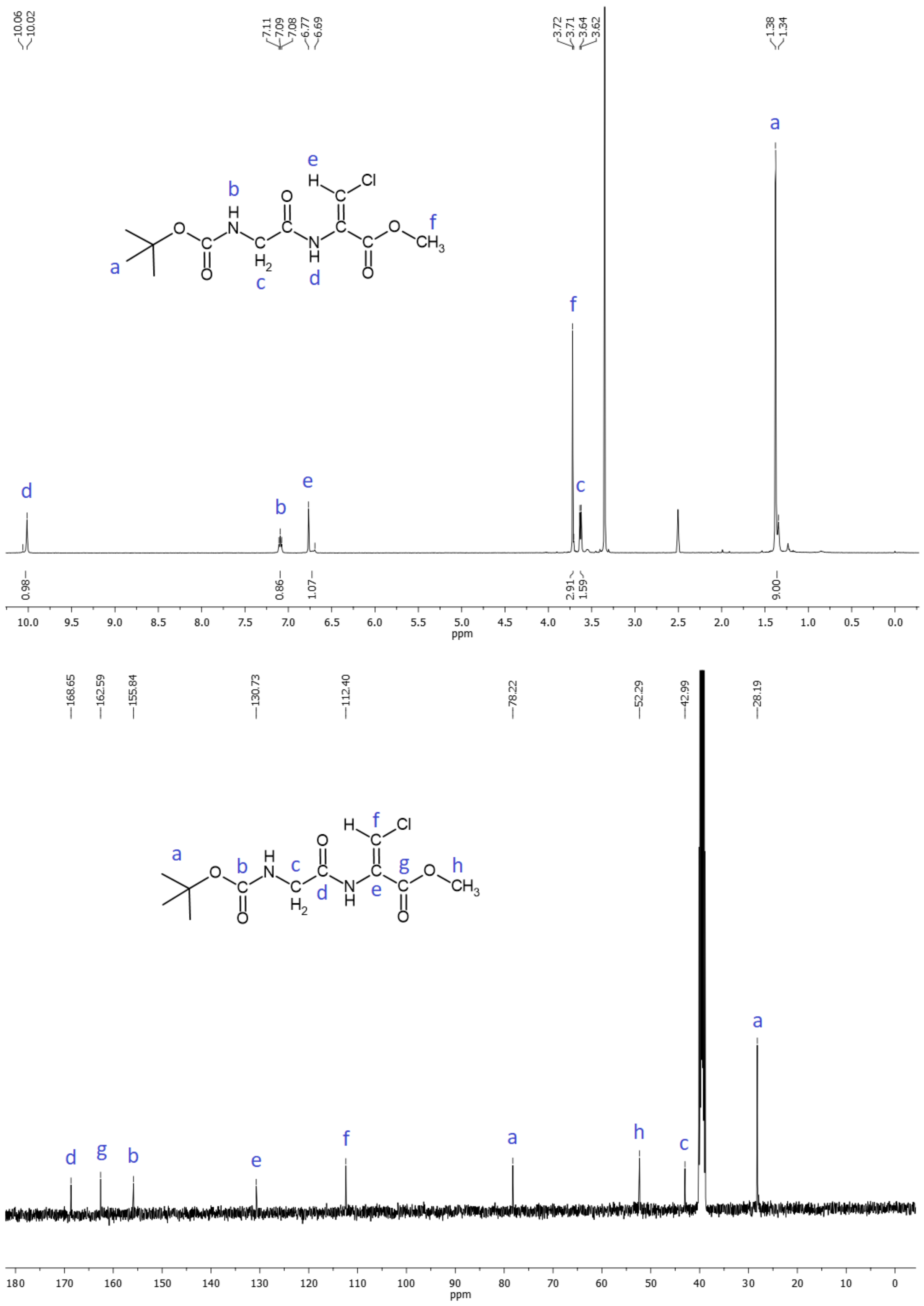

Figure 40S. ^{1}H and ^{13}C NMR spectra of Boc-Gly-(*E*)-ΔAla(βCl)-OMe in DMSO-d_6.

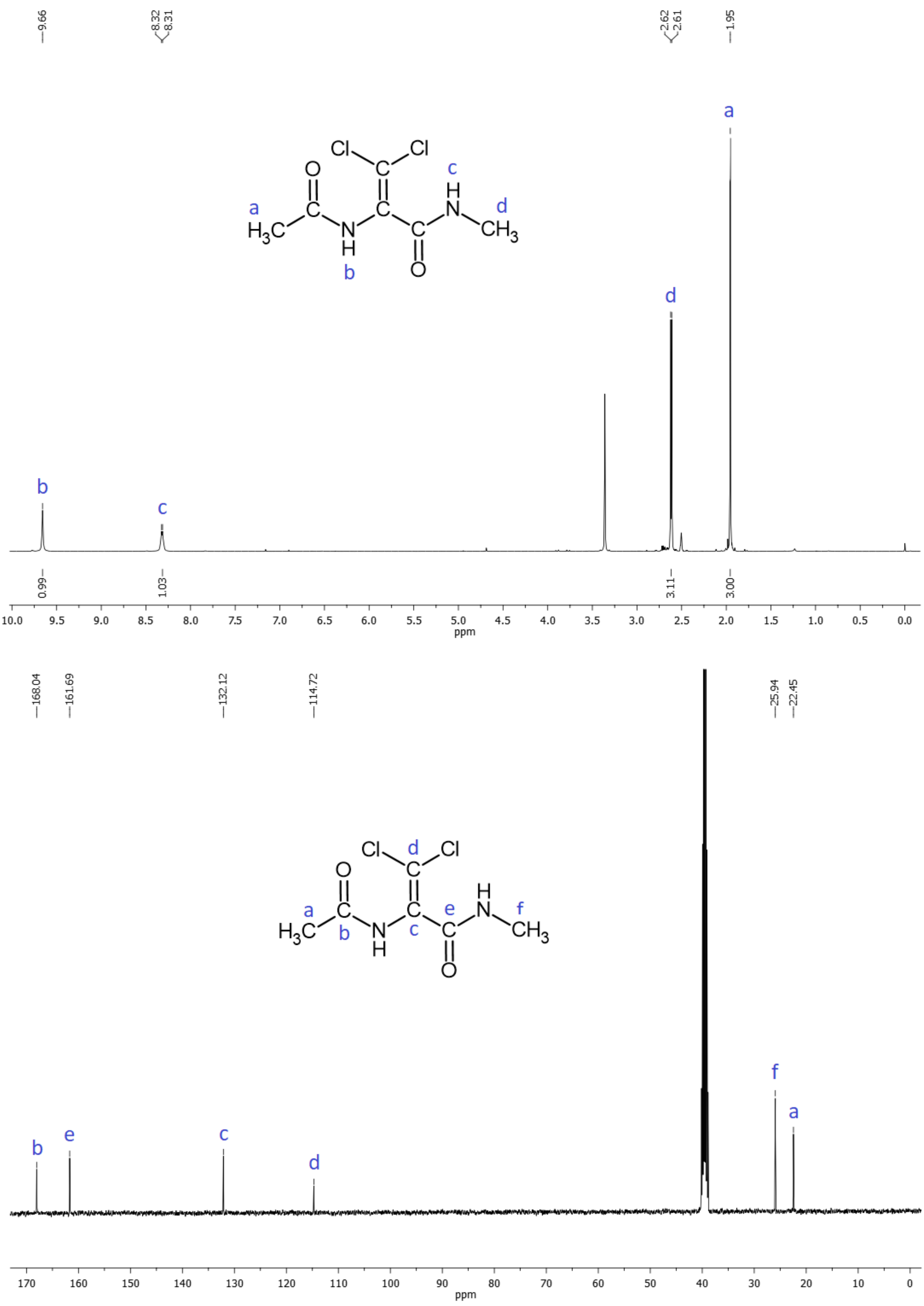

Figure 41S. ^{1}H and ^{13}C NMR spectra of Ac-ΔAla(βCl$_2$)-NHMe (**5**) in DMSO-d_6.
